# Association of Weight Loss Interventions With Changes in Biomarkers of Nonalcoholic Fatty Liver Disease: A Systematic Review and Meta-analysis

**DOI:** 10.1001/jamainternmed.2019.2248

**Published:** 2019-07-01

**Authors:** Dimitrios A. Koutoukidis, Nerys M. Astbury, Kate E. Tudor, Elizabeth Morris, John A. Henry, Michaela Noreik, Susan A. Jebb, Paul Aveyard

**Affiliations:** 1 Nuffield Department of Primary Care Health Sciences, University of Oxford, Oxford, United Kingdom; 2 National Institute for Health Research Oxford Biomedical Research Centre, Oxford University Hospitals NHS Foundation Trust, Oxford, United Kingdom

## Abstract

**Importance:**

Nonalcoholic fatty liver disease (NAFLD) affects about 25% of adults worldwide and is associated with obesity. Weight loss may improve biomarkers of liver disease, but its implications have not been systematically reviewed and quantified.

**Objective:**

To estimate the association of weight loss interventions with biomarkers of liver disease in NAFLD.

**Data Sources:**

MEDLINE, Embase, PsycINFO, CINAHL, Cochrane, and Web of Science databases along with 3 trial registries were searched from inception through January 2019.

**Study Selection:**

Randomized clinical trials of people with NAFLD were included if they compared any intervention aiming to reduce weight (behavioral weight loss programs [BWLPs], pharmacotherapy, and surgical procedures) with no or lower-intensity weight loss intervention. The review followed the Preferred Reporting Items for Systematic Reviews and Meta-analyses (PRISMA) guidelines.

**Data Extraction and Synthesis:**

Two independent reviewers screened the studies, extracted the data, and assessed the risk of bias using the Cochrane tool. Pooled mean differences or odds ratios (ORs) were obtained from random-effects meta-analyses.

**Main Outcomes and Measures:**

Blood, radiologic, and histologic biomarkers of liver disease.

**Results:**

Twenty-two studies with 2588 participants (with a mean [SD] age of 45 [14] years and with approximately 66% male) were included. Fifteen studies tested BWLPs, 6 tested pharmacotherapy, and 1 tested a surgical procedure. The median (interquartile range) intervention duration was 6 (3-8) months. Compared with no or lower-intensity weight loss interventions, more-intensive weight loss interventions were statistically significantly associated with greater weight change (–3.61 kg; 95% CI, –5.11 to –2.12; *I^2^* = 95%). Weight loss interventions were statistically significantly associated with improvements in biomarkers, including alanine aminotransferase (–9.81 U/L; 95% CI, –13.12 to –6.50; *I^2^* = 97%), histologically or radiologically measured liver steatosis (standardized mean difference: –1.48; 95% CI, –2.27 to –0.70; *I^2^* = 94%), histologic NAFLD activity score (–0.92; 95% CI, –1.75 to –0.09; *I^2^* = 95%), and presence of nonalcoholic steatohepatitis (OR, 0.14; 95% CI, 0.04-0.49; *I^2^* = 0%). No statistically significant change in histologic liver fibrosis was found (–0.13; 95% CI, –0.54 to 0.27; *I^2^* = 68%). Twelve studies were at high risk of bias in at least 1 domain. In a sensitivity analysis of the 3 trials at low risk of bias, the estimates and precision of most outcomes did not materially change.

**Conclusions and Relevance:**

The trials, despite some heterogeneity, consistently showed evidence of the association between weight loss interventions and improved biomarkers of liver disease in NAFLD in the short to medium term, although evidence on long-term health outcomes was limited. These findings appear to support the need to change the clinical guidelines and to recommend formal weight loss programs for people with NAFLD.

## Key Points

**Question:**

Are weight loss interventions associated with changes in biomarkers of liver disease in people with nonalcoholic fatty liver disease?

**Findings:**

In this systematic review and meta-analysis of 22 randomized clinical trials with 2588 participants with nonalcoholic fatty liver disease, weight loss interventions were associated with clinically meaningful improvements in biomarkers of liver disease, although no evidence of changes in fibrosis was found.

**Meaning:**

Evidence appeared to support changing the current clinical guidelines and recommending formal weight loss programs to treat people with nonalcoholic fatty liver disease.

## Teaser

This systematic review and meta-analysis evaluates 22 randomized clinical trials including 2588 participants with nonalcoholic fatty liver disease (NAFLD) who underwent interventions to determine whether these interventions were associated with changes in NAFLD biomarkers.

## Introduction

Nonalcoholic fatty liver disease (NAFLD) represents a spectrum of diseases starting from excess fat in the liver (steatosis) that can progress to inflammation and fibrosis (nonalcoholic steatohepatitis [NASH]), advanced fibrosis, and cirrhosis. Worldwide, approximately 25% of adults have NAFLD and about 2% to 6% of adults have NASH.^1^ Approximately 50% to 75% of people with obesity also have NAFLD.^2^ Obesity is a factor in the pathogenesis of both initial steatosis and progression to NASH.^2^ It is associated with more severe forms of NAFLD and with worse prognosis.^3^

Nonalcoholic fatty liver disease is serious and costly because patients with NAFLD have a high risk for liver-related and cardiovascular morbidity and mortality.^4^ People with severe NAFLD have 2.5 times (95% CI, 1.78-3.75) higher incidence of cardiovascular disease compared with matched controls.^5^ Incidence of hepatocellular carcinoma associated with NAFLD has increased 10-fold in the past decades, and NASH is the second most important factor in liver transplant.^6,7^ Within NAFLD, fibrosis is the marker most associated with long-term outcomes. Advanced fibrosis confers a relative risk of liver events of 14 and mortality of 3.^8,9^

No licensed pharmacotherapy is currently available for NAFLD or NASH. Clinical guidelines around the world recommend physicians offer advice on lifestyle modification, which mostly includes weight loss through hypoenergetic diets and increased physical activity.^10,11,12,13,14^ However, whether clinicians provide advice and the type of advice they give vary greatly,^15^ and guidelines rarely specifically recommend treatment programs to support weight loss. Behavioral weight loss programs (BWLPs), weight loss pharmacotherapy, and bariatric surgery lead to weight loss and a favorable cardiometabolic profile, but their association with improvements in NAFLD is unclear.^16,17,18^ Largely based on observational data, the assumption is that weight loss improves NAFLD through reducing insulin resistance, inflammation, and oxidative stress. Previous reviews did not find sufficient trials to perform a meta-analysis^19^ or did not assess biomarkers of liver disease.^20^ Other reviews have included isoenergetic diets of varying macronutrient content and exercise-only trials that may confound the association between weight loss and NAFLD.^21,22^

The current systematic review and meta-analysis aimed to synthesize the data from randomized clinical trials (RCTs) of interventions for weight loss and to quantitatively analyze the likely implications of weight loss and improvements in glucose regulation for biomarkers of liver disease.

## Methods

The review protocol was prospectively registered (PROSPERO ID: CRD42018088882). The protocol was followed with no changes, and the review followed the Preferred Reporting Items for Systematic Reviews and Meta-analyses (PRISMA) guidelines.^23^

The review included RCTs on adults with an NAFLD diagnosis. Given the lack of an accepted definition for the diagnosis of NAFLD, we used the definition presented in each study, including, but not limited to, the presence of NASH.

We included interventions comprising BWLPs, pharmacotherapy, bariatric surgery, alone or in combination. Exercise or diet interventions that did not aim for weight loss were excluded. Studies in which a weight loss intervention was combined with another potential treatment for NAFLD, such as pioglitazone hydrochloride, were excluded because the effect of weight loss intervention was potentially confounded by additional effects of the medication on the pathogenesis of disease.

The comparator intervention was no or minimal weight loss support or a lower-intensity weight loss intervention. We defined the intensity of the program by the extent of behavioral support, prescribed energy deficit, or pharmacotherapy dose. In trials that compared pharmacotherapy with BWLPs, pharmacotherapy was a priori deemed the intervention and the BWLP the comparator. Trials that compared interventions of the same intensity (eg, comparison between diets with the same behavioral support and energy deficit but different macronutrient content) were excluded.

To be included, trials needed to report at least 1 biomarker of liver disease, including alanine aminotransferase (ALT), aspartate transaminase (AST), alkaline phosphatase (ALP), γ-glutamyltransferase (GGT), the Enhanced Liver Fibrosis score, the NAFLD fibrosis score, the Fatty Liver Index, liver stiffness, radiologically or histologically measured steatosis, inflammation, ballooning, fibrosis, and the NAFLD Activity Score (NAS). Weight and insulin-resistance markers (hemoglobin A_1c_, fasting glucose, fasting insulin, and the homeostatic model assessment for assessing insulin resistance [HOMA-IR] or equivalent) were considered as mediating variables. Secondary outcomes included any adverse events. Trials were included irrespective of the length of intervention or length of follow-up.

We searched MEDLINE, Embase, PsycINFO, CINAHL, Cochrane, and Web of Science databases and 3 trial registries from inception until January 2019 with no restrictions on language or publication date. The search strategy (eMethods in the Supplement) was created by an experienced librarian and has been published. We also hand-searched studies from systematic reviews of interventions in NAFLD.

Among 6 of us (D.A.K., N.M.A., K.E.T., E.M., J.A.H., and M.N.), 2 paired up at a time to independently screen each study’s title and abstract and full text using an online standardized tool.^24^ Two of us at a time also independently extracted the data using a predefined and prepiloted data extraction form and assessed the risk of bias using the Cochrane Risk of Bias tool.^25^ The data items prespecified in the published protocol were extracted. Discrepancies were resolved through discussion or referral to a third reviewer (D.A.K. or P.A.). We contacted study authors for additional data when required. We assessed publication bias with funnel plots.

We conducted a meta-analysis for all outcomes in which at least 2 comparisons were available, using random-effects models defined a priori given the heterogeneity in the interventions, study populations, and assessment of outcomes. All outcomes are summarized as difference in means with 95% CIs, except the presence of definite NASH, which is summarized as odds ratios (ORs). Steatosis was summarized as standardized mean difference because it was measured with 3 different techniques (histologic examination, ultrasonography, and magnetic resonance imaging). Statistical heterogeneity was assessed with the *I^2^* statistic. We judged the strength of evidence by the precision of the CIs, suggesting clinically relevant improvements, and the heterogeneity. Data were interpreted in light of changes in mediating variables. For example, studies in which the weight loss interventions did not lead to weight loss may also not have led to a difference in biomarkers of liver disease. For ease of interpretation, all forest plots arranged the studies in descending order of achieved weight change.

For 3-arm RCTs, we compared the participants in each of the 2 interventions against half of the participants in the control group. If data on the change in liver state and its SD were not provided, we estimated these following the methods described in the Cochrane Handbook.^25^ We estimated mean baseline weight if studies reported only body mass index (BMI) using mean height by sex for the specific country.^26^ Aiming to reduce bias in summary estimates, we included in the meta-analysis only studies that described when the outcome in question showed no significant change at follow-up. To do so, we assumed the follow-up value equaled the baseline value and that the 2 measures were correlated when estimating the SD of the change. We reported the analytical methods of each study and used the data as analyzed in the published studies, acknowledging the variation in the methods of dealing with missing data. All analyses were conducted in Review Manager, version 5.3 (Cochrane Community).

We ran 3 prespecified additional analyses: (1) an analysis including only studies with a low risk of bias, (2) a subgroup analysis to explore the implications of different types of interventions (BWLPs and pharmacotherapy), and (3) separate analyses for interventions compared with a lower-intensity intervention and for interventions compared with no or minimal weight loss intervention. We undertook a post hoc subgroup analysis comparing the studies that had a minimum cutoff to include only participants with overweight in their eligibility criteria against studies that enrolled participants irrespective of weight status. We undertook a post hoc analysis to examine changes in fibrosis to separate trials that included people with all stages of NAFLD from people with NASH because this is one of the US Food and Drug Administration criteria for licensing treatments for NASH.

## Results

Excluding duplicates, 2096 titles or abstracts were screened and 221 full-text articles were assessed. Most studies were excluded because the intervention or comparator did not meet the inclusion criteria. Twenty-two studies evaluating 26 interventions were included, with 20 full-text articles and 2 conference abstracts only^27,28^ (PRISMA flowchart in eFigure 1 in the Supplement).

Overall, 2588 participants were included in the analyses. Six trials were conducted in middle-income countries,^29,30,31,32,33,34^ and the remaining trials were in high-income countries.^27,35,36,37,38,39,40,41,42,43,44,45,46,47,48^ The location was unclear for 1 study.^28^ Sixteen studies recruited participants with any stage of NAFLD,^27,29,30,31,32,33,34,36,37,39,40,42,43,46,47,48^ and 6 recruited only participants with NASH.^28,35,38,41,44,45^ Seven studies recruited participants regardless of BMI,^31,32,33,40,46,47,48^ whereas 15 of 22 recruited only people above a minimum BMI (median cutoff, 25). Among participants, approximately 66% were male and the mean (SD) age was 45 (14) years, the mean (SD) BMI was 33.7 (10.7), and about 7% had type 2 diabetes (4 studies did not report on diabetes status and 3 did not report on sex; thus, no exact numbers were given).

Six studies tested BWLPs against usual care,^29,31,32,35,36,37^ 9 tested BWLPs against a lower-intensity BWLP,^27,29,33,34,39,40,42,45,46,47^ 2 tested pharmacotherapy against placebo,^28,38^ 1 tested pharmacotherapy against a BWLP,^43^ 3 tested pharmacotherapy with a BWLP against either a BWLP or placebo,^30,41,48^ and 1 tested a surgical procedure with a BWLP against a BWLP.^44^ The Table presents the key characteristics of the included studies with additional detail provided in eTables 1 and 2 in the Supplement. Most BWLPs included both an energy-restricted diet and an exercise component; the pharmacotherapy included orlistat, liraglutide, or sibutramine hydrochloride; and the 1 surgical trial examined the implication of placing a gastric balloon. The median (interquartile range [IQR]) intervention duration was 6 (3-8) months, and all trials examined outcomes at intervention completion.

**Table.** Characteristics of Included Studies

Source	Disease	Total, No.	Participants, %		Duration, mo	Weight Loss Intervention	Comparison	Outcomes Measured
			Male	Female				
Dong et al,^32^ 2016 China	NAFLD	280	100	0	24	Diet and exercise	Usual care	ALT, AST, GGT, NFS, FLI, steatosis-US
Sun et al,^34^ 2012 China	NAFLD	1087	64	36	12	Diet and exercise	Minimal intervention	ALT, AST, GGT
Wong et al,^47^ 2013 Hong Kong	NAFLD	154	46	54	12	Diet and exercise	Minimal intervention	ALT, AST, FLI, liver stiffness, steatosis-MRI
Cheng et al,^31^ 2017 China	NAFLD	86	23	77	9	Arm 1: Diet	Usual care	ALT, AST, GGT, steatosis-MRI
						Arm 2: Exercise^a^		
						Arm 3: Diet and exercise		
Abenavoli et al,^36^ 2017 Italy	NAFLD	30	60	40	6	Arm 1: Diet and exercise	Usual care	ALT, AST, GGT, FLI, liver stiffness, steatosis-US
						Arm 2: Diet, exercise, and antioxidant supplement^a^		
Axley et al,^39^ 2018 United States	NAFLD	30	37	63	6	Diet and exercise	Minimal intervention	ALT, AST
Eckard et al,^40^ 2013 United States	NAFLD	3241	59		6	Arm 1: Low-fat diet and exercise	Minimal intervention	ALT, AST, NAS, fibrosis
						Arm 2: Moderate-fat diet and exercise		
Promrat et al,^45^ 2010 United States	NASH	31	71	29	6	Diet and exercise	Minimal intervention	ALT, AST, NAS, steatosis-H, inflammation, ballooning, fibrosis, definite NASH
Katsagoni et al,^42^ 2018 Greece	NAFLD	63	68	32	6	Arm 1: Diet	Minimal intervention	ALT, GGT, NFS, liver stiffness
						Arm 2: Diet and exercise		
Abd El-Kader et al,^35^ 2016 Saudi Arabia	NASH	100	70	30	3	Diet and exercise	Usual care	ALT, AST
Al-Jiffri et al,^37^ 2013 Saudi Arabia	NAFLD	100	100	0	3	Diet and exercise	Usual care	ALT, AST, ALP, GGT
Asghari et al,^29^ 2018 Iran	NAFLD	60	68	32	3	Arm 1: Diet	Placebo	ALT, AST, steatosis-US,
						Arm 2: Resveratrol^a^		
St George et al,^46^ 2009 Australia	NAFLD^b^	152	63	37	3	Arm 1: Low-intensity diet and exercise	Minimal intervention	ALT, AST, GGT
						Arm 2: Moderate-intensity diet and exercise		
Lim et al,^27^ 2018 Singapore	NAFLD	86	NR	NR	3	Diet and exercise and mobile app	Diet and exercise	ALT, AST
Selezneva et al,^33^ 2014 Russia	NAFLD	174	NR	NR	1	Diet	Isocaloric diet	ALT, AST
Armstrong et al,^38^ 2016 United Kingdom	NASH	52	59	41	12	Liraglutide	Placebo	ALT, AST, ALP, GGT, ELF, NAS, steatosis-H, inflammation, ballooning, fibrosis, definite NASH
Khoo et al,^43^ 2017 Singapore	NAFLD	30	92	8	6	Liraglutide	Diet and exercise	ALT, AST, liver stiffness
Harrison et al,^41^ 2009 United States	NASH	41	32	68	9	Orlistat and diet	Diet	ALT, AST, ALP, NAS, steatosis-H, inflammation, ballooning
Ye et al,^28^ 2017 NR	NASH	30	NR	NR	6	Orlistat	Placebo	Steatosis-MRI
Zelber-Sagi et al,^48^ 2006 Israel	NAFLD	52	43	57	6	Orlistat, diet, and exercise	Placebo, diet, and exercise	ALT, AST, GGT, steatosis-US, steatosis-H, fibrosis
Bahmanadabi et al,^30^ 2011 Iran	NAFLD	40	20	80	3	Sibutramine hydrochloride and diet	Diet	ALT, AST, steatosis-US
Lee et al,^44^ 2012 Singapore	NASH	18	61	39	6	Gastric balloon surgical procedure, diet, and exercise	Sham procedure, diet, and exercise	ALT, NAS, steatosis-H, inflammation, ballooning, fibrosis

Abbreviations: ALP, alkaline phosphatase; ALT, alanine aminotransferase; AST, aspartate transaminase; ELF, Enhanced Liver Fibrosis; FLI, Fatty Liver Index; GGT, γ-glutamyltransferase; NAFLD, nonalcoholic fatty liver disease; NAS, NAFLD activity score; NASH, nonalcoholic steatohepatitis; NFS, NAFLD fibrosis score; NR, not reported; steatosis-H, histologically assessed steatosis; steatosis-MRI, magnetic resonance imaging–assessed steatosis; steatosis-US, ultrasonography assessed steatosis.

^a^ This study arm was excluded from the analysis because it did not meet the review eligibility criteria.

^b^ Eight participants had hepatitis C and were not receiving antiviral therapy (n = 6 in the intervention; n = 2 in the control). We included the study in the analysis because the authors reported that their results did not change in a sensitivity analysis that excluded these participants. We also ran a sensitivity analysis excluding this study, and the estimates of the meta-analysis did not change.

All but 1 study^28^ reported ALT and weight. Eighteen comparisons reported an insulin resistance index (HOMA-IR, Matsuda index, or QUICKI index). Steatosis was examined by ultrasonography (n = 4),^29,32,36,48^ magnetic resonance imaging (n = 4),^28,31,47^ or histologic examination (n = 5).^38,41,44,45,48^ There were 6 comparisons from 5 studies of NAS,^38,40,41,44,45^ and 2 studies reported histologically defined NASH.^38,45^ Additional blood biomarkers included AST, ALP, GGT, the Enhanced Liver Fibrosis score, the NAFLD fibrosis score, and the Fatty Liver Index. Five comparisons reported liver stiffness by ultrasonography.^36,42,43,47^ Four studies reported inflammation and ballooning,^38,41,44,45^ and 6 comparisons reported on liver fibrosis by histologic examination.^38,40,41,44,45^

**Figure 1.**
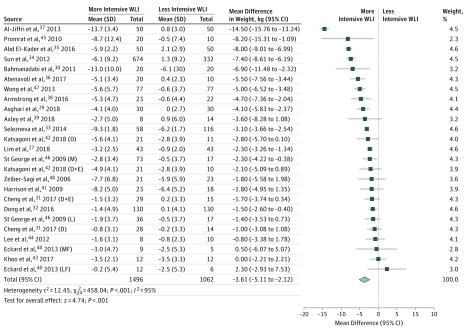
Association Between Weight Loss Intervention (WLI) and Weight Loss (WL) D indicates diet group; D+E, diet and exercise group; L, low-intensity intervention group; LF, low-fat diet group; M, moderate-intensity intervention group; and MF, moderate-fat diet group.

Compared with no or minimal or lower-intensity interventions, more-intensive weight loss interventions, based on evidence, were associated with greater weight change (–3.61 kg; 95% CI, –5.11 to –2.12; *I^2^* = 95%) (Figure 1). Mixed evidence emerged that more-intensive interventions were associated with improved glucose regulation and insulin resistance, because several estimates were imprecise and CIs for insulin and insulin resistance included the null (eFigures 1.1-1.4 in the Supplement).

**Figure 2.**
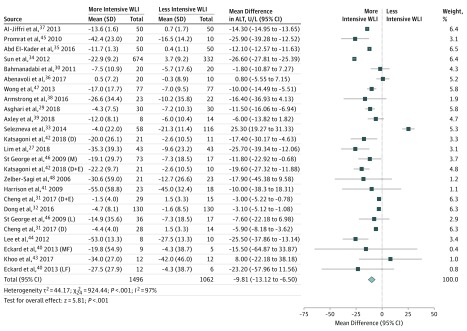
Association Between Weight Loss Intervention (WLI) and Alanine Aminotransferase (ALT) D indicates diet group; D+E, diet and exercise group; L, low-intensity intervention group; LF, low-fat diet group; M, moderate-intensity intervention group; and MF, moderate-fat diet group.

Although the effect size was imprecisely estimated, clear evidence showed that blood markers for liver disease improved, including ALT (–9.81 U/L; 95% CI, –13.12 to –6.50; *I^2^* = 97%; to convert to microkatal per liter, multiply by 0.0167) (Figure 2) and AST (–4.84 U/L; 95% CI, –7.31 to –2.38; *I^2^* = 96%; to convert to microkatal per liter, multiply by 0.0167) (eFigure 1.5 in the Supplement). The change in ALP was –5.53 U/L (95% CI, –20.48 to 9.22; *I^2^* = 96%; to convert to microkatal per liter, multiply by 0.0167) (eFigure 1.6 in the Supplement) and in GGT was –4.35 U/L (95% CI, –7.67 to –1.04; *I^2^* = 92%; to convert to microkatal per liter, multiply by 0.0167) (eFigure 1.7 in the Supplement). No evidence was found of an association between NAFLD fibrosis score (0.15; 95% CI, –0.13 to 0.43; *I^2^* = 68%) (eFigure 1.8 in the Supplement) and Fatty Liver Index (–1.84; 95% CI, –5.08 to 1.40; *I^2^* = 96%) (eFigure 1.9 in the Supplement). Only one trial reported changes in the Enhanced Liver Fibrosis score (–0.40; 95% CI, –0.87 to 0.07) (eFigure 1.10 in the Supplement).

**Figure 3.**
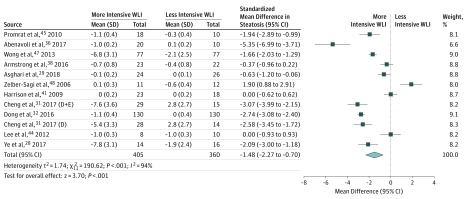
Association Between Weight Loss Intervention (WLI) and Liver Steatosis Standardized mean difference was assessed by histologic examination, magnetic resonance imaging, or ultrasonography. D indicates diet group; D+E, diet and exercise group.

**Figure 4.**
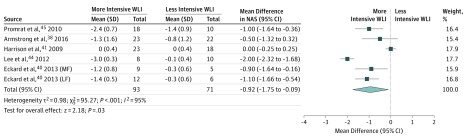
Association Between Weight Loss Intervention (WLI) and NAS (Nonalcoholic Fatty Liver Disease Activity Score) The NAS range is 0-8, with the highest score indicating more severe disease. LF indicates low-fat diet group; MF, moderate-fat diet group.

Clear evidence showed that weight loss interventions were associated with improved liver steatosis measured by histologic examination, magnetic oukidisresonance imaging, or ultrasonography (standardized mean difference, –1.48; 95% CI, –2.27 to –0.70; *I^2^* = 94%) (Figure 3). Clear but imprecise evidence indicated that weight loss interventions were associated with changes in liver stiffness (–1.11 kPa; 95% CI, –1.91 to –0.32; *I^2^* = 94%) (eFigure 1.11 in the Supplement), the NAS score (–0.92; 95% CI, –1.75 to –0.09; *I^2^* = 95%) (Figure 4), and the presence of definite NASH (OR, 0.14; 95% CI, 0.04-0.49; *I^2^* = 0%) (eFigure 1.12 in the Supplement). No evidence was found of changes in the histologic scores for inflammation (–0.01; 95% CI, –0.10 to 0.07; *I^2^* = 0%) (eFigure 1.13 in the Supplement), ballooning (–0.11; 95% CI, –0.26 to 0.04; *I^2^* = 43%) (eFigure 1.14 in the Supplement), or fibrosis (–0.13; 95% CI, –0.54 to 0.27; *I^2^* = 68%) (eFigure 1.15 in the Supplement).

Only 2 trials followed up participants after the end of the intervention: 1 after 6 months,^49^ and 1 after 5 years.^50^ No evidence was found of between-group differences in weight, glucose, HOMA-IR, ALT, or Fatty Liver Index in the long-term follow-up (eFigures 2.1-2.5 in the Supplement).

eTable 3 in the Supplement shows the reported adverse events. Half of the studies (n = 11) did not report on adverse events. Gastrointestinal symptoms were the most commonly reported adverse events in pharmacotherapy and surgical trials. Seven BWLP trials that reported on related adverse events found none.

eFigure 3.1 in the Supplement presents a summary of the risk-of-bias assessments. Three studies were judged to be at low risk of bias across all domains,^38,45,47^ 12 at high risk of bias in at least 1 domain, and the remainder had unclear risk of bias in at least 1 domain. On examination of funnel plots for ALT and AST (eFigure 3.2 in the Supplement), we observed no evidence of publication bias.

We confined the analyses to include only the studies at low risk of bias. The estimates and precision of measures of insulin, ALP, steatosis, NAS, presence of definite NASH, inflammation, fibrosis, and liver stiffness did not materially change. Mean weight change, ALT, and GGT were somewhat larger. Although the statistical significance of the estimates for HOMA-IR, ballooning, glucose, hemoglobin A_1c_, and AST changed, the direction of the association remained the same (eFigures 4.1-4.15 in the Supplement).

In a subgroup analysis by type of program (BWLP or pharmacotherapy), we observed no subgroup differences in possible mediators of the association, namely weight and markers of glucose regulation (eFigures 5.1-5.4 in the Supplement). No evidence was found of subgroup differences in ALT or AST. Steatosis improved with the BWLPs, but no evidence of improvement in steatosis was found with pharmacotherapy alone, but strong evidence of a subgroup difference (eFigures 5.5-5.7 in the Supplement) was noted.

Comparisons of intervention against usual care showed larger effects than the comparisons of higher- with lower-intensity interventions, especially for steatosis in which clear evidence of a subgroup difference was observed (eFigures 6.1-6.7 in the Supplement). In a post hoc analysis, the changes in weight, ALT, and AST were larger in studies that included only participants who were overweight than in studies that included participants irrespective of weight status with strong evidence of subgroup differences. However, no evidence of subgroup differences was found in glucose regulation, insulin resistance, and steatosis (eFigures 7.1-7.7 in the Supplement). Evidence did not show that changes in histologically assessed fibrosis differed between people with all stages of NAFLD and those with NASH, and no evidence was noted of worsening of fibrosis in people with NASH (eFigure 8.1 in the Supplement).

## Discussion

In people with NAFLD, interventions aimed at weight loss were associated with statistically and clinically significant improvements in blood biomarkers of liver disease, such as ALT and AST, as well as radiologic and histologic markers, such as liver stiffness, steatosis, and the NAS. However, no evidence was found that these interventions were associated with changes in histologic liver fibrosis after 6 months. Twelve studies were judged at high risk of bias and 7 studies at unclear risk of bias in at least 1 domain, but sensitivity analyses showed estimates were largely unaffected by excluding these studies.

### Strengths and Limitations 

We followed the Cochrane methods to minimize bias. We included only RCTs to eliminate selection bias and minimize confounding. We sought to minimize outcome reporting bias by imputing incompletely reported negative findings. No perfect measure of liver disease exists, as even liver biopsy, the benchmark, has limitations^51^; thus, we sought evidence of the association of weight loss with a range of outcomes. All but 1 study measured ALT, with fewer studies reporting other biomarkers and only a minority reporting histologic outcomes, limiting conclusions on these outcomes. The steatosis results may have been affected by the different methods of assessing this outcome, but there is no basis for assuming the different methods would bias the findings by trial arm. Most studies were of short- to medium-term duration (median, 6 months), so the long-term association of these interventions with the liver is unclear. This present review included a trial of sibutramine, which is no longer licensed, but removing this trial from the analysis did not materially affect the results. Only 1 trial of bariatric surgery was available, which meant that the evidence on this treatment modality is limited.

The results are limited by the high statistical heterogeneity. As we hypothesized weight loss to be the main driver of change in liver markers, we combined BWLP, pharmacotherapy, and surgical procedure and compared them with either no or minimal intervention or a lower-intensity weight loss intervention. Furthermore, the pooled BWLPs varied in intensity and delivery format, which may explain the marked differences in weight loss achieved within and between these groups as well as the high statistical heterogeneity of the pooled effect size estimates for biomarkers of liver disease. We decided a priori to exclude exercise and diet interventions that did not restrict energy intake, because these interventions were testing a different mechanism of action. The association between changing dietary composition with or without additional physical activity has little implication for weight loss.^52,53,54^ Physical activity may influence biomarkers of liver disease irrespective of weight loss,^55^ but isoenergetic changes in dietary composition have not been shown differential effects on liver markers.^56^

This systematic review synthesized RCTs of weight loss interventions of variable type, content, and duration and quantified their association with liver biomarkers. It included 22 trials and 26 comparisons with 2588 participants from 12 countries, including both high- and middle-income countries. The review updated the 2011 Cochrane systematic review by adding 15 more trials and including a meta-analysis, yielding much stronger evidence of advantage. In contrast with the Cochrane systematic review, we included as a comparison not only no or minimal interventions but also lower-intensity interventions, which allowed a more comprehensive assessment of the research question. In the United Kingdom, the National Institute for Health and Care Excellence review on the assessment and management of NAFLD did not identify any studies of dietary interventions for weight loss in NAFLD but did identify 4 interventions of combined diet, exercise, and behavioral support.^12^

### Implications for Clinicians and Policymakers

The 2018 Practice Guidance of the American Association for the Study of Liver Diseases advises that weight loss generally reduces steatosis. However, in common with European guidelines, the Practice Guidance offers no specific recommendation to refer to or provide formal weight loss programs for treating NAFLD.^10,11,12,13^ The current practice among hepatologists is to advise weight loss for patients with NAFLD or NASH,^57^ often setting targets for 5% or 10% weight loss, but referral to treatment programs is uncommon.^15^ Patients offered access to typical weight loss programs in routine care can expect to lose more than 4 kilograms in 1 year,^58^ whereas advice to lose weight from a clinician is typically followed by only about 1 kg weight loss.^59,60^ Thus, the mean weight loss difference observed in this review approximates that which we might expect if patients were offered treatment in 1 of the weight loss programs typically available in routine clinical care. Accordingly, we would expect to see similar improvements in liver biomarkers and histologic results. The advantages seem to be greater in people who are overweight and with NAFLD, but our exploratory results suggest that weight loss interventions might still be beneficial in the minority of people with healthy weight and NAFLD. Clinicians may use these findings to counsel people with NAFLD on the expected clinically significant improvements in liver biomarkers after weight loss and direct the patients toward valuable interventions.

Most trials included people at various stages of NAFLD, and only 6 studies specifically included people with a NASH diagnosis. The Food and Drug Administration considers licensing pharmacotherapy for NASH if trials show the resolution of NASH without worsening fibrosis, and these weight loss interventions seem to have met this criterion.^61^ Because BWLPs have cardiometabolic advantages, which make them cost-effective, referral to these programs is likely to be particularly valuable for this population at high risk for cardiovascular disease.^58,62^

### Unanswered Questions and Future Research

Most RCTs followed up participants for 1 year or less, and only 1 trial reported longer-term follow-up, which showed a mean weight difference between groups of –2.30 kg (95% CI, –3.71 to –0.89) 5 years after the end of the intervention, but no evidence was found of between-group differences in liver biomarkers, which were imprecisely estimated.^50^ We did not include non–RCTs, but an uncontrolled study of a BWLP in NASH with paired biopsies at 1 year found a strong and independent association between weight loss and improvements in liver histologic examination, highlighting the need for future RCTs of programs that can help patients maintain substantial weight loss. Weight regain is common after program completion,^58,63^ and future trials should include long-term follow-up to examine the association between weight regain and biomarkers of liver disease or long-term cardiovascular outcomes. We included only trials in adults, but we recognize that with the increasing prevalence of obesity at younger ages, NAFLD is an emerging condition in childhood and warrants future consideration.^64,65^ Future trials might also incorporate subgroup analysis by BMI status, examining the advantages in people with NAFD and healthy weight.

## Conclusions

Weight loss interventions appeared to be associated with statistically and clinically significant improvements in biomarkers of liver disease in people with NAFLD in the short term. The accumulated evidence supports changing the clinical guidelines and routine practice to recommend formal weight loss programs to treat people with NAFLD.

## Notes

Supplement. **eTable 1.** Characteristics of the Interventions of the Included Studies **eTable 2.** Handing of Missing Data in Each Study **eTable 3.** Reported Adverse Events **eFigure 1.** PRISMA Flowchart **eFigure 1.1** Glucose (mmol/L) **eFigure 1.2** HbA1c (%) **eFigure 1.3** Insulin Resistance Index (HOMA-IR, QUICKI, or Matsuda) **eFigure 1.4** Insulin (pmol/L) **eFigure 1.5** Aspartate Transaminase – AST (U/L) **eFigure 1.6** Alkaline Phosphatase – ALP (U/L) **eFigure 1.7** γ-Glutamyl Transferase – GGT (U/L) **eFigure 1.8** NAFLD Fibrosis Score **eFigure 1.9** Fatty Liver Index **eFigure 1.10** Enhanced Liver Fibrosis (ELF) score **eFigure 1.11** Liver Stiffness (kPa) **eFigure 1.12** Presence of Definite NASH (Yes/No) **eFigure 1.13** Inflammation (Score 0-3) **eFigure 1.14** Ballooning (Score 0-2) **eFigure 1.15** Fibrosis (Stage F0-F4) **eFigure 2.1** Weight (kg) (Long-term) **eFigure 2.2** Glucose (mmol/L) (Long-term) **eFigure 2.3** HOMA-IR (Long-term) **eFigure 2.4** Alanine Aminotransferase – ALT (U/L) (Long-term) **eFigure 2.5** Fatty Liver Index (Long-term) **eFigure 3.1** Risk of Bias Within Studies **eFigure 3.2** Funnel Plot of Studies Reporting (a) Alanine Aminotransferase (ALT) and (b) Aspartate Transaminase (AST) **eFigure 4.1** Weight (kg) (Sensitivity Analysis) **eFigure 4.2** HOMA-IR (Sensitivity Analysis) **eFigure 4.3** Glucose (mmol/L) (Sensitivity Analysis) **eFigure 4.4** Insulin (pmol/L) (Sensitivity Analysis) **eFigure 4.5** HbA1c (%) (Sensitivity Analysis) **eFigure 4.6** Alanine Aminotransferase – ALT (U/L) (Sensitivity Analysis) **eFigure 4.7** Liver Stiffness (kPa) (Sensitivity Analysis) **eFigure 4.8** Steatosis – Standardized Mean Difference as Assessed by Histologic Examination or MRI (Sensitivity Analysis) **eFigure 4.9** NAFLD Activity Score (NAS) (Sensitivity Analysis) **eFigure 4.10** Inflammation (score 0-3) (Sensitivity Analysis) **eFigure 4.11** Ballooning (score 0-2) (Sensitivity Analysis) **eFigure 4.12** Fibrosis (scale F0-F4) (Sensitivity Analysis) **eFigure 4.13** Aspartate transaminase – AST (U/L) (Sensitivity Analysis) **eFigure 4.14** Alkaline phosphatase – ALP (U/L) (Sensitivity Analysis) **eFigure 4.15** γ-Glutamyl Transferase – GGT (U/L) (Sensitivity Analysis) **eFigure 5.1** Weight (kg) (Subgroup Analysis by the Type of Intervention) **eFigure 5.2** Glucose (mmol/L) (Subgroup Analysis by the Type of Intervention) **eFigure 5.3** Insulin Resistance Index (HOMA-IR or other) (Subgroup Analysis by the Type of Intervention) **eFigure 5.4** Insulin (pmol/L) (Subgroup Analysis by the Type of Intervention) **eFigure 5.5** ALT (U/L) (Subgroup Analysis by the Type of Intervention) **eFigure 5.6** Steatosis – Standardized Mean Difference as Assessed by Histologic Examination or MRI (Subgroup Analysis by the Type of Intervention) **eFigure 5.7** AST (U/L) (Subgroup Analysis by the Type of Intervention) **eFigure 6.1** Weight Change (kg) by the Intensity of the Comparator Intervention **eFigure 6.2** Glucose (mmol/L) by the Intensity of the Comparator Intervention **eFigure 6.3** Insulin Resistance Indices by the Intensity of the Comparator Intervention **eFigure 6.4** Insulin (pmol/L) by the Intensity of the Comparator Intervention **eFigure 6.5** ALT (U/L) by the Intensity of the Comparator Intervention **eFigure 6.6** AST (U/L) by the Intensity of the Comparator Intervention **eFigure 6.7** Steatosis by the Intensity of the Comparator Intervention **eFigure 7.1** Weight loss by the Presence of a Minimum Overweight/Obesity Cut-off **eFigure 7.2** Glucose (mmol/L) by the Presence of a Minimum Overweight/Obesity Cut-off **eFigure 7.3** Insulin Resistance Index (HOMA-IR or Other) by the Presence of a Minimum Overweight/Obesity Cut-off **eFigure 7.4** Insulin (pmol/L) by the Presence of a Minimum Overweight/Obesity Cut-off **eFigure 7.5** ALT (U/L) by the Presence of a Minimum Overweight/Obesity Cut-off **eFigure 7.6** AST (U/L) by the Presence of a Minimum Overweight/Obesity Cut-off **eFigure 7.7** Steatosis (Standardized Mean Difference as Assessed by Histologic Examination, MRI, or Ultrasonography) by the Presence of a Minimum Overweight/Obesity Cut-off **eFigure 8.1** Fibrosis (Stage F0-F4) **eMethods** Search Strategy
