# Supplementary material for: Association of Weight Loss Interventions With Changes in Biomarkers of Nonalcoholic Fatty Liver Disease: A Systematic Review and Meta-analysis
Source: JAMA Intern Med. 2019 Jul 1;179(9):1262–71. doi: 10.1001/jamainternmed.2019.2248 (PMC6604126; doi:10.1001/jamainternmed.2019.2248)
Supplement: Supplement. — eTable 1. Characteristics of the Interventions of the Included Studies eTable 2. Handing of Missing Data in Each Study eTable 3. Reported Adverse Events eFigure 1. PRISMA Flowchart eFigure 1.1 Glucose (mmol/L) eFigure 1.2 HbA1c (%) eFigure 1.3 Insulin Resistance Index (HOMA-IR, QUICKI, or Matsuda) eFigure 1.4 Insulin (pmol/L) eFigure 1.5 Aspartate Transaminase – AST (U/L) eFigure 1.6 Alkaline Phosphatase – ALP (U/L) eFigure 1.7 γ-Glutamyl Transferase – GGT (U/L) eFigure 1.8 NAFLD Fibrosis Score eFigure 1.9 Fatty Liver Index eFigure 1.10 Enhanced Liver Fibrosis (ELF) score eFigure 1.11 Liver Stiffness (kPa) eFigure 1.12 Presence of Definite NASH (Yes/No) eFigure 1.13 Inflammation (Score 0-3) eFigure 1.14 Ballooning (Score 0-2) eFigure 1.15 Fibrosis (Stage F0-F4) eFigure 2.1 Weight (kg) (Long-term) eFigure 2.2 Glucose (mmol/L) (Long-term) eFigure 2.3 HOMA-IR (Long-term) eFigure 2.4 Alanine Aminotransferase – ALT (U/L) (Long-term) eFigure 2.5 Fatty Liver Index (Long-term) eFigure 3.1 Risk of Bias Within Studies eFigure 3.2 Funnel Plot of Studies Reporting (a) Alanine Aminotransferase (ALT) and (b) Aspartate Transaminase (AST) eFigure 4.1 Weight (kg) (Sensitivity Analysis) eFigure 4.2 HOMA-IR (Sensitivity Analysis) eFigure 4.3 Glucose (mmol/L) (Sensitivity Analysis) eFigure 4.4 Insulin (pmol/L) (Sensitivity Analysis) eFigure 4.5 HbA1c (%) (Sensitivity Analysis) eFigure 4.6 Alanine Aminotransferase – ALT (U/L) (Sensitivity Analysis) eFigure 4.7 Liver Stiffness (kPa) (Sensitivity Analysis) eFigure 4.8 Steatosis – Standardized Mean Difference as Assessed by Histologic Examination or MRI (Sensitivity Analysis) eFigure 4.9 NAFLD Activity Score (NAS) (Sensitivity Analysis) eFigure 4.10 Inflammation (score 0-3) (Sensitivity Analysis) eFigure 4.11 Ballooning (score 0-2) (Sensitivity Analysis) eFigure 4.12 Fibrosis (scale F0-F4) (Sensitivity Analysis) eFigure 4.13 Aspartate transaminase – AST (U/L) (Sensitivity Analysis) eFigure 4.14 Alkaline phosphatase – ALP (U/L) (Sensitivity [file jamainternmed-179-1262-s001.pdf]

Supplementary Online Content

Koutoukidis DA, Astbury NM, Tudor KE, et al. Association of weight loss interventions with changes in biomarkers of nonalcoholic fatty liver disease: a systematic review and meta-analysis. *JAMA Intern Med*. Published online July 1, 2019. doi:10.1001/jamainternmed.2019.2248

eFigure 1 PRISMA Flowchart
eTable 1 Characteristics of the Interventions of the Included Studies
eTable 2 Handing of Missing Data in Each Study
eTable 3 Reported Adverse Events
eFigure 1.1 Glucose (mmol/L)
eFigure 1.2 HbA1c (%)
eFigure 1.3 Insulin Resistance Index (HOMA-IR, QUICKI, or Matsuda)
eFigure 1.4 Insulin (pmol/L)
eFigure 1.5 Aspartate Transaminase – AST (U/L)
eFigure 1.6 Alkaline Phosphatase – ALP (U/L)
eFigure 1.7 γ-Glutamyl Transferase – GGT (U/L)
eFigure 1.8 NAFLD Fibrosis Score
eFigure 1.9 Fatty Liver Index
eFigure 1.10 Enhanced Liver Fibrosis (ELF) score
eFigure 1.11 Liver Stiffness (kPa)
eFigure 1.12 Presence of Definite NASH (Yes/No)
eFigure 1.13 Inflammation (Score 0-3)
eFigure 1.14 Ballooning (Score 0-2)
eFigure 1.15 Fibrosis (Stage F0-F4)
eFigure 2.1 Weight (kg) (Long-term)
eFigure 2.2 Glucose (mmol/L) (Long-term)
eFigure 2.3 HOMA-IR (Long-term)
eFigure 2.4 Alanine Aminotransferase – ALT (U/L) (Long-term)
eFigure 2.5 Fatty Liver Index (Long-term)
eFigure 3.1 Risk of Bias Within Studies
eFigure 3.2 Funnel Plot of Studies Reporting (a) Alanine Aminotransferase (ALT) and (b) Aspartate Transaminase (AST)
eFigure 4.1 Weight (kg) (Sensitivity Analysis)
eFigure 4.2 HOMA-IR (Sensitivity Analysis)
eFigure 4.3 Glucose (mmol/L) (Sensitivity Analysis)
eFigure 4.4 Insulin (pmol/L) (Sensitivity Analysis)
eFigure 4.5 HbA1c (%) (Sensitivity Analysis)
eFigure 4.6 Alanine Aminotransferase – ALT (U/L) (Sensitivity Analysis)
eFigure 4.7 Liver Stiffness (kPa) (Sensitivity Analysis)
eFigure 4.8 Steatosis – Standardized Mean Difference as Assessed by Histologic Examination or MRI (Sensitivity Analysis)
eFigure 4.9 NAFLD Activity Score (NAS) (Sensitivity Analysis)
eFigure 4.10 Inflammation (score 0-3) (Sensitivity Analysis)
eFigure 4.11 Ballooning (score 0-2) (Sensitivity Analysis)

© 2019 Koutoukidis DA et al. *JAMA Internal Medicine*.

eFigure 4.12 Fibrosis (scale F0-F4) (Sensitivity Analysis)
eFigure 4.13 Aspartate transaminase – AST (U/L) (Sensitivity Analysis)
eFigure 4.14 Alkaline phosphatase – ALP (U/L) (Sensitivity Analysis)
eFigure 4.15 γ-Glutamyl Transferase – GGT (U/L) (Sensitivity Analysis)
eFigure 5.1 Weight (kg) (Subgroup Analysis by the Type of Intervention)
eFigure 5.2 Glucose (mmol/L) (Subgroup Analysis by the Type of Intervention)
eFigure 5.3 Insulin Resistance Index (HOMA-IR or other) (Subgroup Analysis by the Type of Intervention)
eFigure 5.4 Insulin (pmol/L) (Subgroup Analysis by the Type of Intervention)
eFigure 5.5 ALT (U/L) (Subgroup Analysis by the Type of Intervention)
eFigure 5.6 Steatosis – Standardized Mean Difference as Assessed by Histologic Examination or MRI (Subgroup Analysis by the Type of Intervention)
eFigure 5.7 AST (U/L) (Subgroup Analysis by the Type of Intervention)
eFigure 6.1 Weight Change (kg) by the Intensity of the Comparator Intervention
eFigure 6.2 Glucose (mmol/L) by the Intensity of the Comparator Intervention
eFigure 6.3 Insulin Resistance Indices by the Intensity of the Comparator Intervention
eFigure 6.4 Insulin (pmol/L) by the Intensity of the Comparator Intervention
eFigure 6.5 ALT (U/L) by the Intensity of the Comparator Intervention
eFigure 6.6 AST (U/L) by the Intensity of the Comparator Intervention
eFigure 6.7 Steatosis by the Intensity of the Comparator Intervention
eFigure 7.1 Weight loss by the Presence of a Minimum Overweight/Obesity Cut-off
eFigure 7.2 Glucose (mmol/L) by the Presence of a Minimum Overweight/Obesity Cut-off
eFigure 7.3 Insulin Resistance Index (HOMA-IR or Other) by the Presence of a Minimum Overweight/Obesity Cut-off
eFigure 7.4 Insulin (pmol/L) by the Presence of a Minimum Overweight/Obesity Cut-off
eFigure 7.5 ALT (U/L) by the Presence of a Minimum Overweight/Obesity Cut-off
eFigure 7.6 AST (U/L) by the Presence of a Minimum Overweight/Obesity Cut-off
eFigure 7.7 Steatosis (Standardized Mean Difference as Assessed by Histologic Examination, MRI, or Ultrasonography) by the Presence of a Minimum Overweight/Obesity Cut-off
eFigure 8.1 Fibrosis (Stage F0-F4)
eMethods Search Strategy

This supplementary material has been provided by the authors to give readers additional information about their work.

© 2019 Koutoukidis DA et al. *JAMA Internal Medicine*.

eFigure 1 PRISMA flowchart

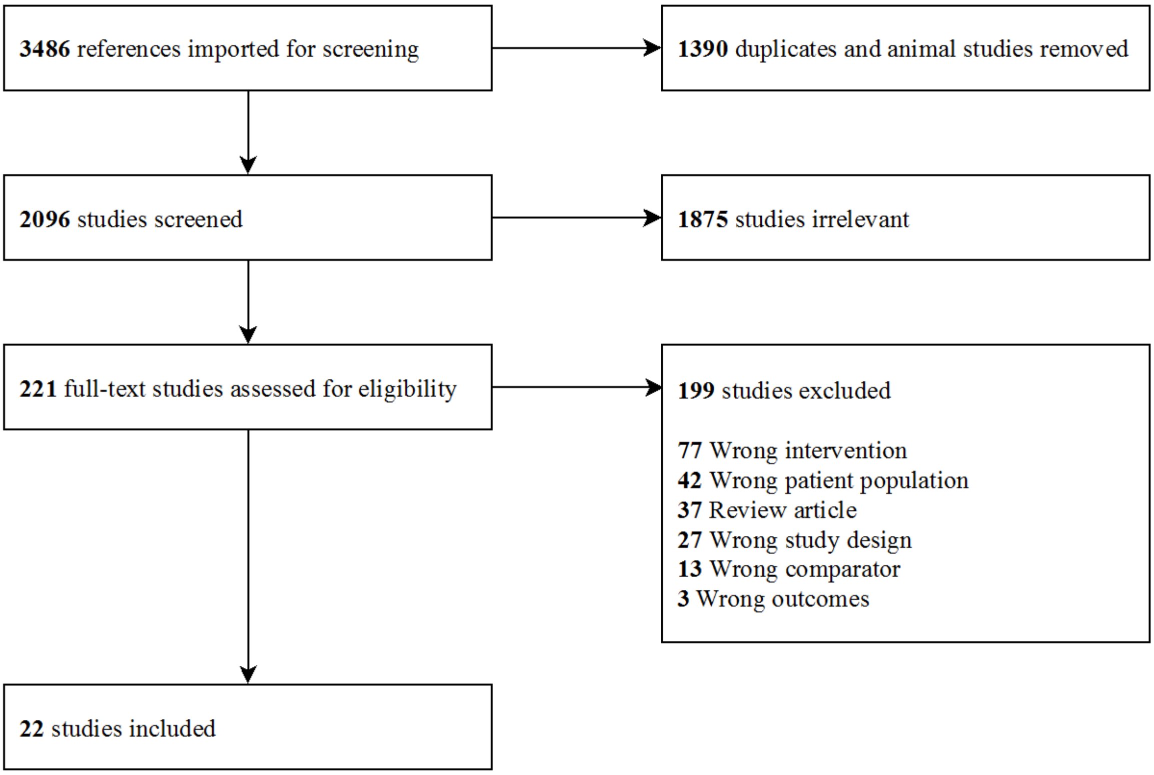

© 2019 Koutoukidis DA et al. *JAMA Internal Medicine*.

eTable 1 Characteristics of the interventions of the included studies

Study and country	Intervention	Provider	Delivery and Intensity	Duration	Comparison
Dong, 2016, China	Diet: 25-30kcal/kg/d if at least overweight, avoid greasy food<br><br>Exercise: advice for aerobic 3-4x 30-60min/wk	Dietitian and exercise physiologist	Phone, 10minutes thi-monthly	2 years	Usual care
Sun, 2012, China	Diet: 25kcal/kg/day, low-fat, low-sugar, optimized mixed diet<br><br>Exercise: advice to increase PA (23METs/h/wk) and exercise therapy (4METs/h/wk)	Physicians, hygienists, dietitians and nurses	Unclear	1 year	Basic healthy eating/exercise/weight loss advice
Wong, 2013, Hong Kong	Diet: hypoenergetic, low-fat, low-GI, varied, meal plans, and booklets<br><br>Exercise:<br>advice for 3-5x 30min/wk	Dietitian and exercise instructor	Face-to-face, individual, weekly for months 0-4 and monthly for months 5-12	1 year	Basic healthy eating/exercise/weight loss advice (Advice to reduce carbohydrate and fat intake and exercise 3x 30min/wk)
Cheng, 2017, China	Diet: 300-500kcal deficit/day if at least with overweight, low-carbohydrate with prepared meals<br><br>Exercise: aerobic 2-3 times/wk, progressively increased	Nutritionist and exercise researcher	Face-to-face, 3x nutrition meetings, 2-3 exercise classes/week	8.6 months	Usual care: Maintain ordinary lifestyle
Abenavoli, 2017, Italy	Diet: 1400-1600kcal/d, Mediterranean<br><br>Exercise: advice for daily physical activity	Unclear	Telephone, monthly	6 months	Usual care: Only monitored with the medical indication for weight loss
Axley, 2017, USA	Diet and exercise	Automated	Text messaging. 6x weekly	6 months	Basic healthy lifestyle/weight loss instructions
Eckard, 2013, USA	Arm 1: Diet: low-fat<br>Arm 2: Diet: moderate-fat and low-processed-carbohydrate	Dietitian and exercise physiologist	Face-to-face, bi-monthly (diet)	6 months	Basic healthy eating/exercise/weight loss advice

© 2019 Koutoukidis DA et al. *JAMA Internal Medicine*.

	Diet (both arms): 500kcal deficit/day, received measuring cups and spoons and booklets<br><br>Exercise (both arms): 1 class, log, pedometer and advice for -4-7x 20-60min/wk				
Promrat, 2010, USA	Diet: 1000-1500kcal/day, low-fat, meal replacements, meal plan, based on DPP<br><br>Exercise: pedometer, log, advice for ≥200min/wk or 10,000steps/day	Nutritionist or health educator	Face to face, group, weekly for months 0-6 and biweekly for months 7-12	1 year (48 wk's)	Basic healthy lifestyle/weight loss instructions
Katsagoni, 2018, Greece	Diet: 1500-1800kcal/day, Mediterranean<br><br>Exercise (for exercise group only): advice for 30min/day (also: advice for optimal sleep duration, and mid-day rest)	Dietitian	Face-to-face, group, fortnightly for months 0-2 and monthly for months 3-6	6 months	Minimal intervention
AbdEl-Kader, 2016, Saudi Arabia	Diet: 1200kcal/d, balanced<br><br>Exercise: aerobic 3x 30min/wk on treadmill	Dietitians	Unclear	3 months	Usual care: Maintain ordinary lifestyle
Al-Jiffri, 2013, Saudi Arabia	Diet: 1200kcal/d, balanced<br><br>Exercise: aerobic 3x30min/wk on treadmill	Dietitians	Unclear	Diet: 2 months<br>Exercise: 3 months	Usual care: No intervention
Asghari, 2018, Iran	Diet: 500-1000kcal deficit/day, healthy	Nutritionist	Face to face, biweekly	3 months	Placebo<br>Basic healthy lifestyle/weight loss instructions
St. George, 2009, Australia	Diet: 400-570kcal deficit/day, low in saturated fat and processed food, high in n-3 and fiber<br><br>Exercise: advice for (mainly walking) ≥200min/wk	Dietitians and exercise physiologists	Face-to-face, individual,<br><br>Low-intensity arm: 3x fortnightly	3 months	Basic healthy lifestyle/weight loss instructions

© 2019 Koutoukidis DA et al. *JAMA Internal Medicine*.

			Moderate-intensity arm: 6x fortnightly		
Lim, 2018, Singapore	Diet and exercise counselling for weight loss plus mobile app	Mobile app (unclear for counselling	Unclear	Unclear	Diet and exercise counselling
Selezneva, 2014, Russia	Diet: 1600-1700kcal/day	Unclear	Unclear	1 month	Isocaloric diet: 2500-2700kcal/day
Armstrong, 2016, UK	Liraglutide: 1.8mg<br><br>Standard recommendations for lifestyle modification	N/A	Subcutaneous injections, daily	12 months (48 wk's)	Placebo<br><br>Standard recommendations for lifestyle modification
Khoo, 2017, Singapore	Liraglutide: 0.6mg and progressively increased to 3mg by 4th week	N/A	Subcutaneous injections, daily	6 months	Diet: 400kcal deficit/day<br><br>Exercise: advice for moderate-intensity 5-7x 30-60min/wk
Harrison, 2007, USA	Orlistat: 120mg<br><br>Diet: 1400kcal/day<br><br>Exercise: non-specific advice to increase PA	Research nurse and physician	Orlistat: 3x daily<br><br>Diet: Face-to-face	Orlistat: 9 months (36 wk's) Diet: 1 month	Diet (as per the intervention group)
Ye, 2017, NR	Orlistat: 120mg	N/A	3x daily	6 months	Placebo
Zelber-Sagi, 2004, Israel	Orlistat: 120mg<br><br>Diet: 25kcal/kg/day, low-fat, low in simple carbohydrates, balanced<br><br>Exercise: advice for walking 3-4x 40min/wk	Nutritionist	Orlistat: 3x daily<br><br>Diet: Face-to-face, individual, monthly	6 months	Placebo<br><br>Diet and exercise (as per the intervention group)

© 2019 Koutoukidis DA et al. *JAMA Internal Medicine*.

Bahmanadabi, 2011, Iran	Sibutramine: 15mg<br><br>Diet: low-calorie	Unclear	Sibutramine: daily<br><br>Diet: Unclear	3 months	Diet: low-calorie
Lee, 2012, Singapore	Gastric balloon<br><br>Diet: 1200-1500kcal/day<br><br>Exercise: advice for walking/jogging 30min/day	Dietitian and physiotherapist	Monthly	6 months	Sham surgery<br><br>Diet and exercise (as per the intervention group)
PA: physical activity, wk: week					

© 2019 Koutoukidis DA et al. *JAMA Internal Medicine*.

eTable 2 Additional study characteristics

Study and country	Age (years)	BMI (kg/m^2)	Type 2 Diabetes	Hypertension
Dong, 2016, China	57 ± 6	25.8 ± 2.7	0%	NR
Sun, 2012, China	39 ± 15	37.9 ± 14.3	6%	NR
Wong, 2013, Hong Kong	51 ± 9	25.4 ± 3.6	8%	29%
Cheng, 2017, China	60 ± 4	26.7 ± 2.8	0%	NR
Abenavoli, 2017, Italy	45 ± 13	30.3 ± 3.1	0%	0%
Axley, 2017, USA	53 ± 9	37.3 ± 8.2	33%	NR
Eckard, 2013, USA	50 ± 10	35.7 ± 6.6	19%	56%
Promrat, 2010, USA	48 ± 11	33.6 ± 5	48%	NR
Katsagoni, 2018, Greece	46 ± 14	31.4 ± 4.4	0%	NR
AbdEl-Kader, 2016, Saudi Arabia	51 ± 6	32.1 ± 2.7	NR	NR
Al-Jiffri, 2013, Saudi Arabia	NR	32.2 ± 3.7	100%	0%
Asghari, 2018, Iran	40 ± 6	30.9 ± 3.4	0%	NR
StGeorge, 2009, Australia	48 ± 11	31.7 ± 5.3	19%	66%
Lim, 2018, Singapore	NR	NR	NR	NR
Selezneva, 2014, Russia	41 ± 2	37.1 ± 1.8	NR	NR
Armstrong, 2016, UK	51 ± 11	36 ± 5.7	33%	56%
Khoo, 2017, Singapore	41 ± 9	33.1 ± 3.5	0%	0%
Harrison, 2007, USA	47 ± 9	36.4 ± 6.2	10%	NR
Ye, 2017, NR	NR	28.9 ± 3.8	NR	NR
Zelber-Sagi, 2004, Israel	48 ± 10	33.1 ± 5.6	8%	NR
Bahmanadabi, 2011, Iran	38 ± 7	35.1 ± 7.3	0%	0%
Lee, 2012, Singapore	45 ± 17	31.5 ± 7.6	11%	NR
Data are reported as mean ± SD or proportion of total N. NR: Not reported.				

© 2019 Koutoukidis DA et al. *JAMA Internal Medicine*.

eTable 3 Handing of missing data in each study

Study	Year	Total N randomized	N analyzed	Missing data handling
Dong	2016	280	260	Complete case analysis
Sun	2012	1087	1006	Complete case analysis
Wong	2013	154	154	Missing data - last observation carried forward
Cheng[1]	2017	86	86	Missing data - multiple imputation and reporting of marginal means
Abenavoli[1]	2017	30	30	No missing data
Axley	2017	30	22	Missing data - no information on imputation – assumed complete case analysis
Eckard	2013	32	32	Complete case analysis
Promrat	2008	31	30	Complete case analysis
Katsagoni	2018	63	63	Missing data - multiple imputation
AbdEl-Kader	2016	100	100	No information – assumed 100% follow-up rate
Al-Jiffri	2013	100	100	No missing data
Asghari[1]	2018	60	60	Missing data – imputation (personal communication)
StGeorge	2009	152	143	Complete case analysis
Lim	2018	86	86	Complete case analysis - INTERIM
Selezneva	2014	174	174	No information – assumed 100% follow-up rate
Armstrong	2016	52	45	Complete case analysis
Khoo	2017	30	24	Complete case analysis
Harrison	2007	41	41	Complete case analysis
Ye	2017	30	30	Complete case analysis - INTERIM
Zelber-Sagi	2004	52	44	Missing data – complete case analysis in tables but mentions last observation carried forward in the statistical section
Bahmanabadi	2011	40	40	Unclear followed up rate – manuscript mentions that those with low adherence were excluded but unclear how many these were – assumed 100% follow-up
Lee	2009	18	18	Complete case analysis
[1] Numbers exclude participants in trial arms that were not included in the current review				

© 2019 Koutoukidis DA et al. *JAMA Internal Medicine*.

eTable 4 Reported adverse events

Study and country	Adverse events in the intervention group	Adverse events in the control group
Dong, 2016, China	None	None
Sun, 2012, China	Unclear	Unclear
Wong, 2013, Hong Kong	Unclear	Unclear
Cheng, 2017, China	Unclear	Unclear
Abenavoli, 2017, Italy	None	None
Axley, 2017, USA	None	None
Eckard, 2013, USA	Unclear	Unclear
Promrat, 2010, USA	None	None
Katsagoni, 2018, Greece	None	None
AbdEl-Kader, 2016, Saudi Arabia	Unclear	Unclear
Al-Jiffri, 2013, Saudi Arabia	None	None
Asghari, 2018, Iran	None	None
St. George, 2009, Australia	Unclear	Unclear
Lim, 2018, Singapore	Unclear	Unclear
Selezneva, 2014, Russia	Unclear	Unclear
Armstrong, 2016, UK	% serious AEs: 8%<br>% any AE: 88%<br><br>AEs included: gastrointestinal disorders, nausea, diarrhea, abdominal pain, vomiting, constipation, dyspepsia, flatulence, bloating, eye disorders, cardiac disorders, general disorders and administration site conditions, injection site reaction, fatigue, influenza-like symptoms, peripheral edema, chills, non-specific pain, infections and infestations, investigations, increased aspartate aminotransferase, metabolism and nutrition disorders, anorexia (loss of appetite), musculoskeletal and connective disorders 8, back pain, arthralgia, nervous system disorders, dizziness, headaches or migraines, psychiatric disorders, depression, renal and urinary disorders, respiratory, thoracic, and mediastinal disorders, cough, skin and soft tissue disorders	% serious AEs: 8%<br>% any AE: 92%<br><br>AEs included: gastrointestinal disorders, nausea, diarrhea, abdominal pain, vomiting, dyspepsia, eye disorders, cardiac disorders, general disorders and administration site conditions, injection site reaction, fatigue, influenza-like symptoms, peripheral edema, non-specific pain, infections and infestations, chest infection, urinary tract infection, investigations, increased γ-glutamyl transferase, increased aspartate aminotransferase, metabolism and nutrition disorders, anorexia (loss of appetite), musculoskeletal and connective disorders 8, back pain, arthralgia, muscle cramps, nervous system disorders, dizziness, headaches or migraines, psychiatric disorders, depression, renal and urinary disorders, respiratory, thoracic, and mediastinal disorders, cough, skin and soft tissue disorders
Khoo, 2017, Singapore	AEs included: Nausea, transient abdominal discomfort and bloating, injection site reaction	None
Harrison, 2007, USA	Unclear	Unclear
Ye, 2017, NR	Unclear	Unclear
Zelber-Sagi, 2004, Israel	AEs included: Gastrointestinal disorders	AEs included: Gastrointestinal disorders
Bahmanadabi, 2011, Iran	Unclear	Unclear

© 2019 Koutoukidis DA et al. *JAMA Internal Medicine*.

Lee, 2012, Singapore	AEs included: Epigastric discomfort, vomiting	AEs included: Epigastric discomfort, vomiting

© 2019 Koutoukidis DA et al. *JAMA Internal Medicine*.

1 Additional outcomes for the main analysis

eFigure 1.1 Glucose (mmol/L)

Study or Subgroup	more intensive WL Mean	more intensive WL SD	more intensive WL Total	less intensive WL Mean	less intensive WL SD	less intensive WL Total	Weight	Mean Difference IV, Random, 95% CI	Risk of Bias A	B	C	D	E	F
b. Promrat 2010	−0.6	0.8	20	−1.7	3	10	1.2%	1.10 [−0.79, 2.99]	+	+		+	+	
d. Sun 2012	−1.6	0.7	674	−0.2	1	332	6.9%	−1.40 [−1.52, −1.28]	+	?		+	+	
f. Abenavoli 2017	−0.2	0.2	20	0	0.1	10	6.9%	−0.20 [−0.31, −0.09]	−	?		+	+	
g. Wong 2013	0	0.6	77	0.1	0.6	77	6.7%	−0.10 [−0.29, 0.09]	+	+			+	
h. Armstrong 2016	−1	1.5	23	0.7	2.3	23	2.6%	−1.70 [−2.82, −0.58]	+	+	+	+	+	
i. Asghari 2018	0.2	0.2	30	0.1	0.1	30	7.0%	0.10 [0.02, 0.18]	+	−	+	+	+	
l. Katsagoni 2018 (D)	0	0.2	21	0.1	0.1	11	6.9%	−0.10 [−0.20, 0.00]	+	+		+	−	
n. StGeorge 2009 (M)	−0.2	0.7	73	0	0.7	17	5.9%	−0.20 [−0.57, 0.17]	?	?			+	−
o. Katsagoni 2018 (D+E)	−0.1	0.1	21	0.1	0.1	10	7.0%	−0.20 [−0.28, −0.12]	+	+		+	−	
p. Zelber-Sagi 2004	−7.1	0.9	21	−5.1	0.3	23	5.8%	−2.00 [−2.40, −1.60]	−	−	+	+	+	
q. Harrison 2007	−0.4	0.2	23	−0.7	1.8	18	3.6%	0.30 [−0.54, 1.14]	?	−	−	−	+	−
r. Cheng 2017 (D+E)	0.1	0.2	29	0.2	0.2	15	6.9%	−0.10 [−0.22, 0.02]	+	+		+	+	−
s. Dong 2016	0	0.5	130	0.2	0.5	130	6.9%	−0.20 [−0.32, −0.08]	+	?		+	+	−
t. StGeorge 2009 (L)	−0.3	0.6	36	0	0.7	17	5.9%	−0.30 [−0.69, 0.09]	?	?			+	−
u. Cheng 2017 (D)	0.3	0.2	28	0.2	0.2	14	6.9%	0.10 [−0.03, 0.23]	+	+		+	+	−
w. Eckard 2013 (MF)	−0.1	1.1	9	0.1	0.5	5	3.6%	−0.20 [−1.04, 0.64]	+	?		+	+	
x. Khoo 2017	−0.7	0.6	12	−0.6	0.5	12	5.6%	−0.10 [−0.54, 0.34]	+	+	−	+	+	
y. Eckard 2013 (LF)	−0.5	1.3	12	0.1	0.5	6	3.6%	−0.60 [−1.44, 0.24]	+	?		+	+	
**Total (95% CI)**			**1259**			**760**	**100.0%**	**−0.34 [−0.57, −0.12]**						

Heterogeneity: $Tau^2 = 0.19$; $Chi^2 = 554.22$, df = 17 (P < 0.00001); $I^2 = 97\%$
Test for overall effect: Z = 2.96 (P = 0.003)

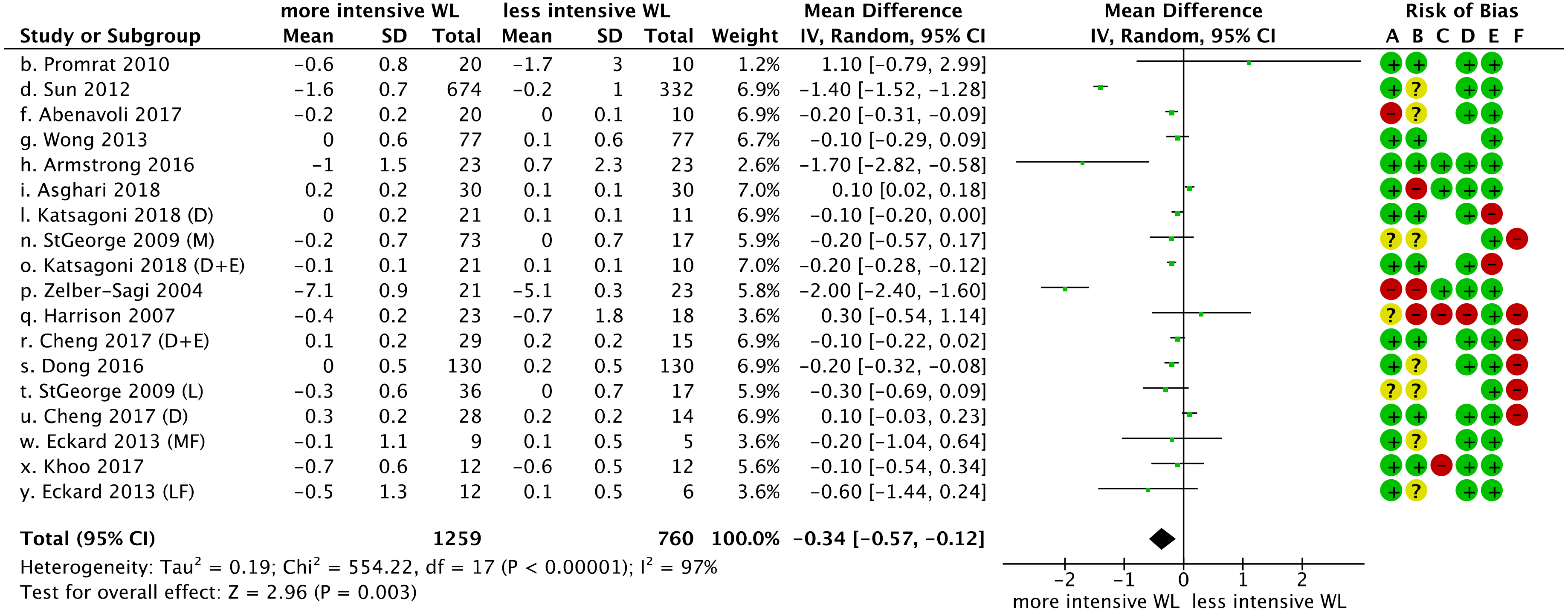

Risk of bias legend for all forest plots

- **(A)** Random sequence generation (selection bias)
- **(B)** Allocation concealment (selection bias)
- **(C)** Blinding of participants and personnel (performance bias)
- **(D)** Blinding of subjective outcome assessment
- **(E)** Incomplete outcome data (attrition bias)
- **(F)** Other bias

© 2019 Koutoukidis DA et al. *JAMA Internal Medicine*.

eFigure 1.2 HbA1c (%)

Study or Subgroup	more intensive WL Mean	SD	Total	less intensive WL Mean	SD	Total	Weight	Mean Difference IV, Random, 95% CI	Risk of Bias A	B	C	D	E	F
b. Promrat 2010	−0.5	0.5	20	−0.7	1.5	10	0.8%	0.20 [−0.76, 1.16]	+	+		+	+	
h. Armstrong 2016	−0.5	0.6	22	0	0.8	21	4.0%	−0.50 [−0.92, −0.08]	+	+	+	+	+	
q. Harrison 2007	−0.2	0.2	23	−0.3	0.7	18	6.2%	0.10 [−0.23, 0.43]	?	−	−	−	+	−
r. Cheng 2017 (D+E)	−0.1	0.1	29	0	0.1	15	58.0%	−0.10 [−0.16, −0.04]	+	+		+	+	−
u. Cheng 2017 (D)	−0.1	0.3	28	0	0.1	14	31.0%	−0.10 [−0.22, 0.02]	+	+		+	+	−
**Total (95% CI)**			**122**			**78**	**100.0%**	**−0.10 [−0.19, −0.01]**						

Heterogeneity: $Tau^2 = 0.00$; $Chi^2 = 5.18$, df = 4 (P = 0.27); $I^2 = 23\%$
Test for overall effect: Z = 2.29 (P = 0.02)

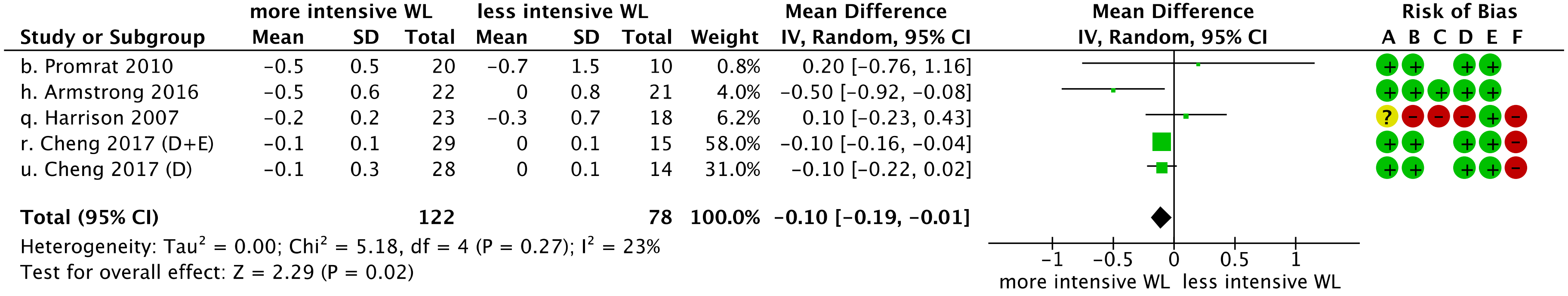

© 2019 Koutoukidis DA et al. *JAMA Internal Medicine*.

eFigure 1.3 Insulin resistance index (HOMA-IR, QUICKI, or Matsuda)

Study or Subgroup	more intensive WL Mean	SD	Total	less intensive WL Mean	SD	Total	Weight	Std. Mean Difference IV, Random, 95% CI	Risk of Bias A	B	C	D	E	F
a. Al–Jiffri 2013	−1.4	1.6	50	0.1	0.6	50	5.6%	−1.23 [−1.66, −0.80]	?	?			+	
b. Promrat 2010	−2	3	20	0.1	2.3	10	5.5%	−0.73 [−1.52, 0.05]	+	+		+	+	
c. AbdEl–Kader 2016	−2	0.7	50	0.3	0.5	50	5.6%	−3.75 [−4.41, −3.09]	?	+			?	
d. Sun 2012	−1.5	0.3	674	0.5	0.3	332	5.6%	−6.66 [−6.98, −6.34]	+	?		+	+	
f. Abenavoli 2017	0.2	0.5	20	0.6	0.3	10	5.5%	−0.87 [−1.67, −0.08]	−	?		+	+	
h. Armstrong 2016	−1.8	3.7	23	0.7	9.5	22	5.6%	−0.34 [−0.93, 0.25]	+	+	+	+	+	
i. Asghari 2018	−0.2	0.5	30	0.7	1.9	30	5.6%	−0.64 [−1.16, −0.12]	+	−	+	+	+	
l. Katsagoni 2018 (D)	−0.5	0.8	21	−0.3	0.6	11	5.5%	−0.26 [−1.00, 0.47]	+	+		+	−	
n. StGeorge 2009 (M)	−0.2	2.3	36	0	1.5	17	5.6%	−0.09 [−0.67, 0.48]	?	?			+	−
o. Katsagoni 2018 (D+E)	−0.8	0.5	21	−0.3	0.6	10	5.5%	−0.91 [−1.70, −0.12]	+	+		+	−	
p. Zelber–Sagi 2004	−6.2	19.6	21	−0.8	3	23	5.6%	−0.39 [−0.98, 0.21]	−	−	+	+	+	
q. Harrison 2007	0.01	0.014	23	0.01	0.007	18	5.6%	0.00 [−0.62, 0.62]	?	−	−	−	+	−
r. Cheng 2017 (D+E)	−0.1	0.5	29	0.2	0.6	15	5.6%	−0.55 [−1.19, 0.08]	+	+		+	+	−
t. StGeorge 2009 (L)	−0.4	1	73	0	1.5	17	5.6%	−0.36 [−0.89, 0.17]	?	?			+	−
u. Cheng 2017 (D)	0.2	0.5	28	0.2	0.6	14	5.6%	0.00 [−0.64, 0.64]	+	+		+	+	−
w. Eckard 2013 (MF)	−0.008	0.019	9	0	0.03	5	5.4%	−0.32 [−1.42, 0.78]	+	?		+	+	
x. Khoo 2017	−2.9	2.3	12	−3.2	2.6	12	5.5%	0.12 [−0.68, 0.92]	+	+	−	+	+	
y. Eckard 2013 (LF)	0.02	0.02	12	0	0.03	6	5.4%	0.81 [−0.22, 1.83]	+	?		+	+	
**Total (95% CI)**			**1152**			**652**	**100.0%**	**−0.91 [−2.11, 0.30]**						

Heterogeneity: $Tau^2 = 6.70$; $Chi^2 = 1219.38$, df = 17 ($P < 0.00001$); $I^2 = 99\%$

Test for overall effect: Z = 1.47 (P = 0.14)

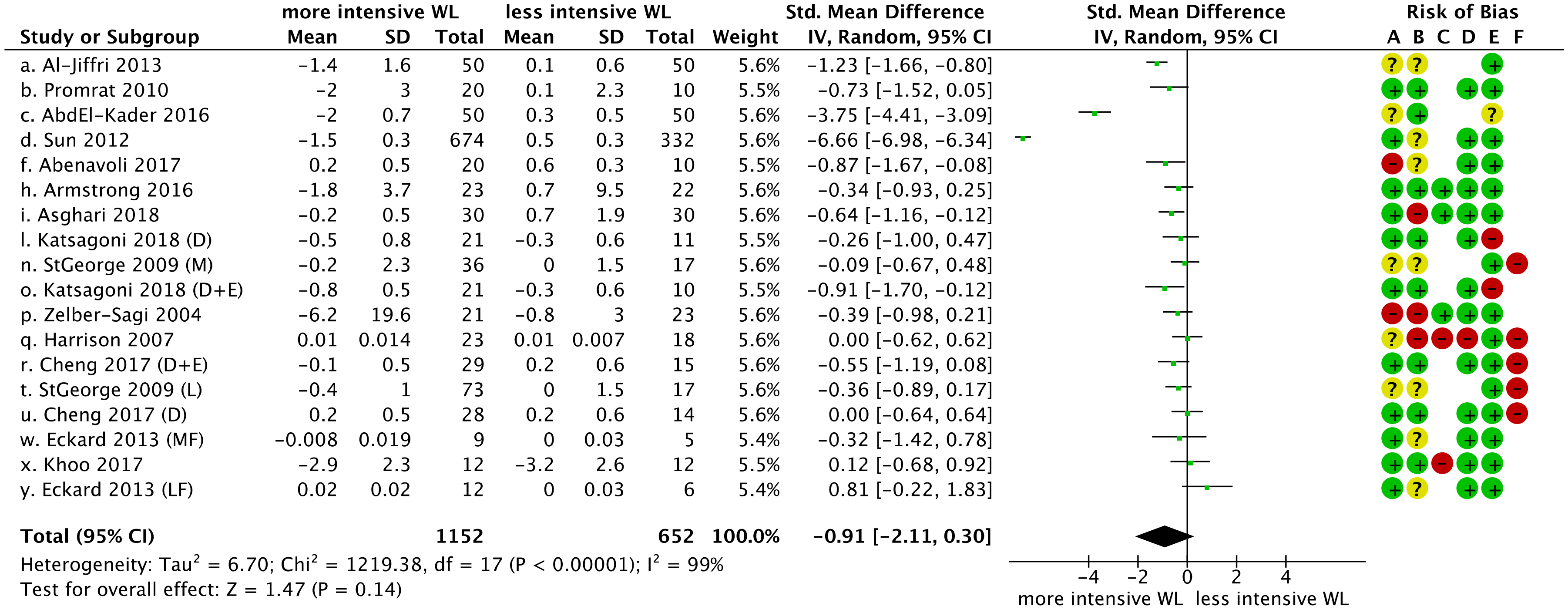

© 2019 Koutoukidis DA et al. *JAMA Internal Medicine*.

eFigure 1.4 Insulin (pmol/L)

Study or Subgroup	more intensive WL Mean	more intensive WL SD	more intensive WL Total	less intensive WL Mean	less intensive WL SD	less intensive WL Total	Weight	Mean Difference IV, Random, 95% CI	Risk of Bias A	B	C	D	E	F
b. Promrat 2010	-31.2	58.8	20	18	30.6	10	5.8%	-49.20 [-81.20, -17.20]	+	+		+	+	
d. Sun 2012	-27	31.8	674	22.2	25.2	332	8.2%	-49.20 [-52.82, -45.58]	+	?		+	+	
f. Abenavoli 2017	4.8	10.8	20	10.2	9	10	8.1%	-5.40 [-12.72, 1.92]	-	?		+	+	
h. Armstrong 2016	-15.9	54.7	23	-34.7	164.1	22	2.7%	18.80 [-53.32, 90.92]	+	+	+	+	+	
i. Asghari 2018	-7.8	12	30	16.8	52.8	30	7.2%	-24.60 [-43.98, -5.22]	+	-	+	+	+	
l. Katsagoni 2018 (D)	-30.8	16.6	21	-6.5	14	11	7.9%	-24.30 [-35.20, -13.40]	+	+		+	-	
n. StGeorge 2009 (M)	4.2	25.2	36	-1.8	27	17	7.5%	6.00 [-9.25, 21.25]	?	?			+	-
o. Katsagoni 2018 (D+E)	-14.3	18.7	21	-6.5	14	10	7.8%	-7.80 [-19.60, 4.00]	+	+		+	-	
p. Zelber-Sagi 2004	-81	235.8	21	-18.6	74.4	23	1.5%	-62.40 [-167.74, 42.94]	-	-	+	+	+	
q. Harrison 2007	-24	69.6	23	-6	16.8	18	6.1%	-18.00 [-47.48, 11.48]	?	-	-	-	+	-
r. Cheng 2017 (D+E)	-4.2	24	29	0.6	35.4	15	7.1%	-4.80 [-24.73, 15.13]	+	+		+	+	-
t. StGeorge 2009 (L)	-9	22.2	73	-1.8	27	17	7.7%	-7.20 [-21.01, 6.61]	?	?			+	-
u. Cheng 2017 (D)	-0.6	19.2	28	0.6	35.4	14	7.1%	-1.20 [-21.06, 18.66]	+	+		+	+	-
w. Eckard 2013 (MF)	15.6	36	9	-6.6	41.4	5	4.7%	22.20 [-21.04, 65.44]	+	?		+	+	
x. Khoo 2017	-54.6	45.6	12	-56.4	37.2	12	5.7%	1.80 [-31.50, 35.10]	+	+	-	+	+	
y. Eckard 2013 (LF)	-31.2	44.4	12	-6.6	41.4	6	4.9%	-24.60 [-66.17, 16.97]	+	?		+	+	
**Total (95% CI)**			**1052**			**552**	**100.0%**	**-13.75 [-28.04, 0.53]**						

Heterogeneity: Tau^2 = 643.40; Chi^2 = 222.99, df = 15 (P < 0.00001); I^2 = 93%

Test for overall effect: Z = 1.89 (P = 0.06)

Mean Difference IV, Random, 95% CI

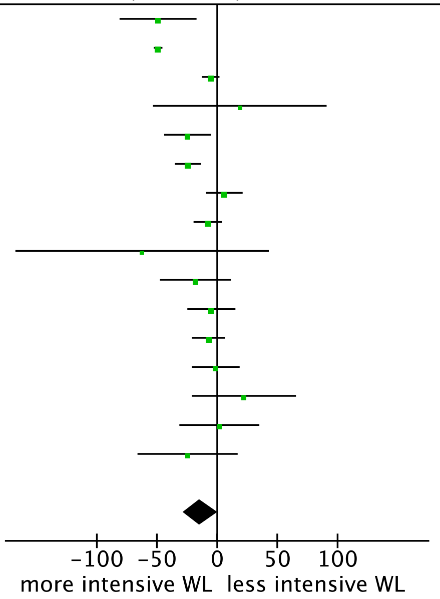

© 2019 Koutoukidis DA et al. *JAMA Internal Medicine*.

eFigure 1.5 Aspartate transaminase – AST (U/L)

Study or Subgroup	more intensive WL Mean	SD	Total	less intensive WL Mean	SD	Total	Weight	Mean Difference IV, Random, 95% CI	Risk of Bias A	B	C	D	E	F
a. Al-Jiffri 2013	-11.6	2.2	50	0.7	1.8	50	7.2%	-12.30 [-13.09, -11.51]	?	?			+	
b. Promrat 2010	-20.2	14.1	20	-18	30.8	10	1.3%	-2.20 [-22.26, 17.86]	+	+		+	+	
c. AbdEl-Kader 2016	-9.5	1.6	50	0.4	1.4	50	7.2%	-9.90 [-10.49, -9.31]	?	+			?	
d. Sun 2012	-5.7	15.2	674	-0.5	5.7	332	7.1%	-5.20 [-6.50, -3.90]	+	?		+	+	
e. Bahmanabadi 2011	4.4	6.7	20	-1.7	7.9	20	5.8%	6.10 [1.56, 10.64]	?	?	-	?	?	
f. Abenavoli 2017	2.8	6.3	20	12.4	15.2	10	3.4%	-9.60 [-19.42, 0.22]	-	?		+	+	
g. Wong 2013	-4	6.2	77	-3	5.4	77	7.0%	-1.00 [-2.84, 0.84]	+	+			+	
h. Armstrong 2016	-15.8	21.8	23	-8.6	28.3	22	2.0%	-7.20 [-22.01, 7.61]	+	+	+	+	+	
i. Asghari 2018	-4.1	3.6	30	4.4	11.8	30	5.9%	-8.50 [-12.91, -4.09]	+	-	+	+	+	
j. Axley 2017	-9	2.2	8	0	2	14	7.0%	-9.00 [-10.85, -7.15]	+	+			-	
k. Selezneva 2014	0.1	13.7	58	-11	16.4	116	5.8%	11.10 [6.48, 15.72]	?	?			+	
m. Lim 2018	-19.3	26.1	43	-5.1	13.7	43	3.8%	-14.20 [-23.01, -5.39]	?	?			?	-
n. StGeorge 2009 (M)	-6.9	18.3	36	-2.6	14.7	17	3.6%	-4.30 [-13.50, 4.90]	?	?			+	-
p. Zelber-Sagi 2004	-18.9	33	21	-8.8	17.2	23	1.8%	-10.10 [-25.87, 5.67]	-	-	+	+	+	
q. Harrison 2007	-28	23.2	23	-29	18.4	18	2.5%	1.00 [-11.73, 13.73]	?	-	-	-	+	-
r. Cheng 2017 (D+E)	-4.9	2.4	29	4	2.3	14	7.1%	-8.90 [-10.39, -7.41]	+	+		+	+	-
s. Dong 2016	-1	3.5	130	0.4	4.9	130	7.2%	-1.40 [-2.44, -0.36]	+	?		+	+	-
t. StGeorge 2009 (L)	-8.3	16.1	73	-2.6	14.7	17	4.2%	-5.70 [-13.60, 2.20]	?	?			+	-
u. Cheng 2017 (D)	0.4	2.6	28	4	2.3	15	7.1%	-3.60 [-5.11, -2.09]	+	+		+	+	-
w. Eckard 2013 (MF)	-19.6	47.9	9	-2.9	25.8	5	0.4%	-16.70 [-55.31, 21.91]	+	?		+	+	
x. Khoo 2017	-18	15	12	-23	24	12	1.8%	5.00 [-11.01, 21.01]	+	+	-	+	+	
y. Eckard 2013 (LF)	-15.9	19.1	12	-2.9	25.8	6	1.0%	-13.00 [-36.30, 10.30]	+	?		+	+	
**Total (95% CI)**			**1446**			**1031**	**100.0%**	**-4.84 [-7.31, -2.38]**						

Heterogeneity: $Tau^2 = 21.83$; $Chi^2 = 530.14$, df = 21 (P < 0.00001); $I^2 = 96\%$
Test for overall effect: Z = 3.85 (P = 0.0001)

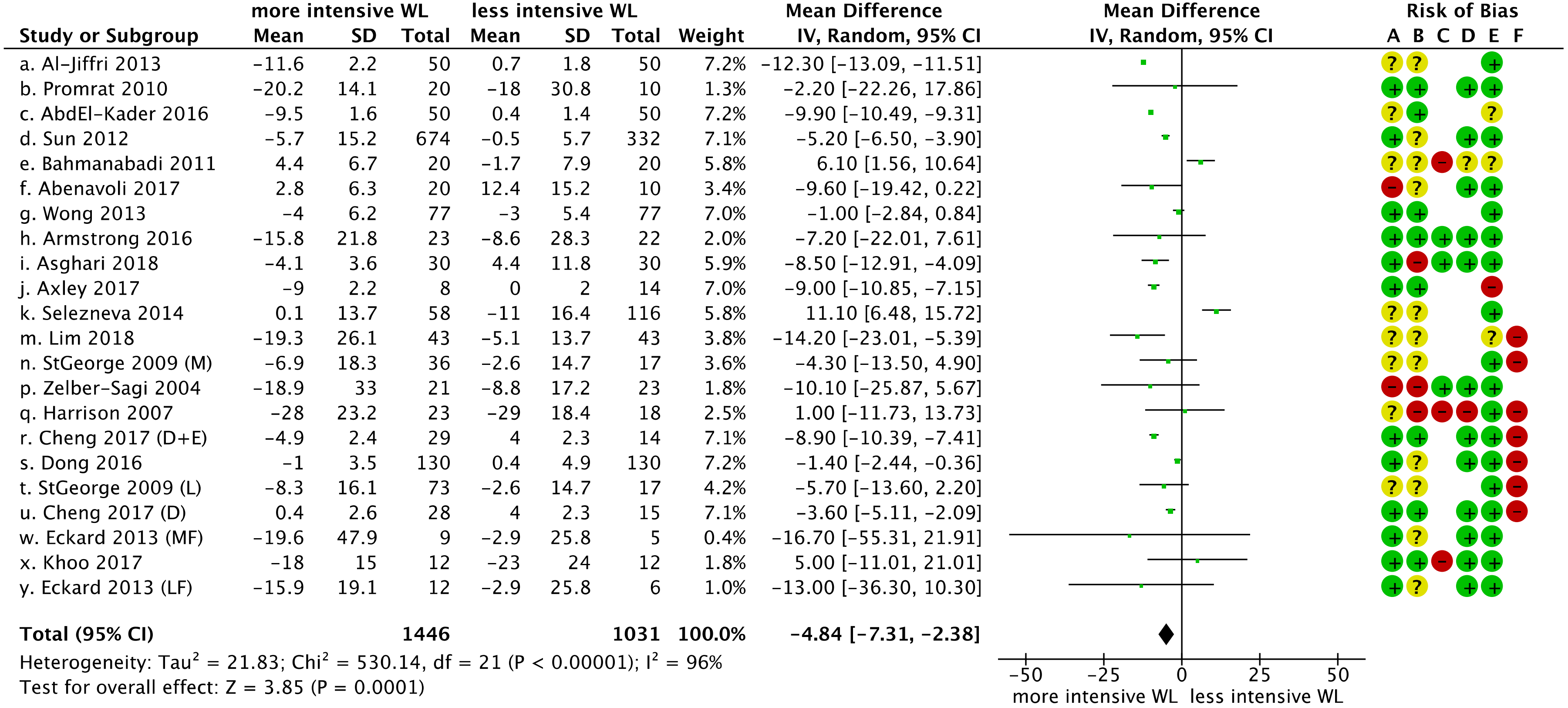

© 2019 Koutoukidis DA et al. *JAMA Internal Medicine*.

eFigure 1.6 Alkaline phosphatase – ALP (U/L)

Study or Subgroup	more intensive WL Mean	more intensive WL SD	more intensive WL Total	less intensive WL Mean	less intensive WL SD	less intensive WL Total	Weight	Mean Difference IV, Random, 95% CI	Risk of Bias A	B	C	D	E	F
a. Al-Jiffri 2013	-15.9	2.7	50	0.4	2.2	50	35.3%	-16.30 [-17.27, -15.33]	?	?			+	
h. Armstrong 2016	-5.1	11.7	23	-1.2	19.1	22	31.1%	-3.90 [-13.20, 5.40]	+	+	+	+	+	
q. Harrison 2007	-4	9.6	23	-8	9.3	18	33.6%	4.00 [-1.82, 9.82]	?	−	−	−	+	−
**Total (95% CI)**			**96**			**90**	**100.0%**	**-5.63 [-20.48, 9.22]**						

Heterogeneity: Tau^2 = 162.14; Chi^2 = 51.69, df = 2 (P < 0.00001); I^2 = 96%
Test for overall effect: Z = 0.74 (P = 0.46)

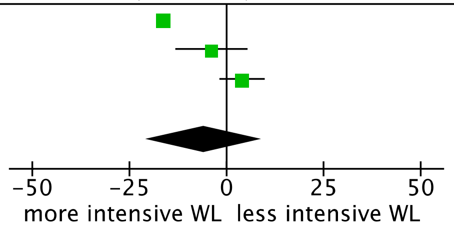

eFigure 1.7 γ-glutamyl transferase – GGT (U/L)

Study or Subgroup	more intensive WL Mean	more intensive WL SD	more intensive WL Total	less intensive WL Mean	less intensive WL SD	less intensive WL Total	Weight	Mean Difference IV, Random, 95% CI	Risk of Bias A	B	C	D	E	F
h. Armstrong 2016	-33.7	42.5	21	-7.2	28.3	21	2.0%	-26.50 [-48.34, -4.66]	+	+	+	+	+	
o. Katsagoni 2018 (D+E)	-27.5	37.2	21	-5.8	27.8	10	1.8%	-21.70 [-45.15, 1.75]	+	+		+	−	
l. Katsagoni 2018 (D)	-18	13.5	21	-5.8	27.8	11	2.9%	-12.20 [-29.61, 5.21]	+	+		+	−	
d. Sun 2012	-11.3	10.9	674	0.3	8.5	332	15.4%	-11.60 [-12.83, -10.37]	+	?		+	+	
p. Zelber-Sagi 2004	-22.3	42.2	21	-10.7	34.6	23	1.8%	-11.60 [-34.53, 11.33]	−	−	+	+	+	
a. Al-Jiffri 2013	-7.1	0.9	50	0.7	0.9	50	15.7%	-7.80 [-8.15, -7.45]	?	?			+	
t. StGeorge 2009 (L)	-16.1	35.3	73	-10.6	27	17	3.7%	-5.50 [-20.68, 9.68]	?	?			+	−
s. Dong 2016	-2.3	15	130	1	12.6	130	13.5%	-3.30 [-6.67, 0.07]	+	?		+	+	−
n. StGeorge 2009 (M)	-13	29.1	36	-10.6	27	17	3.4%	-2.40 [-18.37, 13.57]	?	?			+	−
f. Abenavoli 2017	4.4	2.1	20	5	5.4	10	13.4%	-0.60 [-4.07, 2.87]	−	?		+	+	
r. Cheng 2017 (D+E)	0.3	5.9	29	-2.6	5.5	15	13.4%	2.90 [-0.62, 6.42]	+	+		+	+	−
u. Cheng 2017 (D)	1.8	6.9	28	-2.6	5.5	14	13.0%	4.40 [0.55, 8.25]	+	+		+	+	−
**Total (95% CI)**			**1124**			**650**	**100.0%**	**-4.35 [-7.67, -1.04]**						

Heterogeneity: Tau^2 = 18.20; Chi^2 = 139.22, df = 11 (P < 0.00001); I^2 = 92%
Test for overall effect: Z = 2.57 (P = 0.01)

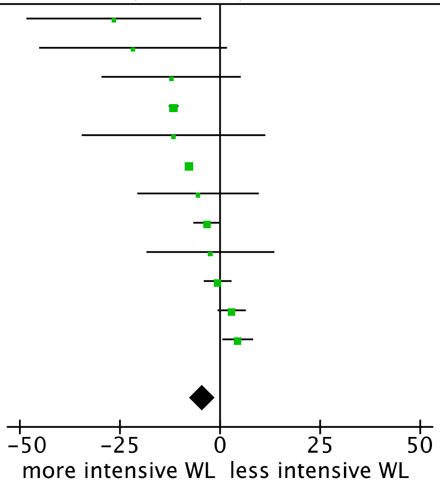

© 2019 Koutoukidis DA et al. *JAMA Internal Medicine*.

eFigure 1.8 NAFLD Fibrosis score

Study or Subgroup	more intensive WL Mean	SD	Total	less intensive WL Mean	SD	Total	Weight	Mean Difference IV, Random, 95% CI
l. Katsagoni 2018 (D)	0	0.4	21	0	0.4	10	31.3%	0.00 [−0.30, 0.30]
o. Katsagoni 2018 (D+E)	0	0.4	21	0	0.4	11	32.0%	0.00 [−0.29, 0.29]
s. Dong 2016	−4.7	1.1	130	−5.1	0.8	130	36.6%	0.40 [0.17, 0.63]
**Total (95% CI)**			**172**			**151**	**100.0%**	**0.15 [−0.13, 0.43]**

Heterogeneity: $Tau^2 = 0.04$; $Chi^2 = 6.23$, df = 2 (P = 0.04); $I^2 = 68\%$
Test for overall effect: Z = 1.03 (P = 0.31)

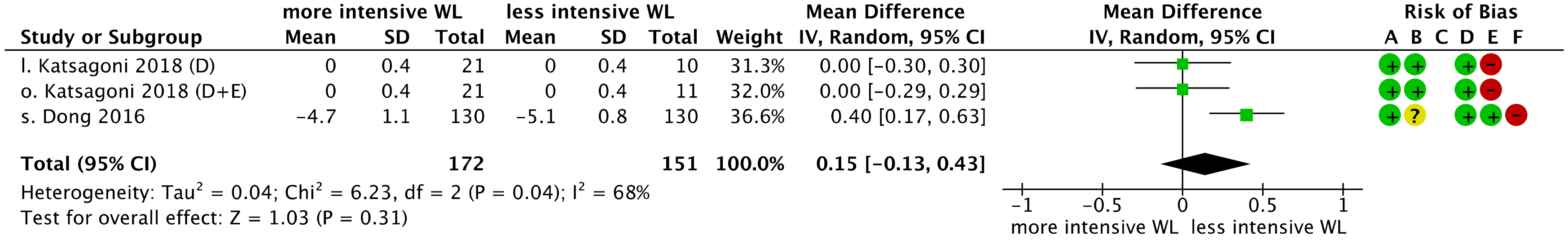

eFigure 1.9 Fatty liver index

Study or Subgroup	more intensive WL Mean	SD	Total	less intensive WL Mean	SD	Total	Weight	Std. Mean Difference IV, Random, 95% CI
f. Abenavoli 2017	−18	6.8	20	3	2.3	10	48.3%	−3.55 [−4.78, −2.33]
s. Dong 2016	0	10.8	130	2.4	9	130	51.7%	−0.24 [−0.48, 0.00]
**Total (95% CI)**			**150**			**140**	**100.0%**	**−1.84 [−5.08, 1.40]**

Heterogeneity: $Tau^2 = 5.28$; $Chi^2 = 26.91$, df = 1 (P < 0.00001); $I^2 = 96\%$
Test for overall effect: Z = 1.11 (P = 0.27)

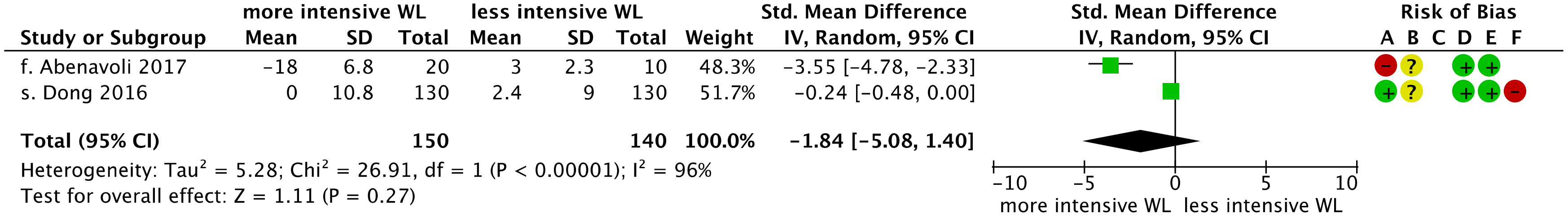

eFigure 1.10 Enhanced liver fibrosis (ELF) score

Study or Subgroup	more intensive WL Mean	SD	Total	less intensive WL Mean	SD	Total	Mean Difference IV, Fixed, 95% CI
h. Armstrong 2016	−0.3	0.8	23	0.1	0.8	22	−0.40 [−0.87, 0.07]

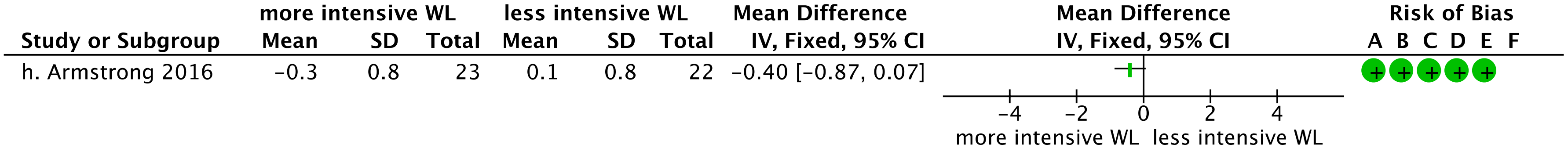

© 2019 Koutoukidis DA et al. *JAMA Internal Medicine*.

eFigure 1.11 Liver stiffness (kPa)

Study or Subgroup	more intensive WL Mean	more intensive WL SD	more intensive WL Total	less intensive WL Mean	less intensive WL SD	less intensive WL Total	Weight	Mean Difference IV, Random, 95% CI	Risk of Bias A	B	C	D	E	F
f. Abenavoli 2017	−1.7	0.6	20	0.4	0.6	10	24.0%	−2.10 [−2.56, −1.64]	−	?		+	+	
g. Wong 2013	−0.5	0.9	77	0.2	0.7	77	25.4%	−0.70 [−0.95, −0.45]	+	+			+	
l. Katsagoni 2018 (D)	−1.8	4.3	21	0.2	1.8	11	9.1%	−2.00 [−4.12, 0.12]	+	+		+	−	
o. Katsagoni 2018 (D+E)	−1.2	1.3	21	0.2	1.8	10	15.9%	−1.40 [−2.65, −0.15]	+	+		+	−	
x. Khoo 2017	−0.3	0.3	12	−0.2	0.2	12	25.6%	−0.10 [−0.30, 0.10]	+	+	−	+	+	
**Total (95% CI)**			**151**			**120**	**100.0%**	**−1.11 [−1.91, −0.32]**						

Heterogeneity: $Tau^2 = 0.63$; $Chi^2 = 68.09$, df = 4 (P < 0.00001); $I^2 = 94\%$
Test for overall effect: Z = 2.75 (P = 0.006)

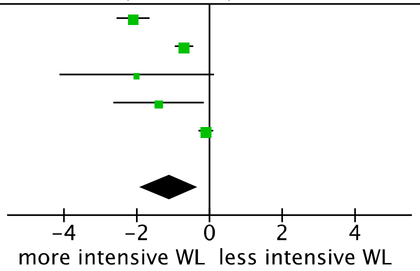

eFigure 1.12 Presence of definite NASH (Yes/No)

Study or Subgroup	more intensive WL Events	more intensive WL Total	less intensive WL Events	less intensive WL Total	Weight	Odds Ratio M-H, Random, 95% CI	Risk of Bias A	B	C	D	E	F
b. Promrat 2010	6	18	8	10	45.6%	0.13 [0.02, 0.78]	+	+		+	+	
h. Armstrong 2016	14	23	20	22	54.4%	0.16 [0.03, 0.83]	+	+	+	+	+	
**Total (95% CI)**		**41**		**32**	**100.0%**	**0.14 [0.04, 0.49]**						
Total events	20		28									

Heterogeneity: $Tau^2 = 0.00$; $Chi^2 = 0.03$, df = 1 (P = 0.86); $I^2 = 0\%$
Test for overall effect: Z = 3.10 (P = 0.002)

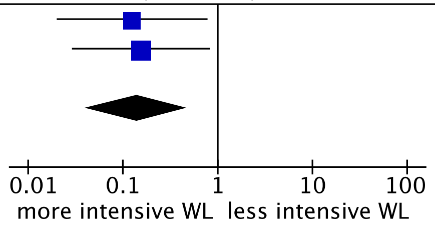

© 2019 Koutoukidis DA et al. *JAMA Internal Medicine*.

eFigure 1.13 Inflammation (score 0-3)

Study or Subgroup	more intensive WL Mean	SD	Total	less intensive WL Mean	SD	Total	Weight	Mean Difference IV, Random, 95% CI	Risk of Bias A	B	C	D	E	F
b. Promrat 2010	−0.5	0.3	18	−0.4	0.3	10	14.3%	−0.10 [−0.33, 0.13]	+	+		+	+	
h. Armstrong 2016	−0.2	0.6	23	−0.2	0.5	22	7.4%	0.00 [−0.32, 0.32]	+	+	+	+	+	
q. Harrison 2007	0	0.1	23	0	0.2	18	75.3%	0.00 [−0.10, 0.10]	?	−	−	−	+	−
v. Lee 2012	−1	0.7	8	−1	0.2	10	3.1%	0.00 [−0.50, 0.50]	+	?	+	?	+	
**Total (95% CI)**			**72**			**60**	**100.0%**	**−0.01 [−0.10, 0.07]**						

Heterogeneity: $Tau^2 = 0.00$; $Chi^2 = 0.61$, df = 3 (P = 0.89); $I^2 = 0\%$
Test for overall effect: Z = 0.32 (P = 0.75)

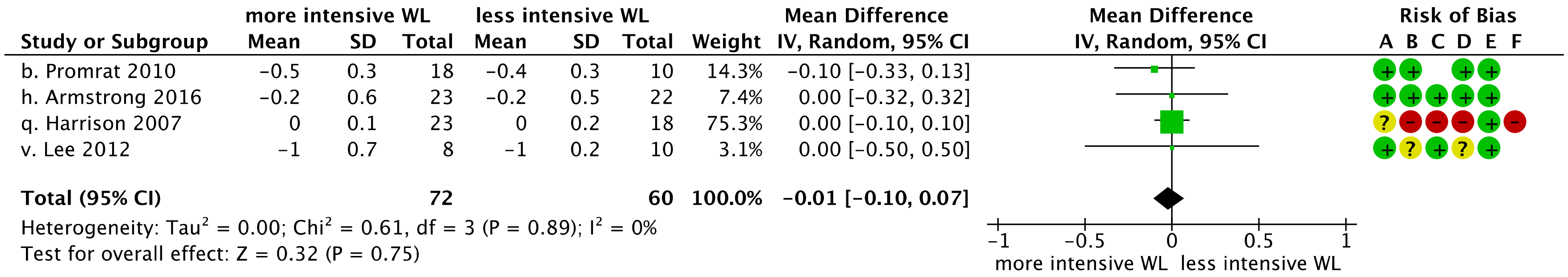

eFigure 1.14 Ballooning (score 0-2)

Study or Subgroup	more intensive WL Mean	SD	Total	less intensive WL Mean	SD	Total	Weight	Mean Difference IV, Random, 95% CI	Risk of Bias A	B	C	D	E	F
b. Promrat 2010	−0.9	0.2	18	−0.7	0.2	10	36.8%	−0.20 [−0.35, −0.05]	+	+		+	+	
h. Armstrong 2016	−0.5	0.7	23	−0.2	0.6	22	12.0%	−0.30 [−0.68, 0.08]	+	+	+	+	+	
q. Harrison 2007	0	0.2	23	0	0.2	18	43.3%	0.00 [−0.12, 0.12]	?	−	−	−	+	−
v. Lee 2012	0	0.7	8	0	0.00001	10	7.9%	0.00 [−0.49, 0.49]	+	?	+	?	+	
**Total (95% CI)**			**72**			**60**	**100.0%**	**−0.11 [−0.26, 0.04]**						

Heterogeneity: $Tau^2 = 0.01$; $Chi^2 = 5.29$, df = 3 (P = 0.15); $I^2 = 43\%$
Test for overall effect: Z = 1.47 (P = 0.14)

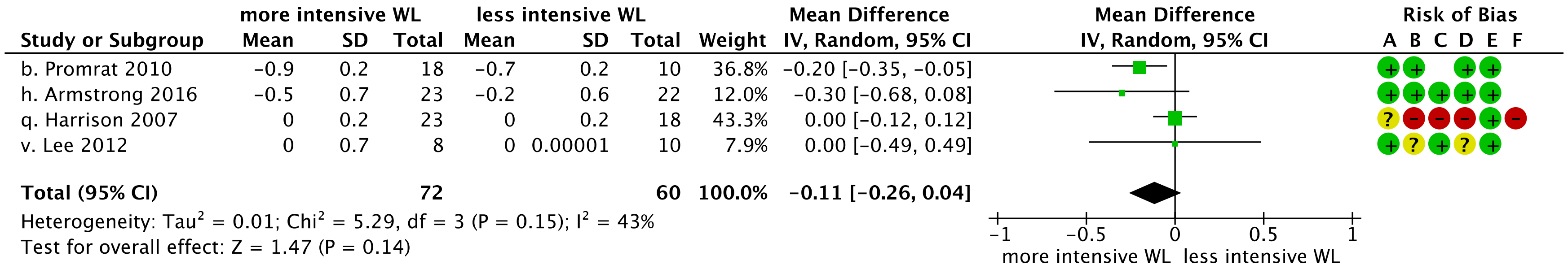

© 2019 Koutoukidis DA et al. *JAMA Internal Medicine*.

eFigure 1.15 Fibrosis (stage F0-F4)

	more intensive WL			less intensive WL				Mean Difference	Risk of Bias					
**Study or Subgroup**	**Mean**	**SD**	**Total**	**Mean**	**SD**	**Total**	**Weight**	**IV, Random, 95% CI**	**A**	**B**	**C**	**D**	**E**	**F**
b. Promrat 2010	0	0.5	18	−0.3	0.6	10	19.8%	0.30 [−0.14, 0.74]	+	+		+	+	
h. Armstrong 2016	−0.2	0.8	23	0.2	1	22	17.9%	−0.40 [−0.93, 0.13]	+	+	+	+	+	
p. Zelber-Sagi 2004	−0.7	0.6	11	−0.5	0.4	11	20.1%	−0.20 [−0.63, 0.23]	−	−	+	+	+	
v. Lee 2012	0.5	0.8	8	0	0.3	10	16.8%	0.50 [−0.08, 1.08]	+	?	+	?	+	
w. Eckard 2013 (MF)	−0.1	1.5	9	0.1	0.5	5	9.1%	−0.20 [−1.27, 0.87]	+	?		+	+	
y. Eckard 2013 (LF)	−0.8	0.8	12	0.1	0.5	6	16.4%	−0.90 [−1.50, −0.30]	+	?		+	+	
**Total (95% CI)**			**81**			**64**	**100.0%**	**−0.13 [−0.54, 0.27]**						

Heterogeneity: $Tau^2 = 0.16$; $Chi^2 = 15.46$, df = 5 (P = 0.009); $I^2 = 68\%$
Test for overall effect: Z = 0.65 (P = 0.52)

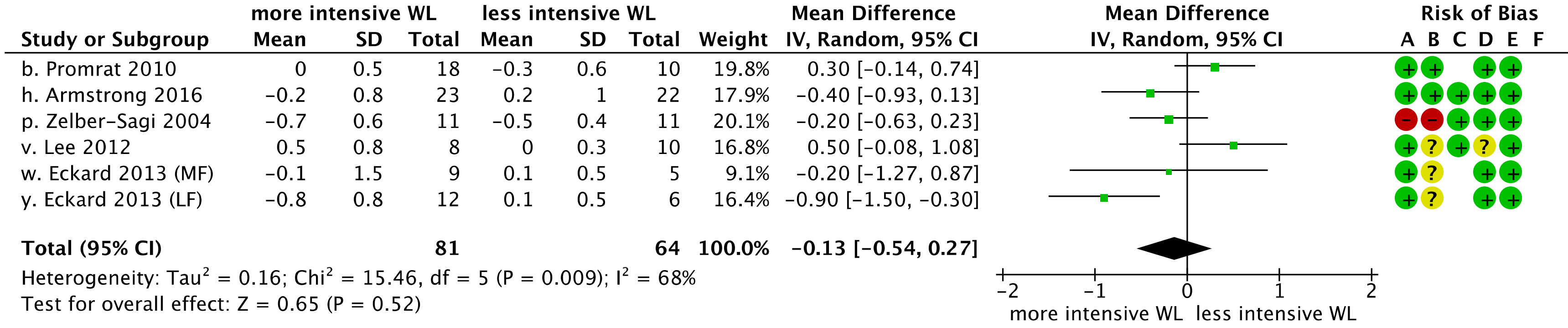

© 2019 Koutoukidis DA et al. *JAMA Internal Medicine*.

2 Effect of the weight loss interventions with long-term follow-up

eFigure 2.1 Weight (kg) (long-term)

	more intensive WL			less intensive WL				Mean Difference
Study or Subgroup	Mean	SD	Total	Mean	SD	Total	Weight	IV, Random, 95% CI
g. Wong 2013	−1.9	4.7	68	0.4	3.3	57	54.3%	−2.30 [−3.71, −0.89]
x. Khoo 2017	−1.8	3	13	−3.9	4.4	13	45.7%	2.10 [−0.79, 4.99]
**Total (95% CI)**			**81**			**70**	**100.0%**	**−0.29 [−4.59, 4.01]**

Heterogeneity: $Tau^2 = 8.33$; $Chi^2 = 7.18$, df = 1 (P = 0.007); $I^2 = 86\%$
Test for overall effect: Z = 0.13 (P = 0.90)

Mean Difference IV, Random, 95% CI — Risk of Bias A B C D E F

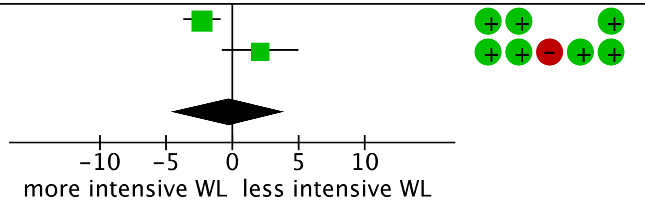

eFigure 2.2 Glucose (mmol/L) (long-term)

	more intensive WL			less intensive WL			Mean Difference
Study or Subgroup	Mean	SD	Total	Mean	SD	Total	IV, Random, 95% CI
g. Wong 2013	0.2	1	68	0.1	1.2	57	0.10 [−0.29, 0.49]

Mean Difference IV, Random, 95% CI — Risk of Bias A B C D E F

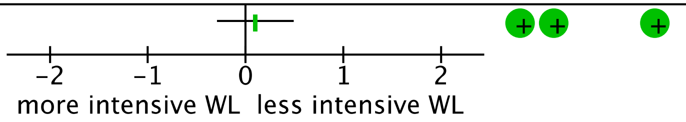

eFigure 2.3 HOMA-IR (long-term)

	more intensive WL			less intensive WL			Mean Difference
Study or Subgroup	Mean	SD	Total	Mean	SD	Total	IV, Random, 95% CI
x. Khoo 2017	−2.1	3.4	13	−3.8	2.6	13	1.70 [−0.63, 4.03]

Mean Difference IV, Random, 95% CI — Risk of Bias A B C D E F

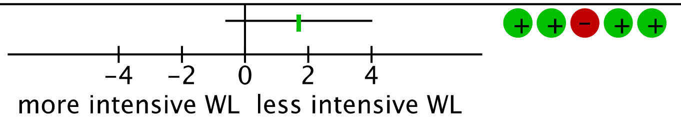

© 2019 Koutoukidis DA et al. *JAMA Internal Medicine*.

eFigure 2.4 Alanine aminotransferase – ALT (U/L) (long-term)

Study or Subgroup	more intensive WL Mean	SD	Total	less intensive WL Mean	SD	Total	Weight	Mean Difference IV, Random, 95% CI
g. Wong 2013	−9.1	33	68	−5.7	21.2	57	83.6%	−3.40 [−12.98, 6.18]
x. Khoo 2017	−22	43.6	13	−41.4	54.3	13	16.4%	19.40 [−18.45, 57.25]
**Total (95% CI)**			**81**			**70**	**100.0%**	**0.34 [−16.21, 16.90]**

Heterogeneity: $Tau^2 = 61.45$; $Chi^2 = 1.31$, df = 1 (P = 0.25); $I^2 = 24\%$
Test for overall effect: Z = 0.04 (P = 0.97)

Mean Difference IV, Random, 95% CI — Risk of Bias A B C D E F

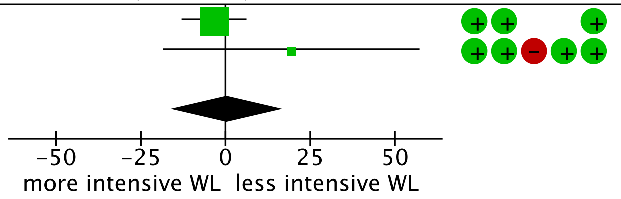

eFigure 2.5 Fatty liver index (long-term)

Study or Subgroup	more intensive WL Mean	SD	Total	less intensive WL Mean	SD	Total	Weight	Std. Mean Difference IV, Random, 95% CI
g. Wong 2013	36.5	31.5	68	38.5	29.6	57	100.0%	−0.06 [−0.42, 0.29]
**Total (95% CI)**			**68**			**57**	**100.0%**	**−0.06 [−0.42, 0.29]**

Heterogeneity: Not applicable
Test for overall effect: Z = 0.36 (P = 0.72)

Std. Mean Difference IV, Random, 95% CI — Risk of Bias A B C D E F

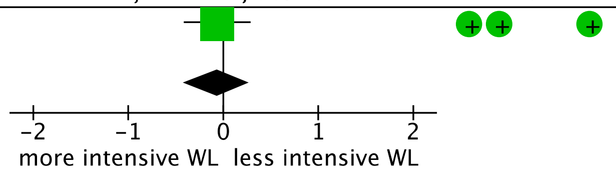

© 2019 Koutoukidis DA et al. *JAMA Internal Medicine*.

3 Risk of bias within and across studies

eFigure 3.1 Risk of bias within studies

	Random sequence generation (selection bias)	Allocation concealment (selection bias)	Blinding of participants and personnel (performance bias)	Blinding of subjective outcome assessments	Incomplete outcome data (attrition bias)	Other bias
a. Al-Jiffri 2013	?	?			+	
b. Katsagoni 2018 (D)	+	+		+	−	
c. Promrat 2010	+	+		+	+	
d. AbdEl-Kader 2016	?	+			?	
e. Sun 2012	+	?		+	+	
f. Bahmanabadi 2011	?	?	−	?	?	
g. Abenavoli 2017	−	?		+	+	
h. Wong 2013	+	+			+	
i. Armstrong 2016	+	+	+	+	+	
j. Asghari 2018	+	−	+	+	+	
k. Axley 2017	+	+			−	
l. Selezneva 2014	?	?			+	
m. Lim 2018	?	?			?	−
n. StGeorge 2009 (M)	?	?			+	−
o. Zelber-Sagi 2004	−	−	+	+	+	
p. Harrison 2007	?	−	−	−	+	−
q. Cheng 2017 (D+E)	+	+		+	+	−
r. Dong 2016	+	?		+	+	−
s. StGeorge 2009 (L)	?	?			+	−
t. Cheng 2017 (D)	+	+		+	+	−
u. Lee 2009	+	?	+	?	+	
v. Katsagoni 2018 (D+E)	+	+		+	−	
w. Eckard 2013 (MF)	+	?		+	+	
x. Khoo 2017	+	+	−	+	+	
y. Eckard 2013 (LF)	+	?		+	+	
z. Ye 2017	?	?	?	?	?	−

© 2019 Koutoukidis DA et al. *JAMA Internal Medicine*.

eFigure 3.2 Funnel plot of studies reporting (a) alanine aminotransferase (ALT) and (b) aspartate transaminase (AST)

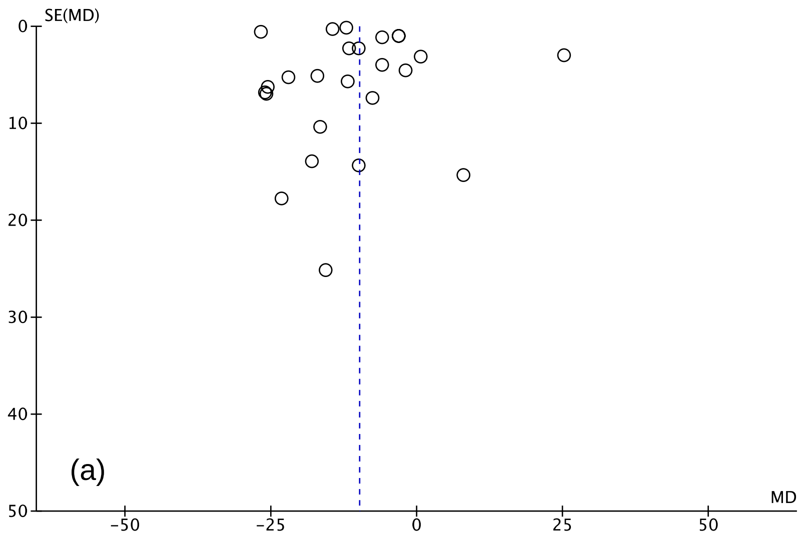

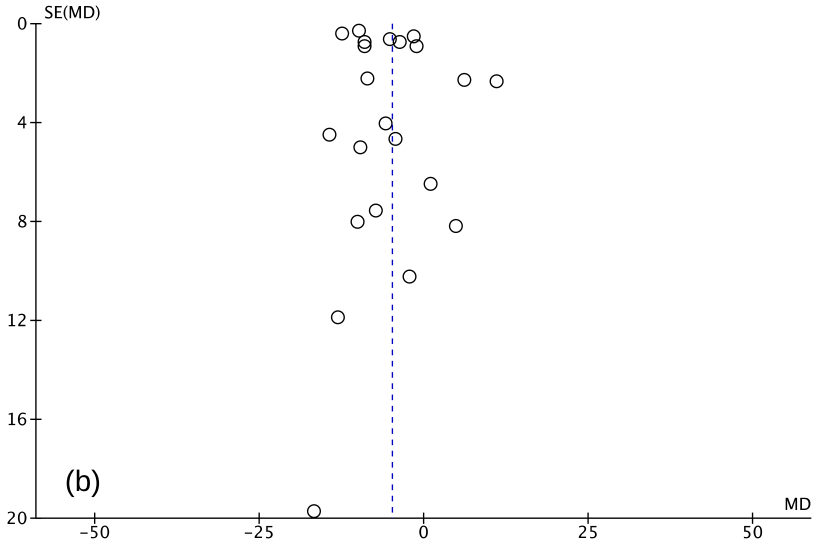

© 2019 Koutoukidis DA et al. *JAMA Internal Medicine*.

4 Sensitivity analysis including only the studies with low risk of bias

eFigure 4.1 Weight (kg) (sensitivity analysis)

Study or Subgroup	more intensive WL Mean	SD	Total	less intensive WL Mean	SD	Total	Weight	Mean Difference IV, Random, 95% CI	Risk of Bias A	B	C	D	E	F
b. Promrat 2010	−8.7	12.4	20	−0.5	7.4	10	3.3%	−8.20 [−15.31, −1.09]	+	+		+	+	
g. Wong 2013	−5.6	5.7	77	−0.6	3.7	77	72.9%	−5.00 [−6.52, −3.48]	+	+			+	
h. Armstrong 2016	−5.3	4.7	23	−0.6	4.4	22	23.8%	−4.70 [−7.36, −2.04]	+	+	+	+	+	
**Total (95% CI)**			**120**			**109**	**100.0%**	**−5.04 [−6.33, −3.74]**						

Heterogeneity: Tau^2 = 0.00; Chi^2 = 0.82, df = 2 (P = 0.66); I^2 = 0%
Test for overall effect: Z = 7.61 (P < 0.00001)

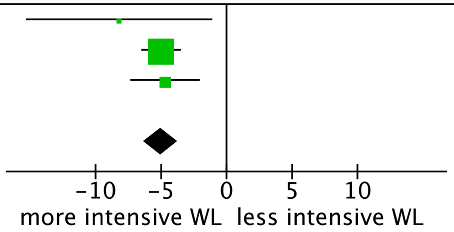

eFigure 4.2 HOMA-IR (sensitivity analysis)

Study or Subgroup	more intensive WL Mean	SD	Total	less intensive WL Mean	SD	Total	Weight	Std. Mean Difference IV, Random, 95% CI	Risk of Bias A	B	C	D	E	F
b. Promrat 2010	−2	3	20	0.1	2.3	10	36.1%	−0.73 [−1.52, 0.05]	+	+		+	+	
h. Armstrong 2016	−1.8	3.7	23	0.7	9.5	22	63.9%	−0.34 [−0.93, 0.25]	+	+	+	+	+	
**Total (95% CI)**			**43**			**32**	**100.0%**	**−0.48 [−0.95, −0.01]**						

Heterogeneity: Tau^2 = 0.00; Chi^2 = 0.60, df = 1 (P = 0.44); I^2 = 0%
Test for overall effect: Z = 2.01 (P = 0.04)

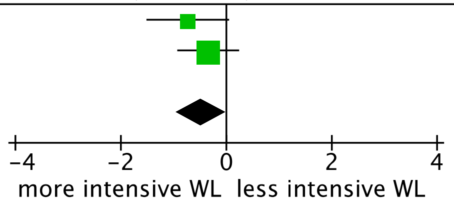

© 2019 Koutoukidis DA et al. *JAMA Internal Medicine*.

eFigure 4.3 Glucose (mmol/L) (sensitivity analysis)

	more intensive WL			less intensive WL				Mean Difference	Risk of Bias					
**Study or Subgroup**	**Mean**	**SD**	**Total**	**Mean**	**SD**	**Total**	**Weight**	**IV, Random, 95% CI**	**A**	**B**	**C**	**D**	**E**	**F**
b. Promrat 2010	−0.6	0.8	20	−1.7	3	10	22.2%	1.10 [−0.79, 2.99]	+	+		+	+	
g. Wong 2013	0	0.6	77	0.1	0.6	77	44.7%	−0.10 [−0.29, 0.09]	+	+			+	
h. Armstrong 2016	−1	1.5	23	0.7	2.3	23	33.1%	−1.70 [−2.82, −0.58]	+	+	+	+	+	
**Total (95% CI)**			**120**			**110**	**100.0%**	**−0.36 [−1.62, 0.89]**						

Heterogeneity: $Tau^2 = 0.90$; $Chi^2 = 9.24$, df = 2 (P = 0.010); $I^2 = 78\%$
Test for overall effect: Z = 0.57 (P = 0.57)

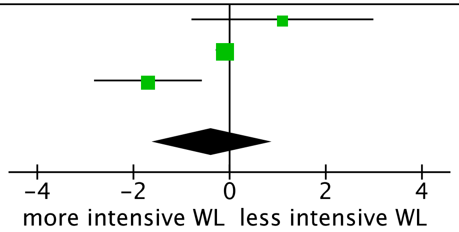

eFigure 4.4 Insulin (pmol/L) (sensitivity analysis)

	more intensive WL			less intensive WL				Mean Difference	Risk of Bias					
**Study or Subgroup**	**Mean**	**SD**	**Total**	**Mean**	**SD**	**Total**	**Weight**	**IV, Random, 95% CI**	**A**	**B**	**C**	**D**	**E**	**F**
b. Promrat 2010	−31.2	58.8	20	18	30.6	10	61.8%	−49.20 [−81.20, −17.20]	+	+		+	+	
h. Armstrong 2016	−15.9	54.7	23	−34.7	164.1	22	38.2%	18.80 [−53.32, 90.92]	+	+	+	+	+	
**Total (95% CI)**			**43**			**32**	**100.0%**	**−23.20 [−87.97, 41.57]**						

Heterogeneity: $Tau^2 = 1501.68$; $Chi^2 = 2.85$, df = 1 (P = 0.09); $I^2 = 65\%$
Test for overall effect: Z = 0.70 (P = 0.48)

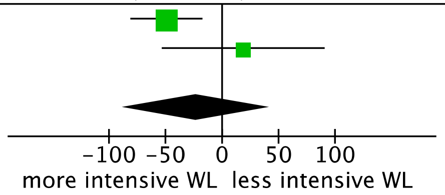

Figure 4.5 HbA1c (%) (sensitivity analysis)

	more intensive WL			less intensive WL				Mean Difference	Risk of Bias					
**Study or Subgroup**	**Mean**	**SD**	**Total**	**Mean**	**SD**	**Total**	**Weight**	**IV, Random, 95% CI**	**A**	**B**	**C**	**D**	**E**	**F**
b. Promrat 2010	−0.5	0.5	20	−0.7	1.5	10	30.5%	0.20 [−0.76, 1.16]	+	+		+	+	
h. Armstrong 2016	−0.5	0.6	22	0	0.8	21	69.5%	−0.50 [−0.92, −0.08]	+	+	+	+	+	
**Total (95% CI)**			**42**			**31**	**100.0%**	**−0.29 [−0.92, 0.35]**						

Heterogeneity: $Tau^2 = 0.10$; $Chi^2 = 1.72$, df = 1 (P = 0.19); $I^2 = 42\%$
Test for overall effect: Z = 0.89 (P = 0.37)

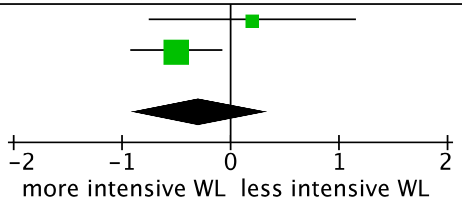

© 2019 Koutoukidis DA et al. *JAMA Internal Medicine*.

eFigure 4.6 Alanine aminotransferase – ALT (U/L) (sensitivity analysis)

Study or Subgroup	more intensive WL Mean	SD	Total	less intensive WL Mean	SD	Total	Weight	Mean Difference IV, Random, 95% CI
b. Promrat 2010	−42.4	23	20	−16.5	14.2	10	30.4%	−25.90 [−39.28, −12.52]
g. Wong 2013	−17	17.7	77	−7	9.5	77	50.8%	−10.00 [−14.49, −5.51]
h. Armstrong 2016	−26.6	34.4	23	−10.2	35.8	22	18.9%	−16.40 [−36.93, 4.13]
**Total (95% CI)**			**120**			**109**	**100.0%**	**−16.04 [−27.01, −5.07]**

Heterogeneity: $Tau^2 = 56.48$; $Chi^2 = 5.08$, df = 2 (P = 0.08); $I^2 = 61\%$
Test for overall effect: Z = 2.87 (P = 0.004)

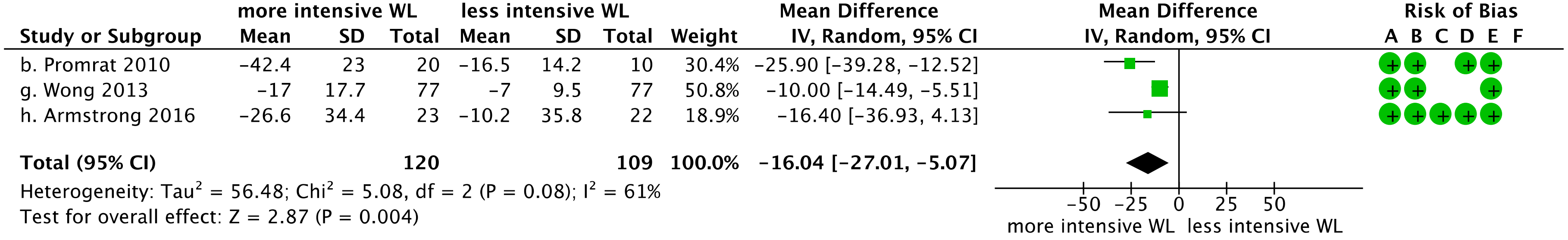

eFigure 4.7 Liver stiffness (kPa) (sensitivity analysis)

Study or Subgroup	more intensive WL Mean	SD	Total	less intensive WL Mean	SD	Total	Mean Difference IV, Random, 95% CI
g. Wong 2013	−0.5	0.9	77	0.2	0.7	77	−0.70 [−0.95, −0.45]

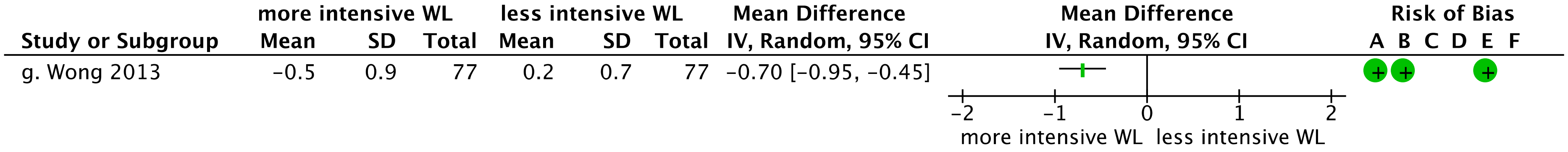

eFigure 4.8 Steatosis – Standardized Mean Difference as Assessed by Histologic Examination or MRI (Sensitivity Analysis)

Study or Subgroup	more intensive WL Mean	SD	Total	less intensive WL Mean	SD	Total	Weight	Std. Mean Difference IV, Random, 95% CI
b. Promrat 2010	−1.1	0.4	18	−0.3	0.4	10	28.3%	−1.94 [−2.89, −0.99]
g. Wong 2013	−6.8	3.1	77	−2.1	2.5	77	37.4%	−1.66 [−2.03, −1.29]
h. Armstrong 2016	−0.7	0.8	23	−0.4	0.8	22	34.3%	−0.37 [−0.96, 0.22]
**Total (95% CI)**			**118**			**109**	**100.0%**	**−1.30 [−2.24, −0.35]**

Heterogeneity: $Tau^2 = 0.58$; $Chi^2 = 14.87$, df = 2 (P = 0.0006); $I^2 = 87\%$
Test for overall effect: Z = 2.70 (P = 0.007)

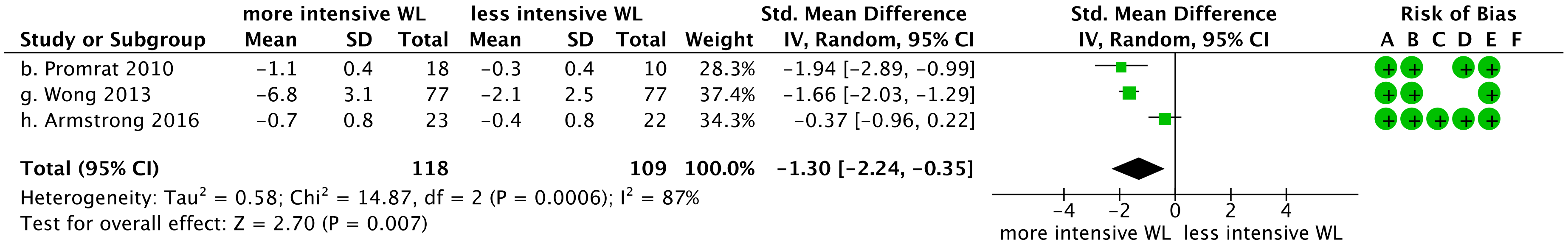

© 2019 Koutoukidis DA et al. *JAMA Internal Medicine*.

eFigure 4.9 NAFLD activity score (NAS) (sensitivity analysis)

Study or Subgroup	more intensive WL Mean	more intensive WL SD	more intensive WL Total	less intensive WL Mean	less intensive WL SD	less intensive WL Total	Weight	Mean Difference IV, Random, 95% CI
b. Promrat 2010	−2.4	0.7	18	−1.4	0.9	10	62.0%	−1.00 [−1.64, −0.36]
h. Armstrong 2016	−1.3	1.6	23	−0.8	1.2	22	38.0%	−0.50 [−1.32, 0.32]
**Total (95% CI)**			**41**			**32**	**100.0%**	**−0.81 [−1.32, −0.30]**

Heterogeneity: $Tau^2 = 0.00$; $Chi^2 = 0.88$, df = 1 (P = 0.35); $I^2 = 0\%$
Test for overall effect: Z = 3.13 (P = 0.002)

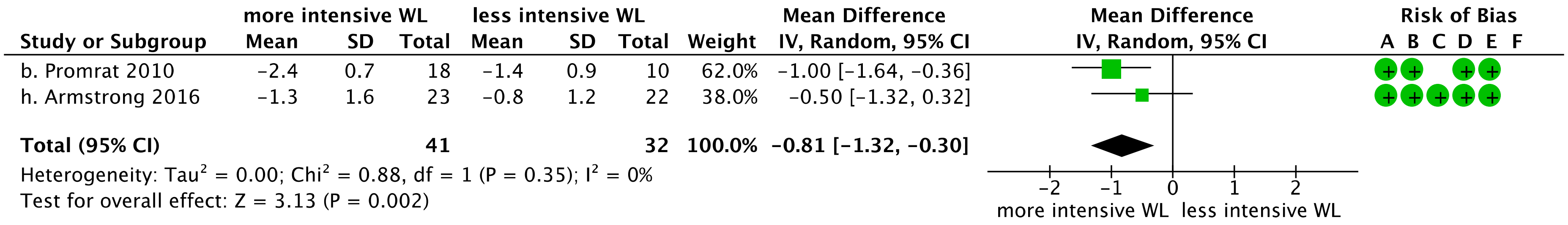

eFigure 4.10 Inflammation (score 0-3) (sensitivity analysis)

Study or Subgroup	more intensive WL Mean	more intensive WL SD	more intensive WL Total	less intensive WL Mean	less intensive WL SD	less intensive WL Total	Weight	Mean Difference IV, Random, 95% CI
b. Promrat 2010	−0.5	0.3	18	−0.4	0.3	10	65.9%	−0.10 [−0.33, 0.13]
h. Armstrong 2016	−0.2	0.6	23	−0.2	0.5	22	34.1%	0.00 [−0.32, 0.32]
**Total (95% CI)**			**41**			**32**	**100.0%**	**−0.07 [−0.25, 0.12]**

Heterogeneity: $Tau^2 = 0.00$; $Chi^2 = 0.24$, df = 1 (P = 0.62); $I^2 = 0\%$
Test for overall effect: Z = 0.69 (P = 0.49)

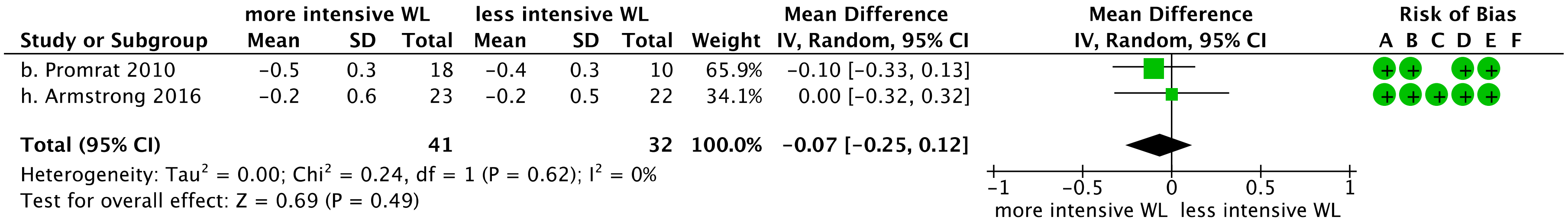

eFigure 4.11 Ballooning (score 0-2) (sensitivity analysis)

Study or Subgroup	more intensive WL Mean	more intensive WL SD	more intensive WL Total	less intensive WL Mean	less intensive WL SD	less intensive WL Total	Weight	Mean Difference IV, Random, 95% CI
b. Promrat 2010	−0.9	0.2	18	−0.7	0.2	10	85.8%	−0.20 [−0.35, −0.05]
h. Armstrong 2016	−0.5	0.7	23	−0.2	0.6	22	14.2%	−0.30 [−0.68, 0.08]
**Total (95% CI)**			**41**			**32**	**100.0%**	**−0.21 [−0.36, −0.07]**

Heterogeneity: $Tau^2 = 0.00$; $Chi^2 = 0.23$, df = 1 (P = 0.63); $I^2 = 0\%$
Test for overall effect: Z = 2.93 (P = 0.003)

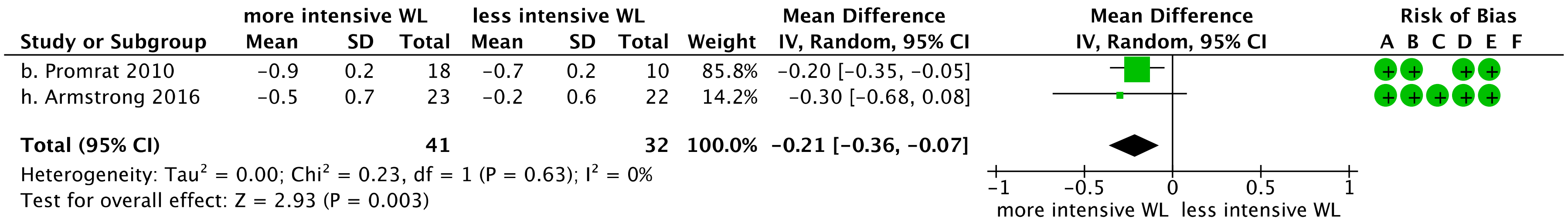

© 2019 Koutoukidis DA et al. *JAMA Internal Medicine*.

eFigure 4.12 Fibrosis (scale F0-F4) (sensitivity analysis)

	more intensive WL			less intensive WL				Mean Difference	Risk of Bias					
Study or Subgroup	Mean	SD	Total	Mean	SD	Total	Weight	IV, Random, 95% CI	A	B	C	D	E	F
h. Armstrong 2016	−0.2	0.8	23	0.2	1	22	47.6%	−0.40 [−0.93, 0.13]	+	+	+	+	+	
b. Promrat 2010	0	0.5	18	−0.3	0.6	10	52.4%	0.30 [−0.14, 0.74]	+	+		+	+	
**Total (95% CI)**			**41**			**32**	**100.0%**	**−0.03 [−0.72, 0.65]**						

Heterogeneity: $Tau^2 = 0.18$; $Chi^2 = 3.98$, df = 1 (P = 0.05); $I^2 = 75\%$
Test for overall effect: Z = 0.10 (P = 0.92)

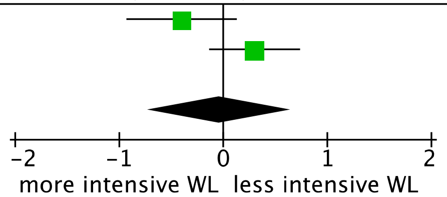

eFigure 4.13 Aspartate transaminase – AST (U/L) (sensitivity analysis)

	more intensive WL			less intensive WL				Mean Difference	Risk of Bias					
Study or Subgroup	Mean	SD	Total	Mean	SD	Total	Weight	IV, Random, 95% CI	A	B	C	D	E	F
b. Promrat 2010	−20.2	14.1	20	−18	30.8	10	0.8%	−2.20 [−22.26, 17.86]	+	+		+	+	
g. Wong 2013	−4	6.2	77	−3	5.4	77	97.7%	−1.00 [−2.84, 0.84]	+	+			+	
h. Armstrong 2016	−15.8	21.8	23	−8.6	28.3	22	1.5%	−7.20 [−22.01, 7.61]	+	+	+	+	+	
**Total (95% CI)**			**120**			**109**	**100.0%**	**−1.10 [−2.92, 0.71]**						

Heterogeneity: $Tau^2 = 0.00$; $Chi^2 = 0.67$, df = 2 (P = 0.71); $I^2 = 0\%$
Test for overall effect: Z = 1.19 (P = 0.23)

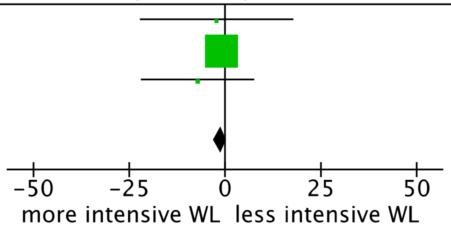

eFigure 4.14 Alkaline phosphatase – ALP (U/L) (sensitivity analysis)

	more intensive WL			less intensive WL			Mean Difference	Risk of Bias					
Study or Subgroup	Mean	SD	Total	Mean	SD	Total	IV, Random, 95% CI	A	B	C	D	E	F
h. Armstrong 2016	−5.1	11.7	23	−1.2	19.1	22	−3.90 [−13.20, 5.40]	+	+	+	+	+	

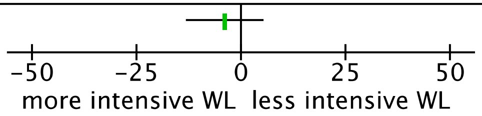

© 2019 Koutoukidis DA et al. *JAMA Internal Medicine*.

eFigure 4.15 γ-glutamyl transferase – GGT (U/L) (sensitivity analysis)

	more intensive WL			less intensive WL			Mean Difference	Mean Difference	Risk of Bias
Study or Subgroup	Mean	SD	Total	Mean	SD	Total	IV, Random, 95% CI	IV, Random, 95% CI	A B C D E F
h. Armstrong 2016	−33.7	42.5	21	−7.2	28.3	21	−26.50 [−48.34, −4.66]	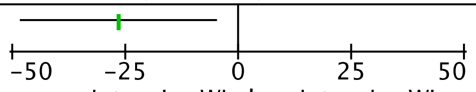	+ + + + +

© 2019 Koutoukidis DA et al. *JAMA Internal Medicine*.

5 Subgroup analysis by the type of intervention (behavioral, pharmacotherapy, surgery)

eFigure 5.1 Weight (kg) (subgroup analysis by the type of intervention)

Study or Subgroup	more intensive WL Mean	SD	Total	less intensive WL Mean	SD	Total	Weight	Mean Difference IV, Random, 95% CI
**4.6.1 Behavioural programmes**								
a. Al-Jiffri 2013	-13.7	3.4	50	0.8	3	50	4.7%	-14.50 [-15.76, -13.24]
b. Promrat 2010	-8.7	12.4	20	-0.5	7.4	10	2.4%	-8.20 [-15.31, -1.09]
c. AbdEl-Kader 2016	-5.9	2.2	50	2.1	2.9	50	4.8%	-8.00 [-9.01, -6.99]
d. Sun 2012	-6.1	9.2	674	1.3	9.2	332	4.7%	-7.40 [-8.61, -6.19]
f. Abenavoli 2017	-5.1	3.4	20	0.4	2.3	10	4.5%	-5.50 [-7.56, -3.44]
g. Wong 2013	-5.6	5.7	77	-0.6	3.7	77	4.6%	-5.00 [-6.52, -3.48]
i. Asghari 2018	-4.1	4	30	0	2.7	30	4.6%	-4.10 [-5.83, -2.37]
j. Axley 2017	-2.7	5	8	0.9	6	14	3.3%	-3.60 [-8.28, 1.08]
k. Selezneva 2014	-9.3	1.8	58	-6.2	1.7	116	4.8%	-3.10 [-3.66, -2.54]
l. Katsagoni 2018 (D)	-5.6	4.1	21	-2.8	3.9	11	4.1%	-2.80 [-5.70, 0.10]
m. Lim 2018	-3.2	2.5	43	-0.9	2	43	4.8%	-2.30 [-3.26, -1.34]
n. StGeorge 2009 (M)	-2.8	3.4	73	-0.5	3.7	17	4.5%	-2.30 [-4.22, -0.38]
o. Katsagoni 2018 (D+E)	-4.9	4.1	21	-2.8	3.9	10	4.1%	-2.10 [-5.09, 0.89]
r. Cheng 2017 (D+E)	-1.5	3.2	29	0.2	3.3	15	4.5%	-1.70 [-3.74, 0.34]
s. Dong 2016	-1.4	4.9	130	0.1	4.1	130	4.7%	-1.50 [-2.60, -0.40]
t. StGeorge 2009 (L)	-1.9	3.7	36	-0.5	3.7	17	4.4%	-1.40 [-3.53, 0.73]
u. Cheng 2017 (D)	-0.8	3.1	28	0.2	3.3	14	4.5%	-1.00 [-3.08, 1.08]
w. Eckard 2013 (MF)	-3	4.7	9	-2.5	5.3	5	3.0%	-0.50 [-6.07, 5.07]
y. Eckard 2013 (LF)	-0.2	5.4	12	-2.5	5.3	6	3.1%	2.30 [-2.93, 7.53]
**Subtotal (95% CI)**			**1389**			**957**	**80.2%**	**-3.93 [-5.67, -2.20]**
Heterogeneity: $Tau^2 = 12.99$; $Chi^2 = 430.74$, df = 18 (P < 0.00001); $I^2 = 96\%$								
Test for overall effect: Z = 4.44 (P < 0.00001)								
**4.6.2 Pharmacotherapy**								
e. Bahmanabadi 2011	-13	10	20	-6.1	3	20	3.4%	-6.90 [-11.48, -2.32]
h. Armstrong 2016	-5.3	4.7	23	-0.6	4.4	22	4.2%	-4.70 [-7.36, -2.04]
p. Zelber-Sagi 2004	-7.7	6.8	21	-5.9	5.9	23	3.8%	-1.80 [-5.58, 1.98]
q. Harrison 2007	-8.2	5	23	-6.4	5.2	18	4.0%	-1.80 [-4.95, 1.35]
x. Khoo 2017	-3.5	2.1	12	-3.5	3.3	12	4.4%	0.00 [-2.21, 2.21]
**Subtotal (95% CI)**			**99**			**95**	**19.8%**	**-2.75 [-5.10, -0.40]**
Heterogeneity: $Tau^2 = 4.50$; $Chi^2 = 11.33$, df = 4 (P = 0.02); $I^2 = 65\%$								
Test for overall effect: Z = 2.29 (P = 0.02)								
**Total (95% CI)**			**1488**			**1052**	**100.0%**	**-3.73 [-5.26, -2.20]**
Heterogeneity: $Tau^2 = 12.51$; $Chi^2 = 451.09$, df = 23 (P < 0.00001); $I^2 = 95\%$								
Test for overall effect: Z = 4.78 (P < 0.00001)								
Test for subgroup differences: $Chi^2 = 0.63$, df = 1 (P = 0.43), $I^2 = 0\%$								

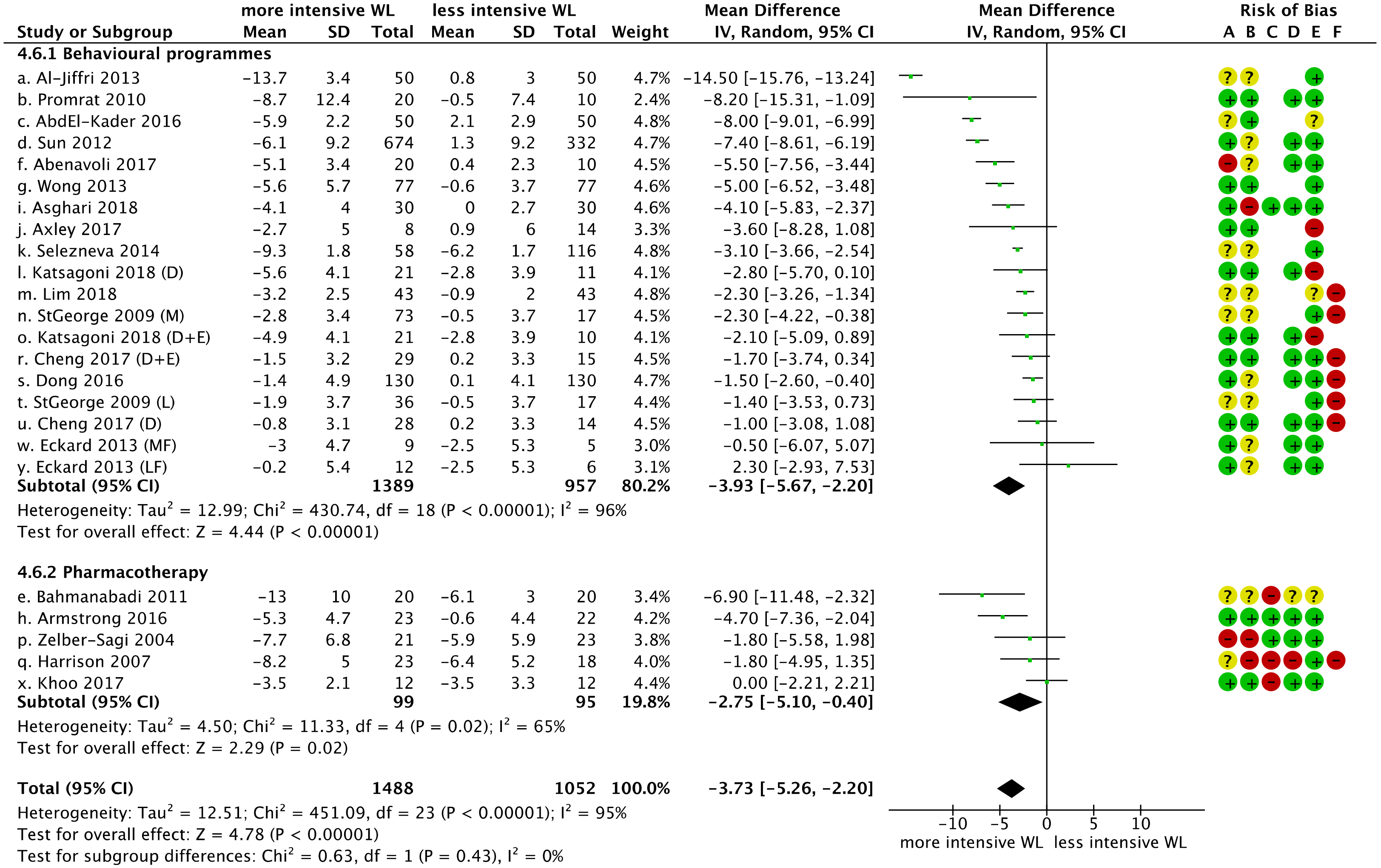

© 2019 Koutoukidis DA et al. *JAMA Internal Medicine*.

eFigure 5.2 Glucose (mmol/L) (subgroup analysis by the type of intervention)

Study or Subgroup	more intensive WL Mean	SD	Total	less intensive WL Mean	SD	Total	Weight	Mean Difference IV, Random, 95% CI	Risk of Bias A	B	C	D	E	F
**4.4.1 Behavioural programmes**														
b. Promrat 2010	−0.6	0.8	20	−1.7	3	10	1.2%	1.10 [−0.79, 2.99]	+	+		+	+	
d. Sun 2012	−1.6	0.7	674	−0.2	1	332	6.9%	−1.40 [−1.52, −1.28]	+	?		+	+	
f. Abenavoli 2017	−0.2	0.2	20	0	0.1	10	6.9%	−0.20 [−0.31, −0.09]	−	?		+	+	
g. Wong 2013	0	0.6	77	0.1	0.6	77	6.7%	−0.10 [−0.29, 0.09]	+	+			+	
i. Asghari 2018	0.2	0.2	30	0.1	0.1	30	7.0%	0.10 [0.02, 0.18]	+	−	+	+	+	
l. Katsagoni 2018 (D)	0	0.2	21	0.1	0.1	11	6.9%	−0.10 [−0.20, 0.00]	+	+		+	−	
n. StGeorge 2009 (M)	−0.2	0.7	73	0	0.7	17	5.9%	−0.20 [−0.57, 0.17]	?	?			+	−
o. Katsagoni 2018 (D+E)	−0.1	0.1	21	0.1	0.1	10	7.0%	−0.20 [−0.28, −0.12]	+	+		+	−	
r. Cheng 2017 (D+E)	0.1	0.2	29	0.2	0.2	15	6.9%	−0.10 [−0.22, 0.02]	+	+		+	+	−
s. Dong 2016	0	0.5	130	0.2	0.5	130	6.9%	−0.20 [−0.32, −0.08]	+	?		+	+	−
t. StGeorge 2009 (L)	−0.3	0.6	36	0	0.7	17	5.9%	−0.30 [−0.69, 0.09]	?	?			+	−
u. Cheng 2017 (D)	0.3	0.2	28	0.2	0.2	14	6.9%	0.10 [−0.03, 0.23]	+	+		+	+	−
w. Eckard 2013 (MF)	−0.1	1.1	9	0.1	0.5	5	3.6%	−0.20 [−1.04, 0.64]	+	?		+	+	
y. Eckard 2013 (LF)	−0.5	1.3	12	0.1	0.5	6	3.6%	−0.60 [−1.44, 0.24]	+	?		+	+	
**Subtotal (95% CI)**			**1180**			**684**	**82.4%**	**−0.23 [−0.47, 0.00]**						
Heterogeneity: $Tau^2 = 0.17$; $Chi^2 = 470.16$, df = 13 (P < 0.00001); $I^2 = 97\%$														
Test for overall effect: Z = 1.92 (P = 0.05)														
**4.4.2 Pharmacotherapy**														
h. Armstrong 2016	−1	1.5	23	0.7	2.3	23	2.6%	−1.70 [−2.82, −0.58]	+	+	+	+	+	
p. Zelber-Sagi 2004	−7.1	0.9	21	−5.1	0.3	23	5.8%	−2.00 [−2.40, −1.60]	−	−	+	+	+	
q. Harrison 2007	−0.4	0.2	23	−0.7	1.8	18	3.6%	0.30 [−0.54, 1.14]	?	−	−	−	+	−
x. Khoo 2017	−0.7	0.6	12	−0.6	0.5	12	5.6%	−0.10 [−0.54, 0.34]	+	+	−	+	+	
**Subtotal (95% CI)**			**79**			**76**	**17.6%**	**−0.87 [−2.10, 0.37]**						
Heterogeneity: $Tau^2 = 1.44$; $Chi^2 = 50.25$, df = 3 (P < 0.00001); $I^2 = 94\%$														
Test for overall effect: Z = 1.38 (P = 0.17)														
**Total (95% CI)**			**1259**			**760**	**100.0%**	**−0.34 [−0.57, −0.12]**						
Heterogeneity: $Tau^2 = 0.19$; $Chi^2 = 554.22$, df = 17 (P < 0.00001); $I^2 = 97\%$														
Test for overall effect: Z = 2.96 (P = 0.003)														
Test for subgroup differences: $Chi^2 = 0.99$, df = 1 (P = 0.32), $I^2 = 0\%$														

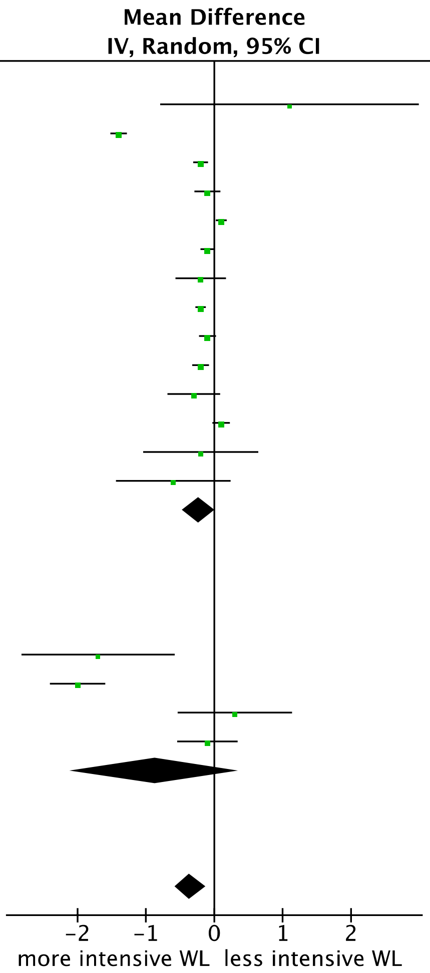

© 2019 Koutoukidis DA et al. *JAMA Internal Medicine*.

eFigure 5.3 Insulin resistance index (HOMA-IR or other) (subgroup analysis by the type of intervention)

Study or Subgroup	more intensive WL Mean	more intensive WL SD	more intensive WL Total	less intensive WL Mean	less intensive WL SD	less intensive WL Total	Weight	Std. Mean Difference IV, Random, 95% CI	Risk of Bias A	B	C	D	E	F
**4.5.1 Behavioural programmes**														
a. Al–Jiffri 2013	−1.4	1.6	50	0.1	0.6	50	5.6%	−1.23 [−1.66, −0.80]	?	?			+	
b. Promrat 2010	−2	3	20	0.1	2.3	10	5.5%	−0.73 [−1.52, 0.05]	+	+		+	+	
c. AbdEl–Kader 2016	−2	0.7	50	0.3	0.5	50	5.6%	−3.75 [−4.41, −3.09]	?	+			?	
d. Sun 2012	−1.5	0.3	674	0.5	0.3	332	5.6%	−6.66 [−6.98, −6.34]	+	?		+	+	
f. Abenavoli 2017	0.2	0.5	20	0.6	0.3	10	5.5%	−0.87 [−1.67, −0.08]	−	?		+	+	
i. Asghari 2018	−0.2	0.5	30	0.7	1.9	30	5.6%	−0.64 [−1.16, −0.12]	+	−	+	+	+	
l. Katsagoni 2018 (D)	−0.5	0.8	21	−0.3	0.6	11	5.5%	−0.26 [−1.00, 0.47]	+	+		+	−	
n. StGeorge 2009 (M)	−0.2	2.3	36	0	1.5	17	5.6%	−0.09 [−0.67, 0.48]	?	?			+	−
o. Katsagoni 2018 (D+E)	−0.8	0.5	21	−0.3	0.6	10	5.5%	−0.91 [−1.70, −0.12]	+	+		+	−	
r. Cheng 2017 (D+E)	−0.1	0.5	29	0.2	0.6	15	5.6%	−0.55 [−1.19, 0.08]	+	+		+	+	−
t. StGeorge 2009 (L)	−0.4	1	73	0	1.5	17	5.6%	−0.36 [−0.89, 0.17]	?	?			+	−
u. Cheng 2017 (D)	0.2	0.5	28	0.2	0.6	14	5.6%	0.00 [−0.64, 0.64]	+	+		+	+	−
w. Eckard 2013 (MF)	−0.008	0.019	9	0	0.03	5	5.4%	−0.32 [−1.42, 0.78]	+	?		+	+	
y. Eckard 2013 (LF)	0.02	0.02	12	0	0.03	6	5.4%	0.81 [−0.22, 1.83]	+	?		+	+	
**Subtotal (95% CI)**			**1073**			**577**	**77.7%**	**−1.12 [−2.59, 0.35]**						
Heterogeneity: $Tau^2 = 7.73$; $Chi^2 = 1096.63$, df = 13 (P < 0.00001); $I^2 = 99\%$														
Test for overall effect: Z = 1.50 (P = 0.13)														
**4.5.2 Pharmacotherapy**														
h. Armstrong 2016	−1.8	3.7	23	0.7	9.5	22	5.6%	−0.34 [−0.93, 0.25]	+	+	+	+	+	
p. Zelber–Sagi 2004	−6.2	19.6	21	−0.8	3	23	5.6%	−0.39 [−0.98, 0.21]	−	−	+	+	+	
q. Harrison 2007	0.01	0.014	23	0.01	0.007	18	5.6%	0.00 [−0.62, 0.62]	?	−	−	−	+	−
x. Khoo 2017	−2.9	2.3	12	−3.2	2.6	12	5.5%	0.12 [−0.68, 0.92]	+	+	−	+	+	
**Subtotal (95% CI)**			**79**			**75**	**22.3%**	**−0.19 [−0.51, 0.13]**						
Heterogeneity: $Tau^2 = 0.00$; $Chi^2 = 1.61$, df = 3 (P = 0.66); $I^2 = 0\%$														
Test for overall effect: Z = 1.18 (P = 0.24)														
**Total (95% CI)**			**1152**			**652**	**100.0%**	**−0.91 [−2.11, 0.30]**						
Heterogeneity: $Tau^2 = 6.70$; $Chi^2 = 1219.38$, df = 17 (P < 0.00001); $I^2 = 99\%$														
Test for overall effect: Z = 1.47 (P = 0.14)														
Test for subgroup differences: $Chi^2 = 1.47$, df = 1 (P = 0.23), $I^2 = 32.0\%$														

Std. Mean Difference IV, Random, 95% CI

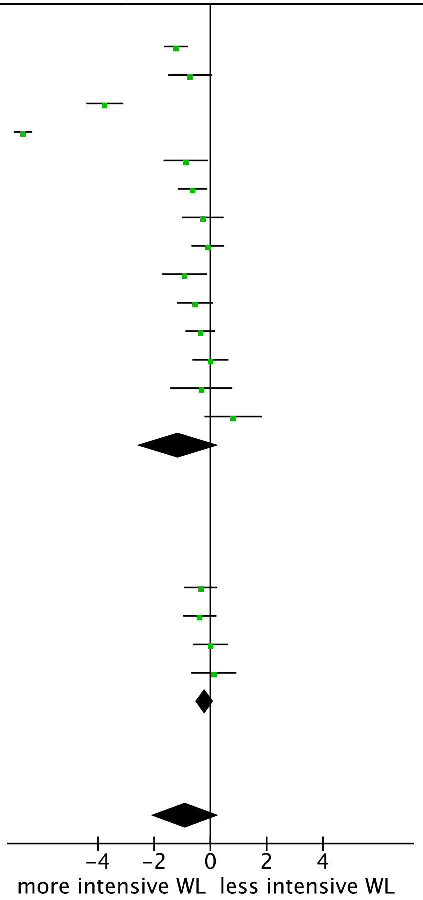

© 2019 Koutoukidis DA et al. *JAMA Internal Medicine*.

eFigure 5.4 Insulin (pmol/L) (subgroup analysis by the type of intervention)

Study or Subgroup	more intensive WL Mean	more intensive WL SD	more intensive WL Total	less intensive WL Mean	less intensive WL SD	less intensive WL Total	Weight	Mean Difference IV, Random, 95% CI	Risk of Bias A	B	C	D	E	F
**4.25.1 Behavioural programmes**														
b. Promrat 2010	-31.2	58.8	20	18	30.6	10	5.8%	-49.20 [-81.20, -17.20]	+	+		+	+	
d. Sun 2012	-27	31.8	674	22.2	25.2	332	8.2%	-49.20 [-52.82, -45.58]	+	?		+	+	
f. Abenavoli 2017	4.8	10.8	20	10.2	9	10	8.1%	-5.40 [-12.72, 1.92]	-	?		+	+	
i. Asghari 2018	-7.8	12	30	16.8	52.8	30	7.2%	-24.60 [-43.98, -5.22]	+	-	+	+	+	
l. Katsagoni 2018 (D)	-30.8	16.6	21	-6.5	14	11	7.9%	-24.30 [-35.20, -13.40]	+	+		+	-	
n. StGeorge 2009 (M)	4.2	25.2	36	-1.8	27	17	7.5%	6.00 [-9.25, 21.25]	?	?			+	-
o. Katsagoni 2018 (D+E)	-14.3	18.7	21	-6.5	14	10	7.8%	-7.80 [-19.60, 4.00]	+	+		+	-	
r. Cheng 2017 (D+E)	-4.2	24	29	0.6	35.4	15	7.1%	-4.80 [-24.73, 15.13]	+	+		+	+	-
t. StGeorge 2009 (L)	-9	22.2	73	-1.8	27	17	7.7%	-7.20 [-21.01, 6.61]	?	?			+	-
u. Cheng 2017 (D)	-0.6	19.2	28	0.6	35.4	14	7.1%	-1.20 [-21.06, 18.66]	+	+		+	+	-
w. Eckard 2013 (MF)	15.6	36	9	-6.6	41.4	5	4.7%	22.20 [-21.04, 65.44]	+	?		+	+	
y. Eckard 2013 (LF)	-31.2	44.4	12	-6.6	41.4	6	4.9%	-24.60 [-66.17, 16.97]	+	?		+	+	
**Subtotal (95% CI)**			**973**			**477**	**84.0%**	**-14.65 [-30.39, 1.09]**						
Heterogeneity: $Tau^2 = 658.12$; $Chi^2 = 215.71$, df = 11 (P < 0.00001); $I^2 = 95\%$														
Test for overall effect: Z = 1.82 (P = 0.07)														
**4.25.2 Pharmacotherapy**														
h. Armstrong 2016	-15.9	54.7	23	-34.7	164.1	22	2.7%	18.80 [-53.32, 90.92]	+	+	+	+	+	
p. Zelber-Sagi 2004	-81	235.8	21	-18.6	74.4	23	1.5%	-62.40 [-167.74, 42.94]	-	-	+	+	+	
q. Harrison 2007	-24	69.6	23	-6	16.8	18	6.1%	-18.00 [-47.48, 11.48]	?	-	-	-	+	-
x. Khoo 2017	-54.6	45.6	12	-56.4	37.2	12	5.7%	1.80 [-31.50, 35.10]	+	+	-	+	+	
**Subtotal (95% CI)**			**79**			**75**	**16.0%**	**-9.03 [-29.73, 11.66]**						
Heterogeneity: $Tau^2 = 0.00$; $Chi^2 = 2.32$, df = 3 (P = 0.51); $I^2 = 0\%$														
Test for overall effect: Z = 0.86 (P = 0.39)														
**Total (95% CI)**			**1052**			**552**	**100.0%**	**-13.75 [-28.04, 0.53]**						
Heterogeneity: $Tau^2 = 643.40$; $Chi^2 = 222.99$, df = 15 (P < 0.00001); $I^2 = 93\%$														
Test for overall effect: Z = 1.89 (P = 0.06)														
Test for subgroup differences: $Chi^2 = 0.18$, df = 1 (P = 0.67), $I^2 = 0\%$														

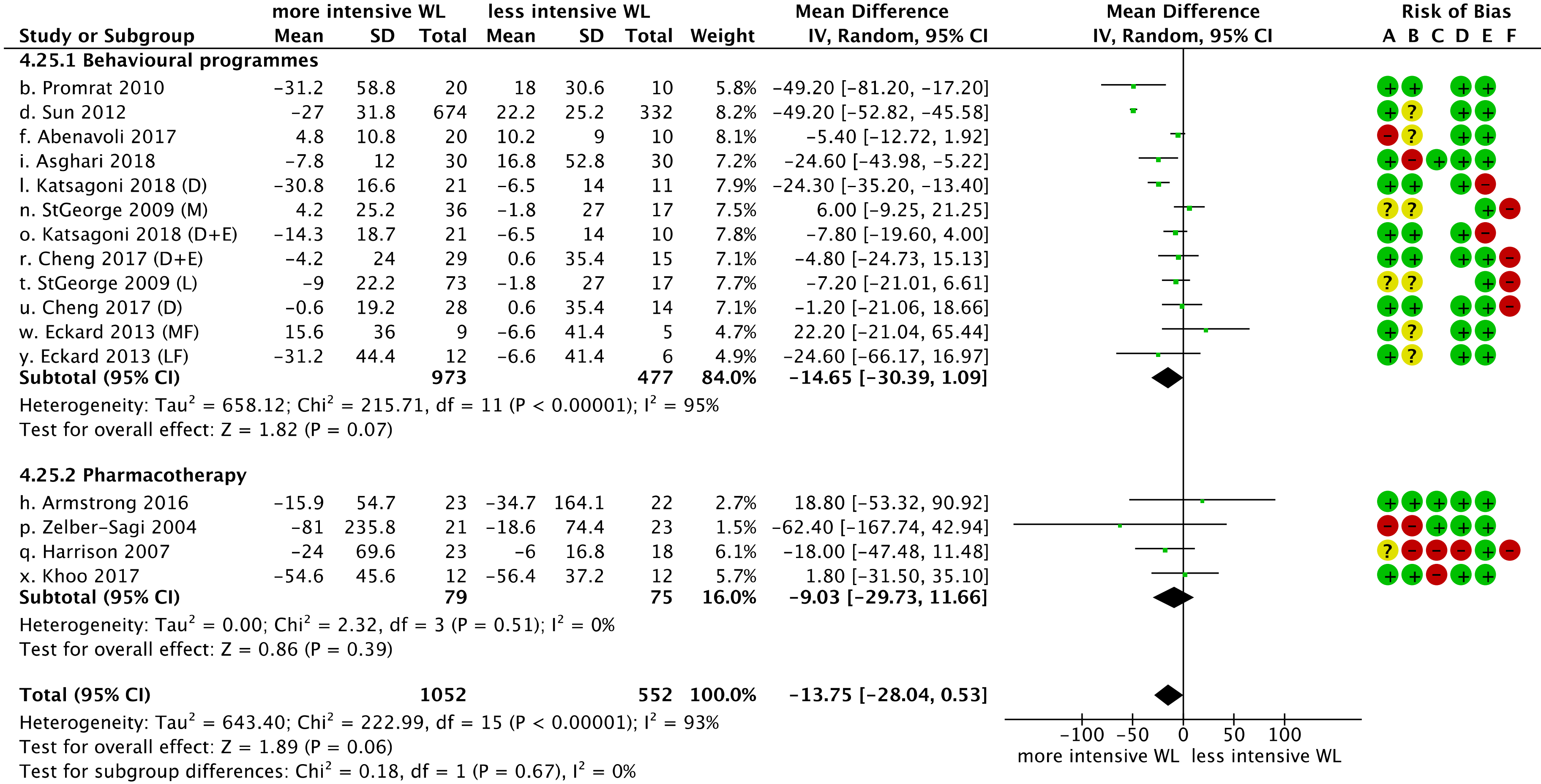

© 2019 Koutoukidis DA et al. *JAMA Internal Medicine*.

eFigure 5.5 ALT (U/L) (subgroup analysis by type of intervention)

Study or Subgroup	more intensive WL Mean	SD	Total	less intensive WL Mean	SD	Total	Weight	Mean Difference IV, Random, 95% CI	Risk of Bias A	B	C	D	E	F
**4.1.1 Behavioural programmes**														
a. Al-Jiffri 2013	-13.6	1.6	50	0.7	1.7	50	6.7%	-14.30 [-14.95, -13.65]	?	?			+	
b. Promrat 2010	-42.4	23	20	-16.5	14.2	10	3.3%	-25.90 [-39.28, -12.52]	+	+		+	+	
c. AbdEl-Kader 2016	-11.7	1.3	50	0.4	1.1	50	6.7%	-12.10 [-12.57, -11.63]	?	+			?	
d. Sun 2012	-22.9	9.2	674	3.7	9.2	332	6.6%	-26.60 [-27.81, -25.39]	+	?		+	+	
f. Abenavoli 2017	0.5	7.2	20	-0.3	8.9	10	5.4%	0.80 [-5.55, 7.15]	-	?		+	+	
g. Wong 2013	-17	17.7	77	-7	9.5	77	6.0%	-10.00 [-14.49, -5.51]	+	+			+	
i. Asghari 2018	-4.3	7.5	30	7.2	10.3	30	6.0%	-11.50 [-16.06, -6.94]	+	-	+	+	+	
j. Axley 2017	-12	8.1	8	-6	10.4	14	4.9%	-6.00 [-13.82, 1.82]	+	+			-	
k. Selezneva 2014	4	22	58	-21.3	11.4	116	5.5%	25.30 [19.27, 31.33]	?	?			+	
l. Katsagoni 2018 (D)	-20	26.1	21	-2.6	10.5	11	3.4%	-17.40 [-30.17, -4.63]	+	+		+	-	
m. Lim 2018	-35.3	39.3	43	-9.6	23.2	43	3.2%	-25.70 [-39.34, -12.06]	?	?			?	-
n. StGeorge 2009 (M)	-19.1	29.7	73	-7.3	18.5	17	3.9%	-11.80 [-22.92, -0.68]	?	?			+	-
o. Katsagoni 2018 (D+E)	-22.2	9.7	21	-2.6	10.5	10	4.9%	-19.60 [-27.32, -11.88]	+	+		+	-	
r. Cheng 2017 (D+E)	-1.5	4	29	1.5	3.3	15	6.5%	-3.00 [-5.22, -0.78]	+	+		+	+	-
s. Dong 2016	-4.7	8.1	130	-1.6	8.5	130	6.5%	-3.10 [-5.12, -1.08]	+	?		+	+	-
t. StGeorge 2009 (L)	-14.9	35.6	36	-7.3	18.5	17	3.0%	-7.60 [-22.18, 6.98]	?	?			+	-
u. Cheng 2017 (D)	-4.4	4	28	1.5	3.3	14	6.5%	-5.90 [-8.18, -3.62]	+	+		+	+	-
w. Eckard 2013 (MF)	-19.8	54.9	9	-4.3	38.7	5	0.4%	-15.50 [-64.87, 33.87]	+	?		+	+	
y. Eckard 2013 (LF)	-27.5	27.9	12	-4.3	38.7	6	0.8%	-23.20 [-57.96, 11.56]	+	?		+	+	
**Subtotal (95% CI)**			**1389**			**957**	**90.1%**	**-9.55 [-13.10, -6.01]**						
Heterogeneity: Tau^2 = 44.16; Chi^2 = 912.46, df = 18 (P < 0.00001); I^2 = 98%														
Test for overall effect: Z = 5.28 (P < 0.00001)														
**4.1.2 Pharmacotherapy**														
e. Bahmanabadi 2011	-7.5	10.9	20	-5.7	17.6	20	4.5%	-1.80 [-10.87, 7.27]	?	?	-	?	?	
h. Armstrong 2016	-26.6	34.4	23	-10.2	35.8	22	1.9%	-16.40 [-36.93, 4.13]	+	+	+	+	+	
p. Zelber-Sagi 2004	-30.6	59	21	-12.7	26.6	23	1.2%	-17.90 [-45.38, 9.58]	-	-	+	+	+	
q. Harrison 2007	-55	58.8	23	-45	32.4	18	1.2%	-10.00 [-38.31, 18.31]	?	-	-	-	+	-
x. Khoo 2017	-34	27	12	-42	46	12	1.0%	8.00 [-22.18, 38.18]	+	+	-	+	+	
**Subtotal (95% CI)**			**99**			**95**	**9.9%**	**-4.85 [-12.26, 2.57]**						
Heterogeneity: Tau^2 = 0.00; Chi^2 = 3.34, df = 4 (P = 0.50); I^2 = 0%														
Test for overall effect: Z = 1.28 (P = 0.20)														
**Total (95% CI)**			**1488**			**1052**	**100.0%**	**-9.26 [-12.63, -5.89]**						
Heterogeneity: Tau^2 = 44.10; Chi^2 = 920.58, df = 23 (P < 0.00001); I^2 = 98%														
Test for overall effect: Z = 5.39 (P < 0.00001)														
Test for subgroup differences: Chi^2 = 1.26, df = 1 (P = 0.26), I^2 = 20.6%														

Mean Difference IV, Random, 95% CI

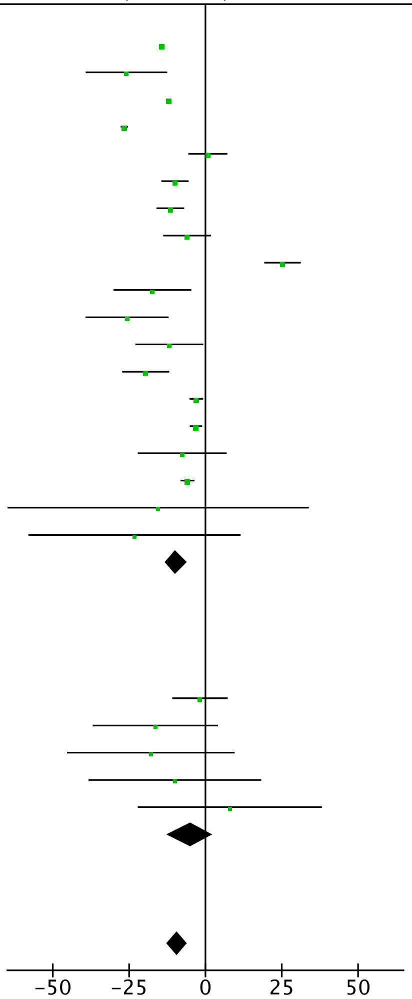

© 2019 Koutoukidis DA et al. *JAMA Internal Medicine*.

eFigure 5.6 Steatosis – Standardized Mean Difference as Assessed by Histologic Examination or MRI (Subgroup Analysis by the Type of Intervention)

Study or Subgroup	more intensive WL Mean	more intensive WL SD	more intensive WL Total	less intensive WL Mean	less intensive WL SD	less intensive WL Total	Weight	Std. Mean Difference IV, Random, 95% CI	Risk of Bias A	B	C	D	E	F
**4.3.1 Behavioural programmes**														
b. Promrat 2010	−1.1	0.4	18	−0.3	0.4	10	8.9%	−1.94 [−2.89, −0.99]	+	+		+	+	
f. Abenavoli 2017	−1	0.2	20	0.1	0.2	10	7.2%	−5.35 [−6.99, −3.71]	−	?		+	+	
g. Wong 2013	−6.8	3.1	77	−2.1	2.5	77	9.9%	−1.66 [−2.03, −1.29]	+	+			+	
i. Asghari 2018	−0.1	0.2	24	0	0.1	26	9.6%	−0.63 [−1.20, −0.06]	+	−	+	+	+	
r. Cheng 2017 (D+E)	−7.6	3.6	29	2.8	2.7	15	8.9%	−3.07 [−3.99, −2.15]	+	+		+	+	−
s. Dong 2016	−1.1	0.4	130	0	0.4	130	9.9%	−2.74 [−3.08, −2.40]	+	?		+	+	−
u. Cheng 2017 (D)	−5.4	3.3	28	2.8	2.7	14	9.0%	−2.58 [−3.45, −1.72]	+	+		+	+	−
**Subtotal (95% CI)**			**326**			**282**	**63.3%**	**−2.40 [−3.17, −1.62]**						
Heterogeneity: Tau^2 = 0.91; Chi^2 = 65.41, df = 6 (P < 0.00001); I^2 = 91%														
Test for overall effect: Z = 6.07 (P < 0.00001)														
**4.3.2 Pharmacotherapy**														
h. Armstrong 2016	−0.7	0.8	23	−0.4	0.8	22	9.6%	−0.37 [−0.96, 0.22]	+	+	+	+	+	
p. Zelber-Sagi 2004	0.1	0.3	11	−0.6	0.4	12	8.7%	1.90 [0.88, 2.91]	−	−	+	+	+	
q. Harrison 2007	0	0.2	23	0	0.2	18	9.5%	0.00 [−0.62, 0.62]	?	−	−	−	+	−
z. Ye 2017	−7.8	3.1	14	−1.9	2.4	16	8.9%	−2.09 [−3.00, −1.18]	?	?	?	?	?	−
**Subtotal (95% CI)**			**71**			**68**	**36.7%**	**−0.16 [−1.41, 1.10]**						
Heterogeneity: Tau^2 = 1.47; Chi^2 = 33.46, df = 3 (P < 0.00001); I^2 = 91%														
Test for overall effect: Z = 0.25 (P = 0.81)														
**Total (95% CI)**			**397**			**350**	**100.0%**	**−1.61 [−2.43, −0.80]**						
Heterogeneity: Tau^2 = 1.72; Chi^2 = 178.92, df = 10 (P < 0.00001); I^2 = 94%														
Test for overall effect: Z = 3.88 (P = 0.0001)														
Test for subgroup differences: Chi^2 = 8.88, df = 1 (P = 0.003), I^2 = 88.7%														

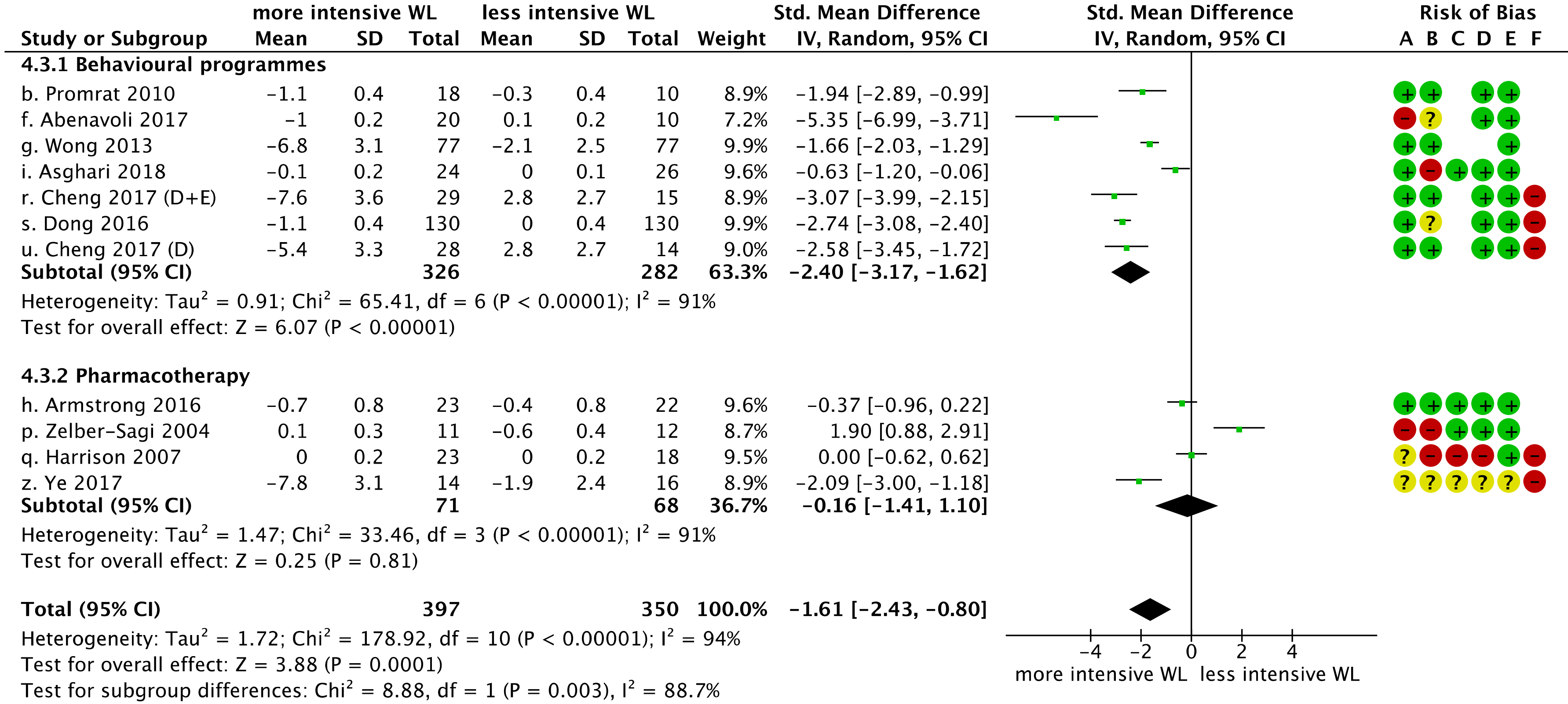

© 2019 Koutoukidis DA et al. *JAMA Internal Medicine*.

eFigure 5.7 AST (U/L) (subgroup analysis by type of intervention)

Study or Subgroup	more intensive WL Mean	SD	Total	less intensive WL Mean	SD	Total	Weight	Mean Difference IV, Random, 95% CI	Risk of Bias A	B	C	D	E	F
**5.3.1 Behavioural programmes**														
a. Al-Jiffri 2013	-11.6	2.2	50	0.7	1.8	50	7.2%	-12.30 [-13.09, -11.51]	?	?			+	
b. Promrat 2010	-20.2	14.1	20	-18	30.8	10	1.3%	-2.20 [-22.26, 17.86]	+	+		+	+	
c. AbdEl-Kader 2016	-9.5	1.6	50	0.4	1.4	50	7.2%	-9.90 [-10.49, -9.31]	?	+			?	
d. Sun 2012	-5.7	15.2	674	-0.5	5.7	332	7.1%	-5.20 [-6.50, -3.90]	+	?		+	+	
f. Abenavoli 2017	2.8	6.3	20	12.4	15.2	10	3.4%	-9.60 [-19.42, 0.22]	-	?		+	+	
g. Wong 2013	-4	6.2	77	-3	5.4	77	7.0%	-1.00 [-2.84, 0.84]	+	+			+	
i. Asghari 2018	-4.1	3.6	30	4.4	11.8	30	5.9%	-8.50 [-12.91, -4.09]	+	-	+	+	+	
j. Axley 2017	-9	2.2	8	0	2	14	7.0%	-9.00 [-10.85, -7.15]	+	+			-	
k. Selezneva 2014	0.1	13.7	58	-11	16.4	116	5.8%	11.10 [6.48, 15.72]	?	?			+	
m. Lim 2018	-19.3	26.1	43	-5.1	13.7	43	3.8%	-14.20 [-23.01, -5.39]	?	?			?	-
n. StGeorge 2009 (M)	-6.9	18.3	36	-2.6	14.7	17	3.6%	-4.30 [-13.50, 4.90]	?	?			+	-
r. Cheng 2017 (D+E)	-4.9	2.4	29	4	2.3	14	7.1%	-8.90 [-10.39, -7.41]	+	+		+	+	-
s. Dong 2016	-1	3.5	130	0.4	4.9	130	7.2%	-1.40 [-2.44, -0.36]	+	?		+	+	-
t. StGeorge 2009 (L)	-8.3	16.1	73	-2.6	14.7	17	4.2%	-5.70 [-13.60, 2.20]	?	?			+	-
u. Cheng 2017 (D)	0.4	2.6	28	4	2.3	15	7.1%	-3.60 [-5.11, -2.09]	+	+		+	+	-
w. Eckard 2013 (MF)	-19.6	47.9	9	-2.9	25.8	5	0.4%	-16.70 [-55.31, 21.91]	+	?		+	+	
y. Eckard 2013 (LF)	-15.9	19.1	12	-2.9	25.8	6	1.0%	-13.00 [-36.30, 10.30]	+	?		+	+	
**Subtotal (95% CI)**			**1347**			**936**	**86.1%**	**-5.79 [-8.38, -3.19]**						
Heterogeneity: $Tau^2 = 20.54$; $Chi^2 = 488.52$, df = 16 (P < 0.00001); $I^2 = 97\%$														
Test for overall effect: Z = 4.38 (P < 0.0001)														
**5.3.2 Pharmacotherapy**														
e. Bahmanabadi 2011	4.4	6.7	20	-1.7	7.9	20	5.8%	6.10 [1.56, 10.64]	?	?	-	?	?	
h. Armstrong 2016	-15.8	21.8	23	-8.6	28.3	22	2.0%	-7.20 [-22.01, 7.61]	+	+	+	+	+	
p. Zelber-Sagi 2004	-18.9	33	21	-8.8	17.2	23	1.8%	-10.10 [-25.87, 5.67]	-	-	+	+	+	
q. Harrison 2007	-28	23.2	23	-29	18.4	18	2.5%	1.00 [-11.73, 13.73]	?	-	-	-	+	-
x. Khoo 2017	-18	15	12	-23	24	12	1.8%	5.00 [-11.01, 21.01]	+	+	-	+	+	
**Subtotal (95% CI)**			**99**			**95**	**13.9%**	**1.14 [-5.28, 7.56]**						
Heterogeneity: $Tau^2 = 19.42$; $Chi^2 = 6.30$, df = 4 (P = 0.18); $I^2 = 36\%$														
Test for overall effect: Z = 0.35 (P = 0.73)														
**Total (95% CI)**			**1446**			**1031**	**100.0%**	**-4.84 [-7.31, -2.38]**						
Heterogeneity: $Tau^2 = 21.83$; $Chi^2 = 530.14$, df = 21 (P < 0.00001); $I^2 = 96\%$														
Test for overall effect: Z = 3.85 (P = 0.0001)														
Test for subgroup differences: $Chi^2 = 3.84$, df = 1 (P = 0.05), $I^2 = 74.0\%$														

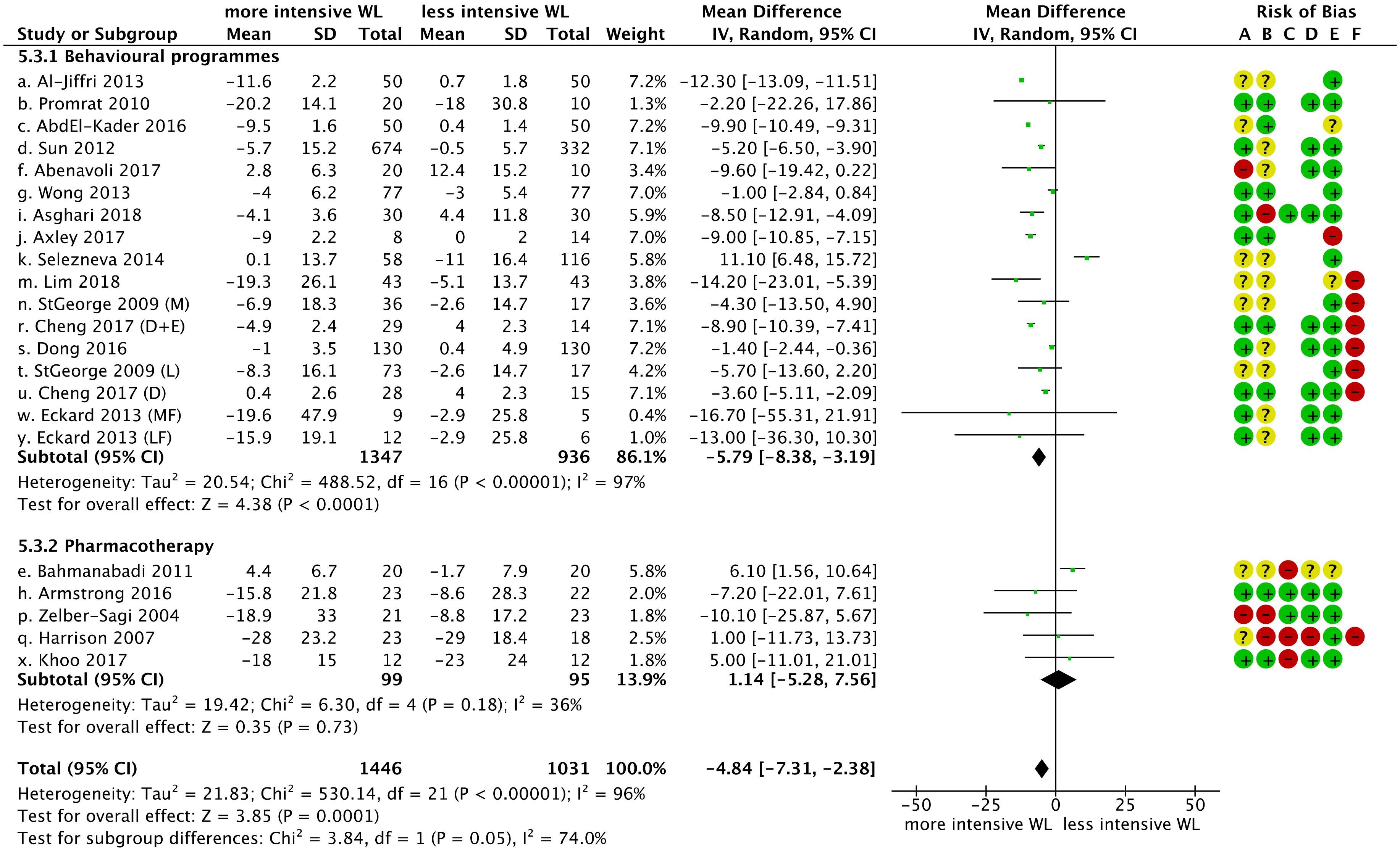

© 2019 Koutoukidis DA et al. *JAMA Internal Medicine*.

6 Subgroup analysis by the intensity of the comparator intervention

eFigure 6.1 Weight change (kg) by the intensity of the comparator intervention

	more intensive WL			less intensive WL				Mean Difference	Risk of Bias					
**Study or Subgroup**	**Mean**	**SD**	**Total**	**Mean**	**SD**	**Total**	**Weight**	**IV, Random, 95% CI**	**A**	**B**	**C**	**D**	**E**	**F**
**5.15.1 Comparator: usual care/minimal intervention**														
a. Al-Jiffri 2013	-13.7	3.4	50	0.8	3	50	4.5%	-14.50 [-15.76, -13.24]	?	?			+	
b. Promrat 2010	-8.7	12.4	20	-0.5	7.4	10	2.3%	-8.20 [-15.31, -1.09]	+	+		+	+	
c. AbdEl-Kader 2016	-5.9	2.2	50	2.1	2.9	50	4.6%	-8.00 [-9.01, -6.99]	?	+			?	
d. Sun 2012	-6.1	9.2	674	1.3	9.2	332	4.5%	-7.40 [-8.61, -6.19]	+	?		+	+	
f. Abenavoli 2017	-5.1	3.4	20	0.4	2.3	10	4.3%	-5.50 [-7.56, -3.44]	−	?		+	+	
g. Wong 2013	-5.6	5.7	77	-0.6	3.7	77	4.5%	-5.00 [-6.52, -3.48]	+	+			+	
h. Armstrong 2016	-5.3	4.7	23	-0.6	4.4	22	4.1%	-4.70 [-7.36, -2.04]	+	+	+	+	+	
i. Asghari 2018	-4.1	4	30	0	2.7	30	4.4%	-4.10 [-5.83, -2.37]	+	−	+	+	+	
j. Axley 2017	-2.7	5	8	0.9	6	14	3.2%	-3.60 [-8.28, 1.08]	+	+			−	
l. Katsagoni 2018 (D)	-5.6	4.1	21	-2.8	3.9	11	4.0%	-2.80 [-5.70, 0.10]	+	+		+	−	
n. StGeorge 2009 (M)	-2.8	3.4	73	-0.5	3.7	17	4.3%	-2.30 [-4.22, -0.38]	?	?			+	−
o. Katsagoni 2018 (D+E)	-4.9	4.1	21	-2.8	3.9	10	3.9%	-2.10 [-5.09, 0.89]	+	+		+	−	
r. Cheng 2017 (D+E)	-1.5	3.2	29	0.2	3.3	15	4.3%	-1.70 [-3.74, 0.34]	+	+		+	+	−
s. Dong 2016	-1.4	4.9	130	0.1	4.1	130	4.6%	-1.50 [-2.60, -0.40]	+	?		+	+	−
t. StGeorge 2009 (L)	-1.9	3.7	36	-0.5	3.7	17	4.3%	-1.40 [-3.53, 0.73]	?	?			+	−
u. Cheng 2017 (D)	-0.8	3.1	28	0.2	3.3	14	4.3%	-1.00 [-3.08, 1.08]	+	+		+	+	−
w. Eckard 2013 (MF)	-3	4.7	9	-2.5	5.3	5	2.8%	-0.50 [-6.07, 5.07]	+	?		+	+	
y. Eckard 2013 (LF)	-0.2	5.4	12	-2.5	5.3	6	3.0%	2.30 [-2.93, 7.53]	+	?		+	+	
**Subtotal (95% CI)**			**1311**			**820**	**71.7%**	**-4.10 [-6.17, -2.03]**						
Heterogeneity: $Tau^2 = 17.80$; $Chi^2 = 361.58$, df = 17 (P < 0.00001); $I^2 = 95\%$														
Test for overall effect: Z = 3.88 (P = 0.0001)														
**5.15.2 Comparator: lower intensity intervention**														
e. Bahmanabadi 2011	-13	10	20	-6.1	3	20	3.2%	-6.90 [-11.48, -2.32]	?	?	−	?	?	
k. Selezneva 2014	-9.3	1.8	58	-6.2	1.7	116	4.6%	-3.10 [-3.66, -2.54]	?	?			+	
m. Lim 2018	-3.2	2.5	43	-0.9	2	43	4.6%	-2.30 [-3.26, -1.34]	?	?			?	−
p. Zelber-Sagi 2004	-7.7	6.8	21	-5.9	5.9	23	3.6%	-1.80 [-5.58, 1.98]	−	−	+	+	+	
q. Harrison 2007	-8.2	5	23	-6.4	5.2	18	3.9%	-1.80 [-4.95, 1.35]	?	−	−	−	+	−
v. Lee 2012	-1.6	3.1	8	-0.8	2.3	10	4.1%	-0.80 [-3.38, 1.78]	+	?	+	?	+	
x. Khoo 2017	-3.5	2.1	12	-3.5	3.3	12	4.2%	0.00 [-2.21, 2.21]	+	+	−	+	+	
**Subtotal (95% CI)**			**185**			**242**	**28.3%**	**-2.21 [-3.25, -1.16]**						
Heterogeneity: $Tau^2 = 0.86$; $Chi^2 = 14.22$, df = 6 (P = 0.03); $I^2 = 58\%$														
Test for overall effect: Z = 4.13 (P < 0.0001)														
**Total (95% CI)**			**1496**			**1062**	**100.0%**	**-3.61 [-5.11, -2.12]**						
Heterogeneity: $Tau^2 = 12.45$; $Chi^2 = 458.04$, df = 24 (P < 0.00001); $I^2 = 95\%$														
Test for overall effect: Z = 4.74 (P < 0.00001)														
Test for subgroup differences: $Chi^2 = 2.56$, df = 1 (P = 0.11), $I^2 = 61.0\%$														

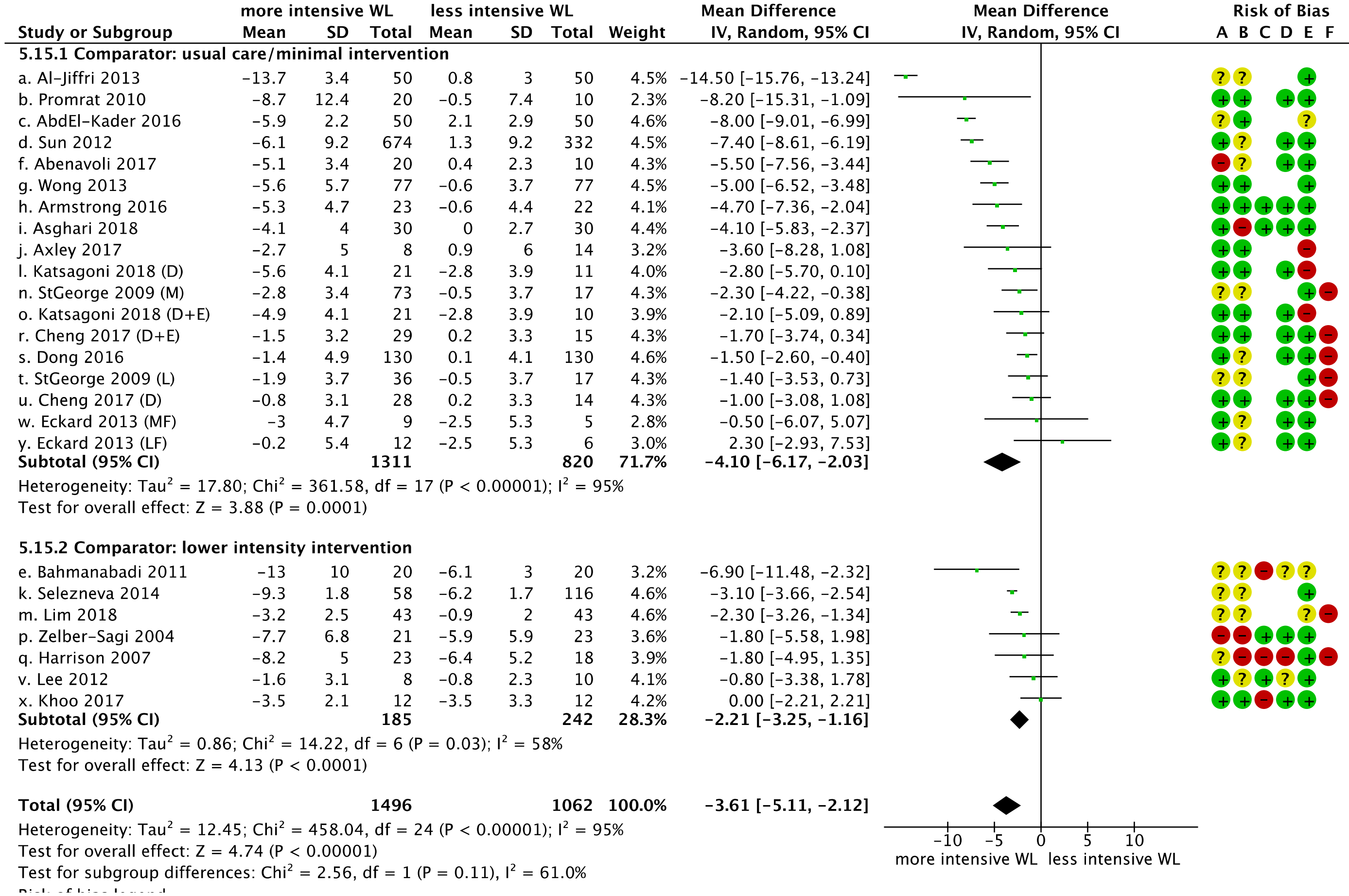

[illegible]

© 2019 Koutoukidis DA et al. *JAMA Internal Medicine*.

eFigure 6.2 Glucose (mmol/L) by the intensity of the comparator intervention

Study or Subgroup	more intensive WL Mean	more intensive WL SD	more intensive WL Total	less intensive WL Mean	less intensive WL SD	less intensive WL Total	Weight	Mean Difference IV, Random, 95% CI	Risk of Bias A	B	C	D	E	F
**5.13.1 Comparator: usual care/minimal intervention**														
b. Promrat 2010	−0.6	0.8	20	−1.7	3	10	1.2%	1.10 [−0.79, 2.99]	+	+		+	+	
d. Sun 2012	−1.6	0.7	674	−0.2	1	332	6.9%	−1.40 [−1.52, −1.28]	+	?		+	+	
f. Abenavoli 2017	−0.2	0.2	20	0	0.1	10	6.9%	−0.20 [−0.31, −0.09]	−	?		+	+	
g. Wong 2013	0	0.6	77	0.1	0.6	77	6.7%	−0.10 [−0.29, 0.09]	+	+			+	
h. Armstrong 2016	−1	1.5	23	0.7	2.3	23	2.6%	−1.70 [−2.82, −0.58]	+	+	+	+	+	
i. Asghari 2018	0.2	0.2	30	0.1	0.1	30	7.0%	0.10 [0.02, 0.18]	+	−	+	+	+	
l. Katsagoni 2018 (D)	0	0.2	21	0.1	0.1	11	6.9%	−0.10 [−0.20, 0.00]	+	+		+	−	
n. StGeorge 2009 (M)	−0.2	0.7	73	0	0.7	17	5.9%	−0.20 [−0.57, 0.17]	?	?			+	−
o. Katsagoni 2018 (D+E)	−0.1	0.1	21	0.1	0.1	10	7.0%	−0.20 [−0.28, −0.12]	+	+		+	−	
q. Harrison 2007	−0.4	0.2	23	−0.7	1.8	18	3.6%	0.30 [−0.54, 1.14]	?	−	−	−	+	−
r. Cheng 2017 (D+E)	0.1	0.2	29	0.2	0.2	15	6.9%	−0.10 [−0.22, 0.02]	+	+		+	+	−
s. Dong 2016	0	0.5	130	0.2	0.5	130	6.9%	−0.20 [−0.32, −0.08]	+	?		+	+	−
t. StGeorge 2009 (L)	−0.3	0.6	36	0	0.7	17	5.9%	−0.30 [−0.69, 0.09]	?	?			+	−
u. Cheng 2017 (D)	0.3	0.2	28	0.2	0.2	14	6.9%	0.10 [−0.03, 0.23]	+	+		+	+	−
w. Eckard 2013 (MF)	−0.1	1.1	9	0.1	0.5	5	3.6%	−0.20 [−1.04, 0.64]	+	?		+	+	
y. Eckard 2013 (LF)	−0.5	1.3	12	0.1	0.5	6	3.6%	−0.60 [−1.44, 0.24]	+	?		+	+	
**Subtotal (95% CI)**			**1226**			**725**	**88.7%**	**−0.25 [−0.48, −0.02]**						
Heterogeneity: $Tau^2 = 0.17$; $Chi^2 = 478.40$, df = 15 (P < 0.00001); $I^2 = 97\%$														
Test for overall effect: Z = 2.15 (P = 0.03)														
**5.13.2 Comparator: lower intensity intervention**														
p. Zelber-Sagi 2004	−7.1	0.9	21	−5.1	0.3	23	5.8%	−2.00 [−2.40, −1.60]	−	−	+	+	+	
x. Khoo 2017	−0.7	0.6	12	−0.6	0.5	12	5.6%	−0.10 [−0.54, 0.34]	+	+	−	+	+	
**Subtotal (95% CI)**			**33**			**35**	**11.3%**	**−1.05 [−2.91, 0.81]**						
Heterogeneity: $Tau^2 = 1.76$; $Chi^2 = 38.69$, df = 1 (P < 0.00001); $I^2 = 97\%$														
Test for overall effect: Z = 1.11 (P = 0.27)														
**Total (95% CI)**			**1259**			**760**	**100.0%**	**−0.34 [−0.57, −0.12]**						
Heterogeneity: $Tau^2 = 0.19$; $Chi^2 = 554.22$, df = 17 (P < 0.00001); $I^2 = 97\%$														
Test for overall effect: Z = 2.96 (P = 0.003)														
Test for subgroup differences: $Chi^2 = 0.70$, df = 1 (P = 0.40), $I^2 = 0\%$														

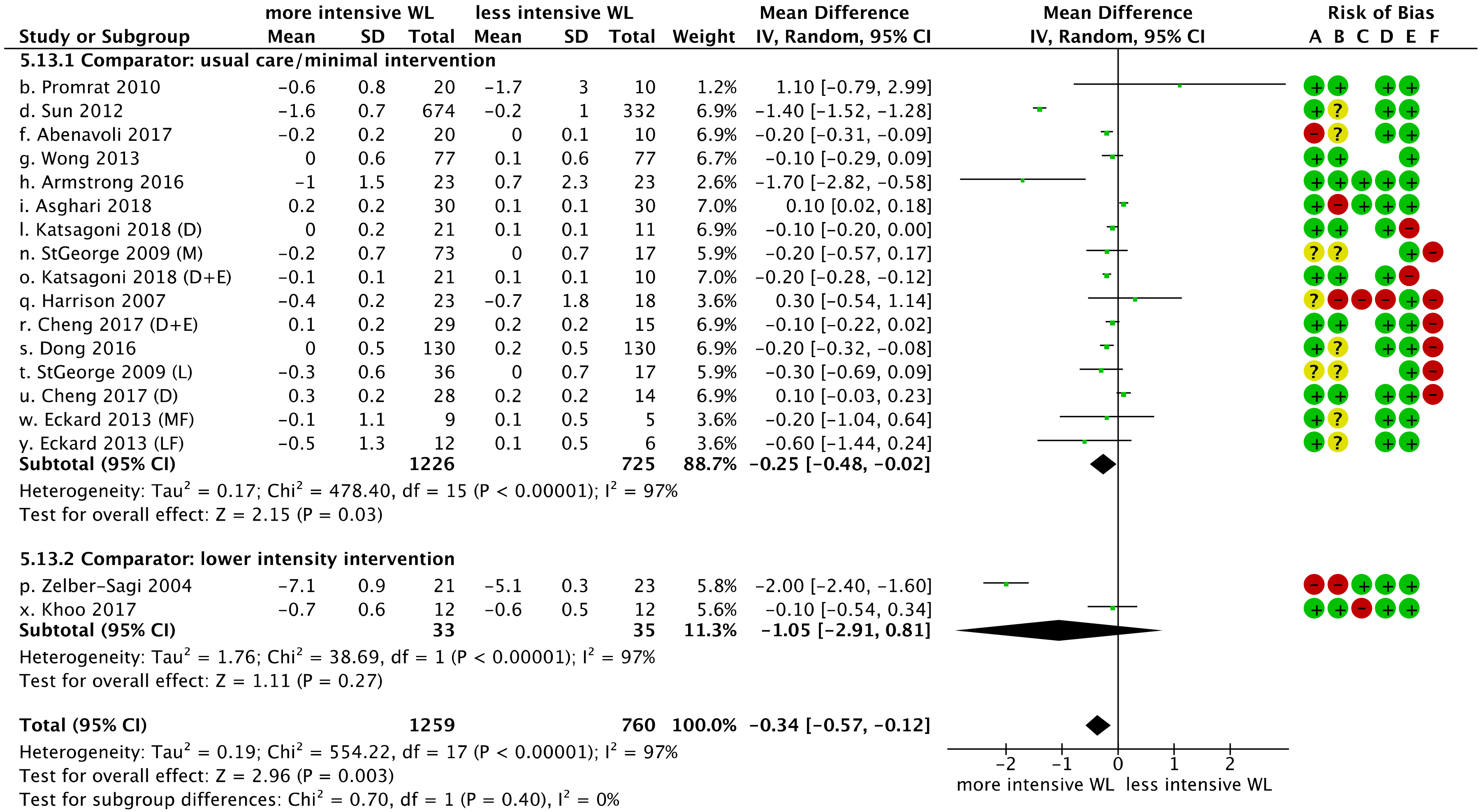

© 2019 Koutoukidis DA et al. *JAMA Internal Medicine*.

eFigure 6.3 Insulin resistance indices by the intensity of the comparator intervention

Study or Subgroup	more intensive WL Mean	SD	Total	less intensive WL Mean	SD	Total	Weight	Std. Mean Difference IV, Random, 95% CI
**5.14.1 Comparator: usual care/minimal intervention**								
a. Al-Jiffri 2013	-1.4	1.6	50	0.1	0.6	50	5.6%	-1.23 [-1.66, -0.80]
b. Promrat 2010	-2	3	20	0.1	2.3	10	5.5%	-0.73 [-1.52, 0.05]
c. AbdEl-Kader 2016	-2	0.7	50	0.3	0.5	50	5.6%	-3.75 [-4.41, -3.09]
d. Sun 2012	-1.5	0.3	674	0.5	0.3	332	5.6%	-6.66 [-6.98, -6.34]
f. Abenavoli 2017	0.2	0.5	20	0.6	0.3	10	5.5%	-0.87 [-1.67, -0.08]
h. Armstrong 2016	-1.8	3.7	23	0.7	9.5	22	5.6%	-0.34 [-0.93, 0.25]
i. Asghari 2018	-0.2	0.5	30	0.7	1.9	30	5.6%	-0.64 [-1.16, -0.12]
l. Katsagoni 2018 (D)	-0.5	0.8	21	-0.3	0.6	11	5.5%	-0.26 [-1.00, 0.47]
n. StGeorge 2009 (M)	-0.2	2.3	36	0	1.5	17	5.6%	-0.09 [-0.67, 0.48]
o. Katsagoni 2018 (D+E)	-0.8	0.5	21	-0.3	0.6	10	5.5%	-0.91 [-1.70, -0.12]
r. Cheng 2017 (D+E)	-0.1	0.5	29	0.2	0.6	15	5.6%	-0.55 [-1.19, 0.08]
t. StGeorge 2009 (L)	-0.4	1	73	0	1.5	17	5.6%	-0.36 [-0.89, 0.17]
u. Cheng 2017 (D)	0.2	0.5	28	0.2	0.6	14	5.6%	0.00 [-0.64, 0.64]
w. Eckard 2013 (MF)	-0.008	0.019	9	0	0.03	5	5.4%	-0.32 [-1.42, 0.78]
y. Eckard 2013 (LF)	0.02	0.02	12	0	0.03	6	5.4%	0.81 [-0.22, 1.83]
**Subtotal (95% CI)**			**1096**			**599**	**83.3%**	**-1.07 [-2.46, 0.32]**
Heterogeneity: $Tau^2 = 7.37$; $Chi^2 = 1131.59$, df = 14 ($P < 0.00001$); $I^2 = 99\%$								
Test for overall effect: Z = 1.51 (P = 0.13)								
**5.14.2 Comparator: lower intensity intervention**								
p. Zelber-Sagi 2004	-6.2	19.6	21	-0.8	3	23	5.6%	-0.39 [-0.98, 0.21]
q. Harrison 2007	0.01	0.014	23	0.01	0.007	18	5.6%	0.00 [-0.62, 0.62]
x. Khoo 2017	-2.9	2.3	12	-3.2	2.6	12	5.5%	0.12 [-0.68, 0.92]
**Subtotal (95% CI)**			**56**			**53**	**16.7%**	**-0.13 [-0.51, 0.25]**
Heterogeneity: $Tau^2 = 0.00$; $Chi^2 = 1.25$, df = 2 (P = 0.54); $I^2 = 0\%$								
Test for overall effect: Z = 0.67 (P = 0.50)								
**Total (95% CI)**			**1152**			**652**	**100.0%**	**-0.91 [-2.11, 0.30]**
Heterogeneity: $Tau^2 = 6.70$; $Chi^2 = 1219.38$, df = 17 ($P < 0.00001$); $I^2 = 99\%$								
Test for overall effect: Z = 1.47 (P = 0.14)								
Test for subgroup differences: $Chi^2 = 1.65$, df = 1 (P = 0.20), $I^2 = 39.3\%$								

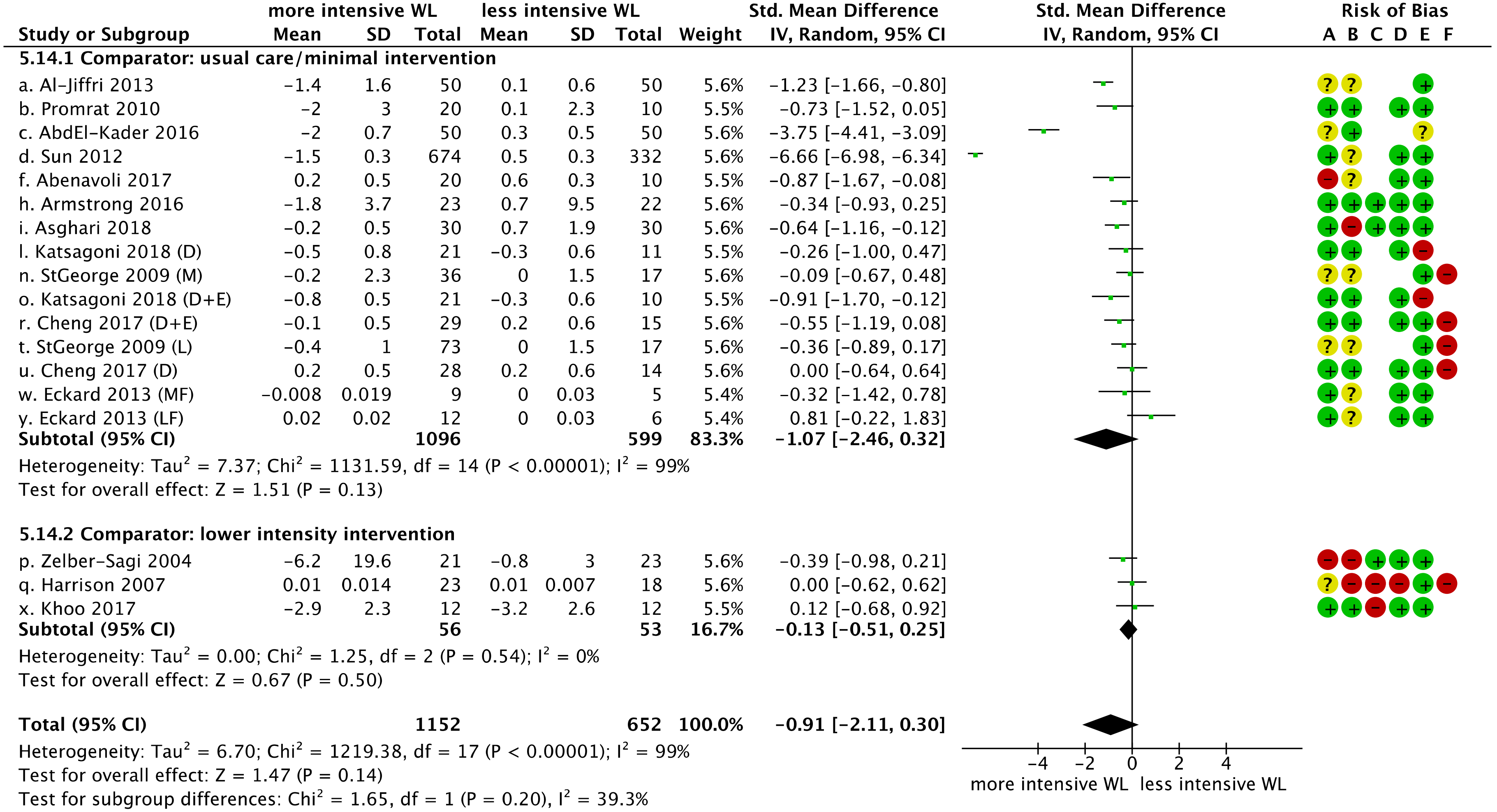

© 2019 Koutoukidis DA et al. *JAMA Internal Medicine*.

eFigure 6.4 Insulin (pmol/L) by the intensity of the comparator intervention

Study or Subgroup	more intensive WL Mean	SD	Total	less intensive WL Mean	SD	Total	Weight	Mean Difference IV, Random, 95% CI	Risk of Bias A	B	C	D	E	F
**5.25.1 Comparator: usual care/minimal intervention**														
b. Promrat 2010	−31.2	58.8	20	18	30.6	10	5.8%	−49.20 [−81.20, −17.20]	+	+		+	+	
d. Sun 2012	−27	31.8	674	22.2	25.2	332	8.2%	−49.20 [−52.82, −45.58]	+	?		+	+	
f. Abenavoli 2017	4.8	10.8	20	10.2	9	10	8.1%	−5.40 [−12.72, 1.92]	−	?		+	+	
h. Armstrong 2016	−15.9	54.7	23	−34.7	164.1	22	2.7%	18.80 [−53.32, 90.92]	+	+	+	+	+	
i. Asghari 2018	−7.8	12	30	16.8	52.8	30	7.2%	−24.60 [−43.98, −5.22]	+	−	+	+	+	
l. Katsagoni 2018 (D)	−30.8	16.6	21	−6.5	14	11	7.9%	−24.30 [−35.20, −13.40]	+	+		+	−	
n. StGeorge 2009 (M)	4.2	25.2	36	−1.8	27	17	7.5%	6.00 [−9.25, 21.25]	?	?			+	−
o. Katsagoni 2018 (D+E)	−14.3	18.7	21	−6.5	14	10	7.8%	−7.80 [−19.60, 4.00]	+	+		+	−	
r. Cheng 2017 (D+E)	−4.2	24	29	0.6	35.4	15	7.1%	−4.80 [−24.73, 15.13]	+	+		+	+	−
t. StGeorge 2009 (L)	−9	22.2	73	−1.8	27	17	7.7%	−7.20 [−21.01, 6.61]	?	?			+	−
u. Cheng 2017 (D)	−0.6	19.2	28	0.6	35.4	14	7.1%	−1.20 [−21.06, 18.66]	+	+		+	+	−
w. Eckard 2013 (MF)	15.6	36	9	−6.6	41.4	5	4.7%	22.20 [−21.04, 65.44]	+	?		+	+	
y. Eckard 2013 (LF)	−31.2	44.4	12	−6.6	41.4	6	4.9%	−24.60 [−66.17, 16.97]	+	?		+	+	
**Subtotal (95% CI)**			**996**			**499**	**86.7%**	**−13.61 [−29.12, 1.89]**						
Heterogeneity: $Tau^2 = 659.06$; $Chi^2 = 217.67$, df = 12 (P < 0.00001); $I^2 = 94\%$														
Test for overall effect: Z = 1.72 (P = 0.09)														
**5.25.2 Comparator: lower intensity intervention**														
p. Zelber-Sagi 2004	−81	235.8	21	−18.6	74.4	23	1.5%	−62.40 [−167.74, 42.94]	−	−	+	+	+	
q. Harrison 2007	−24	69.6	23	−6	16.8	18	6.1%	−18.00 [−47.48, 11.48]	?	−	−	−	+	−
x. Khoo 2017	−54.6	45.6	12	−56.4	37.2	12	5.7%	1.80 [−31.50, 35.10]	+	+	−	+	+	
**Subtotal (95% CI)**			**56**			**53**	**13.3%**	**−11.53 [−33.14, 10.07]**						
Heterogeneity: $Tau^2 = 0.00$; $Chi^2 = 1.70$, df = 2 (P = 0.43); $I^2 = 0\%$														
Test for overall effect: Z = 1.05 (P = 0.30)														
**Total (95% CI)**			**1052**			**552**	**100.0%**	**−13.75 [−28.04, 0.53]**						
Heterogeneity: $Tau^2 = 643.40$; $Chi^2 = 222.99$, df = 15 (P < 0.00001); $I^2 = 93\%$														
Test for overall effect: Z = 1.89 (P = 0.06)														
Test for subgroup differences: $Chi^2 = 0.02$, df = 1 (P = 0.88), $I^2 = 0\%$														

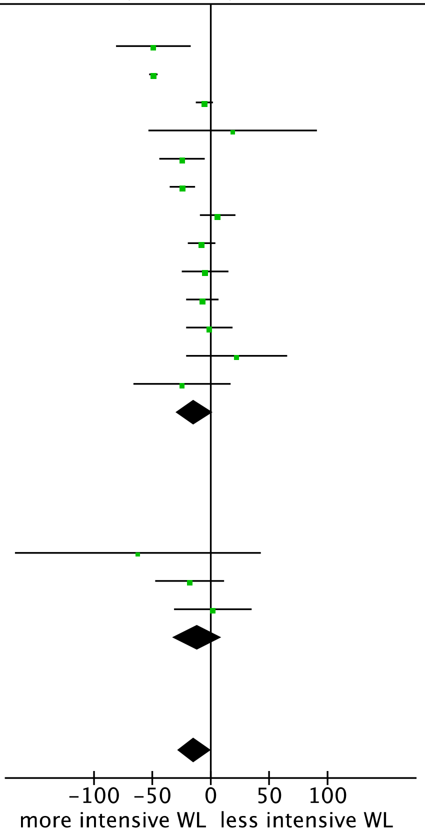

© 2019 Koutoukidis DA et al. *JAMA Internal Medicine*.

eFigure 6.5 ALT (U/L) by the intensity of the comparator intervention

Study or Subgroup	more intensive WL Mean	SD	Total	less intensive WL Mean	SD	Total	Weight	Mean Difference IV, Random, 95% CI
**5.1.1 Comparator: usual care/minimal intervention**								
a. Al-Jiffri 2013	-13.6	1.6	50	0.7	1.7	50	6.4%	-14.30 [-14.95, -13.65]
b. Promrat 2010	-42.4	23	20	-16.5	14.2	10	3.1%	-25.90 [-39.28, -12.52]
c. AbdEl-Kader 2016	-11.7	1.3	50	0.4	1.1	50	6.5%	-12.10 [-12.57, -11.63]
d. Sun 2012	-22.9	9.2	674	3.7	9.2	332	6.4%	-26.60 [-27.81, -25.39]
f. Abenavoli 2017	0.5	7.2	20	-0.3	8.9	10	5.2%	0.80 [-5.55, 7.15]
g. Wong 2013	-17	17.7	77	-7	9.5	77	5.8%	-10.00 [-14.49, -5.51]
h. Armstrong 2016	-26.6	34.4	23	-10.2	35.8	22	1.9%	-16.40 [-36.93, 4.13]
i. Asghari 2018	-4.3	7.5	30	7.2	10.3	30	5.8%	-11.50 [-16.06, -6.94]
j. Axley 2017	-12	8.1	8	-6	10.4	14	4.7%	-6.00 [-13.82, 1.82]
l. Katsagoni 2018 (D)	-20	26.1	21	-2.6	10.5	11	3.3%	-17.40 [-30.17, -4.63]
n. StGeorge 2009 (M)	-19.1	29.7	73	-7.3	18.5	17	3.7%	-11.80 [-22.92, -0.68]
o. Katsagoni 2018 (D+E)	-22.2	9.7	21	-2.6	10.5	10	4.8%	-19.60 [-27.32, -11.88]
r. Cheng 2017 (D+E)	-1.5	4	29	1.5	3.3	15	6.3%	-3.00 [-5.22, -0.78]
s. Dong 2016	-4.7	8.1	130	-1.6	8.5	130	6.3%	-3.10 [-5.12, -1.08]
t. StGeorge 2009 (L)	-14.9	35.6	36	-7.3	18.5	17	2.9%	-7.60 [-22.18, 6.98]
u. Cheng 2017 (D)	-4.4	4	28	1.5	3.3	14	6.3%	-5.90 [-8.18, -3.62]
w. Eckard 2013 (MF)	-19.8	54.9	9	-4.3	38.7	5	0.4%	-15.50 [-64.87, 33.87]
y. Eckard 2013 (LF)	-27.5	27.9	12	-4.3	38.7	6	0.8%	-23.20 [-57.96, 11.56]
**Subtotal (95% CI)**			**1311**			**820**	**80.5%**	**-11.32 [-14.74, -7.91]**
Heterogeneity: $Tau^2 = 36.62$; $Chi^2 = 752.85$, df = 17 (P < 0.00001); $I^2 = 98\%$								
Test for overall effect: Z = 6.50 (P < 0.00001)								
**5.1.2 Comparator: lower intensity intervention**								
e. Bahmanabadi 2011	-7.5	10.9	20	-5.7	17.6	20	4.3%	-1.80 [-10.87, 7.27]
k. Selezneva 2014	4	22	58	-21.3	11.4	116	5.3%	25.30 [19.27, 31.33]
m. Lim 2018	-35.3	39.3	43	-9.6	23.2	43	3.1%	-25.70 [-39.34, -12.06]
p. Zelber-Sagi 2004	-30.6	59	21	-12.7	26.6	23	1.2%	-17.90 [-45.38, 9.58]
q. Harrison 2007	-55	58.8	23	-45	32.4	18	1.1%	-10.00 [-38.31, 18.31]
v. Lee 2012	-53	13.3	8	-27.5	13.3	10	3.4%	-25.50 [-37.86, -13.14]
x. Khoo 2017	-34	27	12	-42	46	12	1.0%	8.00 [-22.18, 38.18]
**Subtotal (95% CI)**			**185**			**242**	**19.5%**	**-6.59 [-26.11, 12.93]**
Heterogeneity: $Tau^2 = 597.23$; $Chi^2 = 92.01$, df = 6 (P < 0.00001); $I^2 = 93\%$								
Test for overall effect: Z = 0.66 (P = 0.51)								
**Total (95% CI)**			**1496**			**1062**	**100.0%**	**-9.81 [-13.12, -6.50]**
Heterogeneity: $Tau^2 = 44.17$; $Chi^2 = 924.44$, df = 24 (P < 0.00001); $I^2 = 97\%$								
Test for overall effect: Z = 5.81 (P < 0.00001)								
Test for subgroup differences: $Chi^2 = 0.22$, df = 1 (P = 0.64), $I^2 = 0\%$								

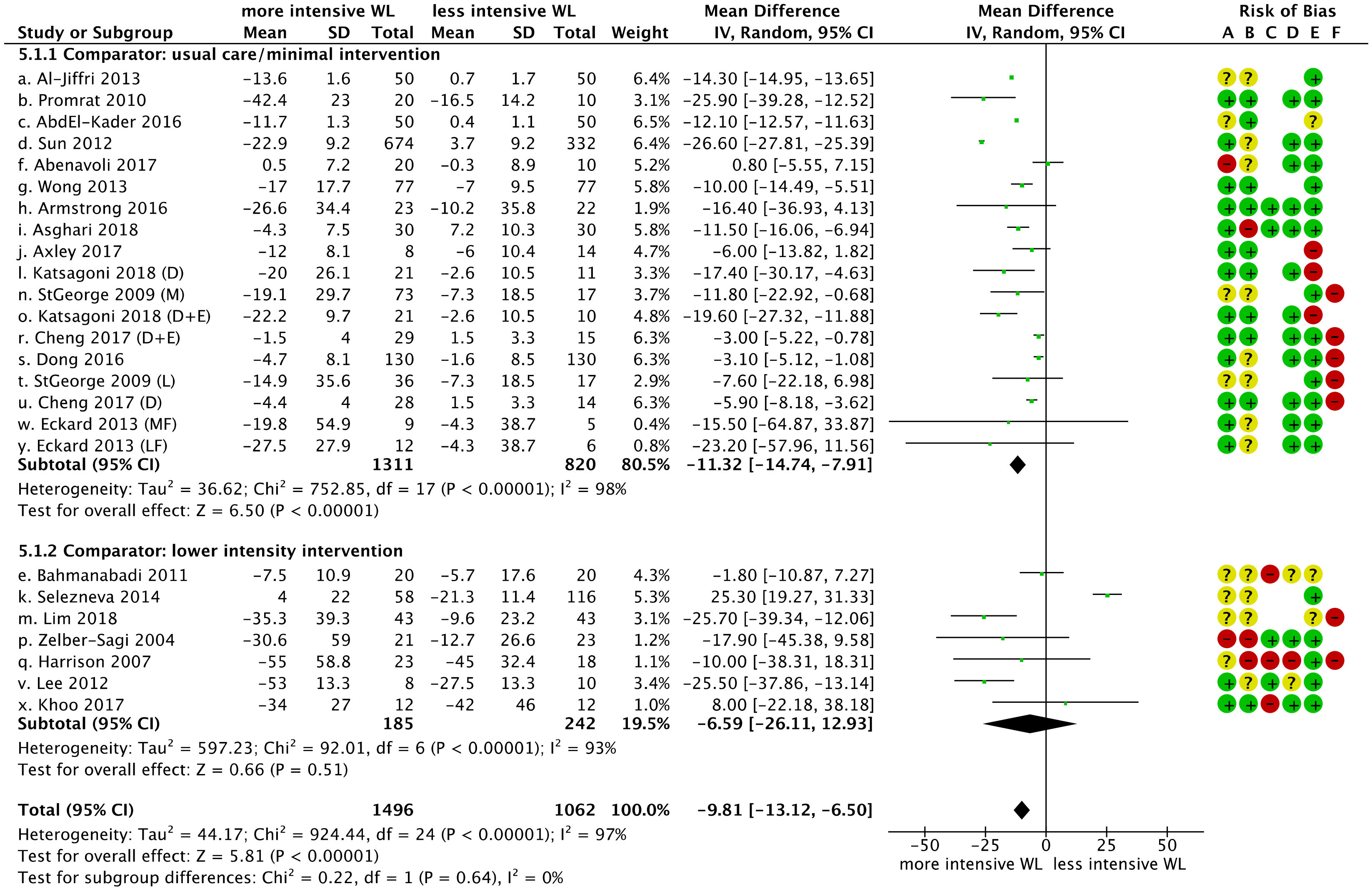

© 2019 Koutoukidis DA et al. *JAMA Internal Medicine*.

eFigure 6.6 AST (U/L) by the intensity of the comparator intervention

Study or Subgroup	more intensive WL Mean	SD	Total	less intensive WL Mean	SD	Total	Weight	Mean Difference IV, Random, 95% CI
**5.2.1 Comparator: usual care/minimal intervention**								
a. Al-Jiffri 2013	-11.6	2.2	50	0.7	1.8	50	7.2%	-12.30 [-13.09, -11.51]
b. Promrat 2010	-20.2	14.1	20	-18	30.8	10	1.3%	-2.20 [-22.26, 17.86]
c. AbdEl-Kader 2016	-9.5	1.6	50	0.4	1.4	50	7.2%	-9.90 [-10.49, -9.31]
d. Sun 2012	-5.7	15.2	674	-0.5	5.7	332	7.1%	-5.20 [-6.50, -3.90]
f. Abenavoli 2017	2.8	6.3	20	12.4	15.2	10	3.4%	-9.60 [-19.42, 0.22]
g. Wong 2013	-4	6.2	77	-3	5.4	77	7.0%	-1.00 [-2.84, 0.84]
h. Armstrong 2016	-15.8	21.8	23	-8.6	28.3	22	2.0%	-7.20 [-22.01, 7.61]
i. Asghari 2018	-4.1	3.6	30	4.4	11.8	30	5.9%	-8.50 [-12.91, -4.09]
j. Axley 2017	-9	2.2	8	0	2	14	7.0%	-9.00 [-10.85, -7.15]
n. StGeorge 2009 (M)	-6.9	18.3	36	-2.6	14.7	17	3.6%	-4.30 [-13.50, 4.90]
r. Cheng 2017 (D+E)	-4.9	2.4	29	4	2.3	14	7.1%	-8.90 [-10.39, -7.41]
s. Dong 2016	-1	3.5	130	0.4	4.9	130	7.2%	-1.40 [-2.44, -0.36]
t. StGeorge 2009 (L)	-8.3	16.1	73	-2.6	14.7	17	4.2%	-5.70 [-13.60, 2.20]
u. Cheng 2017 (D)	0.4	2.6	28	4	2.3	15	7.1%	-3.60 [-5.11, -2.09]
w. Eckard 2013 (MF)	-19.6	47.9	9	-2.9	25.8	5	0.4%	-16.70 [-55.31, 21.91]
y. Eckard 2013 (LF)	-15.9	19.1	12	-2.9	25.8	6	1.0%	-13.00 [-36.30, 10.30]
**Subtotal (95% CI)**			**1269**			**799**	**78.5%**	**-6.66 [-9.21, -4.10]**
Heterogeneity: $Tau^2 = 17.81$; $Chi^2 = 420.27$, df = 15 (P < 0.00001); $I^2 = 96\%$								
Test for overall effect: Z = 5.11 (P < 0.00001)								
**5.2.2 Comparator: lower intensity intervention**								
e. Bahmanabadi 2011	4.4	6.7	20	-1.7	7.9	20	5.8%	6.10 [1.56, 10.64]
k. Selezneva 2014	0.1	13.7	58	-11	16.4	116	5.8%	11.10 [6.48, 15.72]
m. Lim 2018	-19.3	26.1	43	-5.1	13.7	43	3.8%	-14.20 [-23.01, -5.39]
p. Zelber-Sagi 2004	-18.9	33	21	-8.8	17.2	23	1.8%	-10.10 [-25.87, 5.67]
q. Harrison 2007	-28	23.2	23	-29	18.4	18	2.5%	1.00 [-11.73, 13.73]
x. Khoo 2017	-18	15	12	-23	24	12	1.8%	5.00 [-11.01, 21.01]
**Subtotal (95% CI)**			**177**			**232**	**21.5%**	**0.62 [-7.52, 8.76]**
Heterogeneity: $Tau^2 = 75.27$; $Chi^2 = 29.07$, df = 5 (P < 0.0001); $I^2 = 83\%$								
Test for overall effect: Z = 0.15 (P = 0.88)								
**Total (95% CI)**			**1446**			**1031**	**100.0%**	**-4.84 [-7.31, -2.38]**
Heterogeneity: $Tau^2 = 21.83$; $Chi^2 = 530.14$, df = 21 (P < 0.00001); $I^2 = 96\%$								
Test for overall effect: Z = 3.85 (P = 0.0001)								
Test for subgroup differences: $Chi^2 = 2.79$, df = 1 (P = 0.09), $I^2 = 64.2\%$								

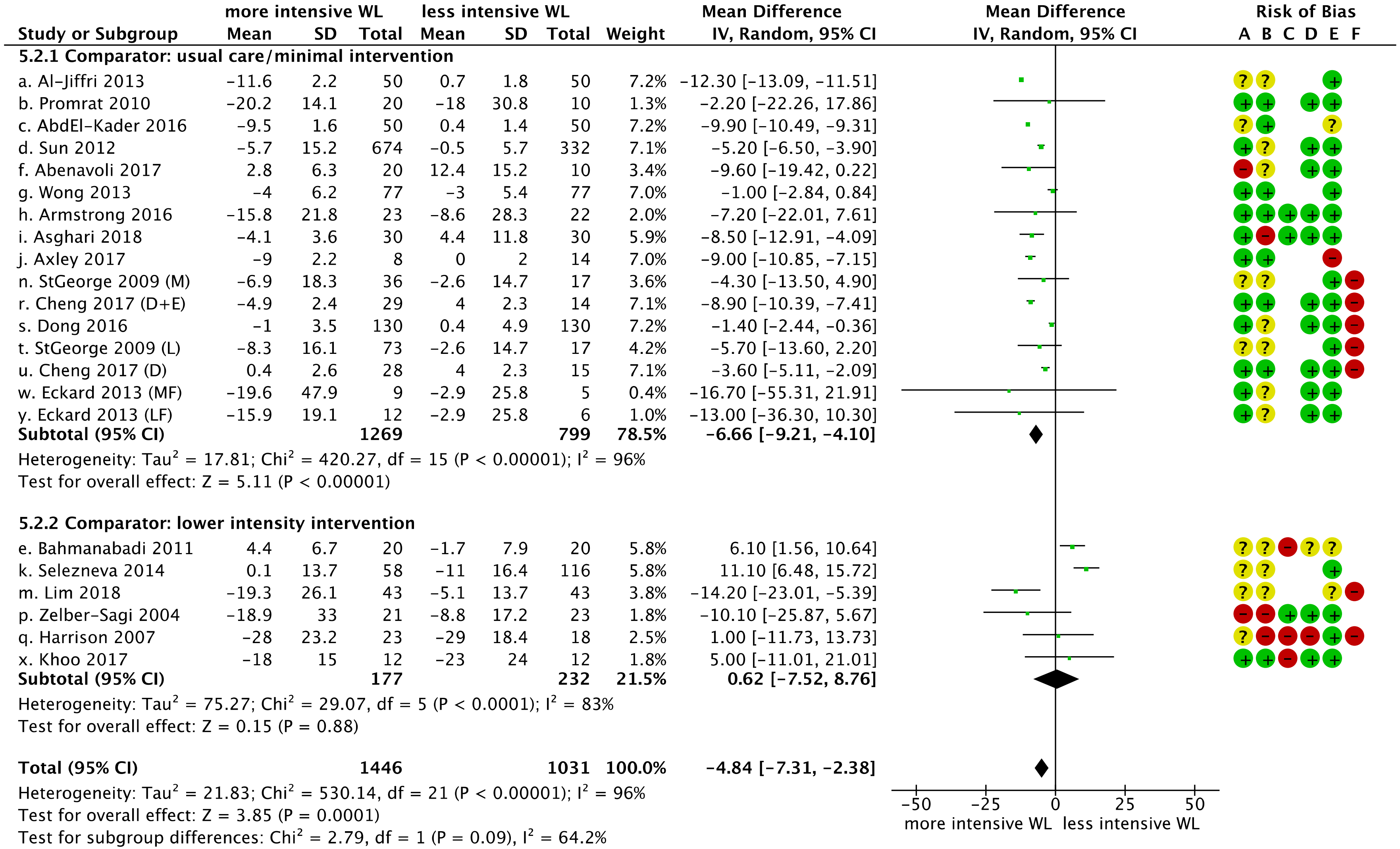

© 2019 Koutoukidis DA et al. *JAMA Internal Medicine*.

eFigure 6.7 Steatosis by the intensity of the comparator intervention

Study or Subgroup	more intensive WL Mean	more intensive WL SD	more intensive WL Total	less intensive WL Mean	less intensive WL SD	less intensive WL Total	Weight	Std. Mean Difference IV, Random, 95% CI
**4.3.1 Behavioural programmes**								
b. Promrat 2010	−1.1	0.4	18	−0.3	0.4	10	8.9%	−1.94 [−2.89, −0.99]
f. Abenavoli 2017	−1	0.2	20	0.1	0.2	10	7.2%	−5.35 [−6.99, −3.71]
g. Wong 2013	−6.8	3.1	77	−2.1	2.5	77	9.9%	−1.66 [−2.03, −1.29]
i. Asghari 2018	−0.1	0.2	24	0	0.1	26	9.6%	−0.63 [−1.20, −0.06]
r. Cheng 2017 (D+E)	−7.6	3.6	29	2.8	2.7	15	8.9%	−3.07 [−3.99, −2.15]
s. Dong 2016	−1.1	0.4	130	0	0.4	130	9.9%	−2.74 [−3.08, −2.40]
u. Cheng 2017 (D)	−5.4	3.3	28	2.8	2.7	14	9.0%	−2.58 [−3.45, −1.72]
**Subtotal (95% CI)**			**326**			**282**	**63.3%**	**−2.40 [−3.17, −1.62]**
Heterogeneity: Tau² = 0.91; Chi² = 65.41, df = 6 (P < 0.00001); I² = 91%								
Test for overall effect: Z = 6.07 (P < 0.00001)								
**4.3.2 Pharmacotherapy**								
h. Armstrong 2016	−0.7	0.8	23	−0.4	0.8	22	9.6%	−0.37 [−0.96, 0.22]
p. Zelber-Sagi 2004	0.1	0.3	11	−0.6	0.4	12	8.7%	1.90 [0.88, 2.91]
q. Harrison 2007	0	0.2	23	0	0.2	18	9.5%	0.00 [−0.62, 0.62]
z. Ye 2017	−7.8	3.1	14	−1.9	2.4	16	8.9%	−2.09 [−3.00, −1.18]
**Subtotal (95% CI)**			**71**			**68**	**36.7%**	**−0.16 [−1.41, 1.10]**
Heterogeneity: Tau² = 1.47; Chi² = 33.46, df = 3 (P < 0.00001); I² = 91%								
Test for overall effect: Z = 0.25 (P = 0.81)								
**Total (95% CI)**			**397**			**350**	**100.0%**	**−1.61 [−2.43, −0.80]**
Heterogeneity: Tau² = 1.72; Chi² = 178.92, df = 10 (P < 0.00001); I² = 94%								
Test for overall effect: Z = 3.88 (P = 0.0001)								
Test for subgroup differences: Chi² = 8.88, df = 1 (P = 0.003), I² = 88.7%								

Std. Mean Difference IV, Random, 95% CI — Risk of Bias A B C D E F

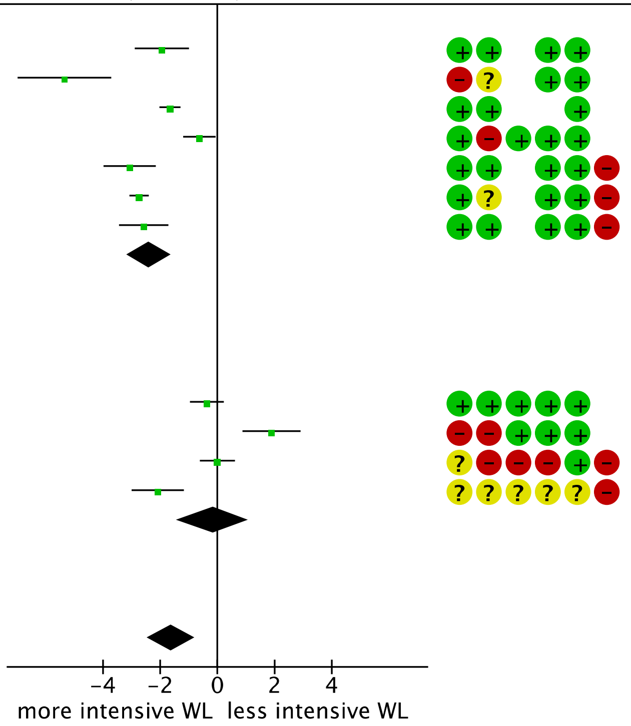

© 2019 Koutoukidis DA et al. *JAMA Internal Medicine*.

7 Subgroup analysis by the presence/absence of a minimum cut-off in the eligibility criteria for including only people with overweight/obesity

eFigure 7.1 Weight loss by the presence of a minimum overweight/obesity cut-off

Study or Subgroup	more intensive WL Mean	SD	Total	less intensive WL Mean	SD	Total	Weight	Mean Difference IV, Random, 95% CI
**6.24.1 With minimum BMI cut-off**								
a. Al-Jiffri 2013	-13.7	3.4	50	0.8	3	50	4.5%	-14.50 [-15.76, -13.24]
b. Promrat 2010	-8.7	12.4	20	-0.5	7.4	10	2.3%	-8.20 [-15.31, -1.09]
c. AbdEl-Kader 2016	-5.9	2.2	50	2.1	2.9	50	4.6%	-8.00 [-9.01, -6.99]
d. Sun 2012	-6.1	9.2	674	1.3	9.2	332	4.5%	-7.40 [-8.61, -6.19]
e. Bahmanabadi 2011	-13	10	20	-6.1	3	20	3.2%	-6.90 [-11.48, -2.32]
f. Abenavoli 2017	-5.1	3.4	20	0.4	2.3	10	4.3%	-5.50 [-7.56, -3.44]
h. Armstrong 2016	-5.3	4.7	23	-0.6	4.4	22	4.1%	-4.70 [-7.36, -2.04]
i. Asghari 2018	-4.1	4	30	0	2.7	30	4.4%	-4.10 [-5.83, -2.37]
j. Axley 2017	-2.7	5	8	0.9	6	14	3.2%	-3.60 [-8.28, 1.08]
l. Katsagoni 2018 (D)	-5.6	4.1	21	-2.8	3.9	11	4.0%	-2.80 [-5.70, 0.10]
m. Lim 2018	-3.2	2.5	43	-0.9	2	43	4.6%	-2.30 [-3.26, -1.34]
o. Katsagoni 2018 (D+E)	-4.9	4.1	21	-2.8	3.9	10	3.9%	-2.10 [-5.09, 0.89]
q. Harrison 2007	-8.2	5	23	-6.4	5.2	18	3.9%	-1.80 [-4.95, 1.35]
v. Lee 2012	-1.6	3.1	8	-0.8	2.3	10	4.1%	-0.80 [-3.38, 1.78]
x. Khoo 2017	-3.5	2.1	12	-3.5	3.3	12	4.2%	0.00 [-2.21, 2.21]
**Subtotal (95% CI)**			**1023**			**642**	**59.8%**	**-4.83 [-7.16, -2.49]**
Heterogeneity: Tau^2 = 19.01; Chi^2 = 322.62, df = 14 (P < 0.00001); I^2 = 96%								
Test for overall effect: Z = 4.05 (P < 0.0001)								
**6.24.2 No minimum BMI cut-off**								
g. Wong 2013	-5.6	5.7	77	-0.6	3.7	77	4.5%	-5.00 [-6.52, -3.48]
k. Selezneva 2014	-9.3	1.8	58	-6.2	1.7	116	4.6%	-3.10 [-3.66, -2.54]
n. StGeorge 2009 (M)	-2.8	3.4	73	-0.5	3.7	17	4.3%	-2.30 [-4.22, -0.38]
p. Zelber-Sagi 2004	-7.7	6.8	21	-5.9	5.9	23	3.6%	-1.80 [-5.58, 1.98]
r. Cheng 2017 (D+E)	-1.5	3.2	29	0.2	3.3	15	4.3%	-1.70 [-3.74, 0.34]
s. Dong 2016	-1.4	4.9	130	0.1	4.1	130	4.6%	-1.50 [-2.60, -0.40]
t. StGeorge 2009 (L)	-1.9	3.7	36	-0.5	3.7	17	4.3%	-1.40 [-3.53, 0.73]
u. Cheng 2017 (D)	-0.8	3.1	28	0.2	3.3	14	4.3%	-1.00 [-3.08, 1.08]
w. Eckard 2013 (MF)	-3	4.7	9	-2.5	5.3	5	2.8%	-0.50 [-6.07, 5.07]
y. Eckard 2013 (LF)	-0.2	5.4	12	-2.5	5.3	6	3.0%	2.30 [-2.93, 7.53]
**Subtotal (95% CI)**			**473**			**420**	**40.2%**	**-2.20 [-3.15, -1.25]**
Heterogeneity: Tau^2 = 1.17; Chi^2 = 24.81, df = 9 (P = 0.003); I^2 = 64%								
Test for overall effect: Z = 4.53 (P < 0.00001)								
**Total (95% CI)**			**1496**			**1062**	**100.0%**	**-3.61 [-5.11, -2.12]**
Heterogeneity: Tau^2 = 12.45; Chi^2 = 458.04, df = 24 (P < 0.00001); I^2 = 95%								
Test for overall effect: Z = 4.74 (P < 0.00001)								
Test for subgroup differences: Chi^2 = 4.18, df = 1 (P = 0.04), I^2 = 76.1%								

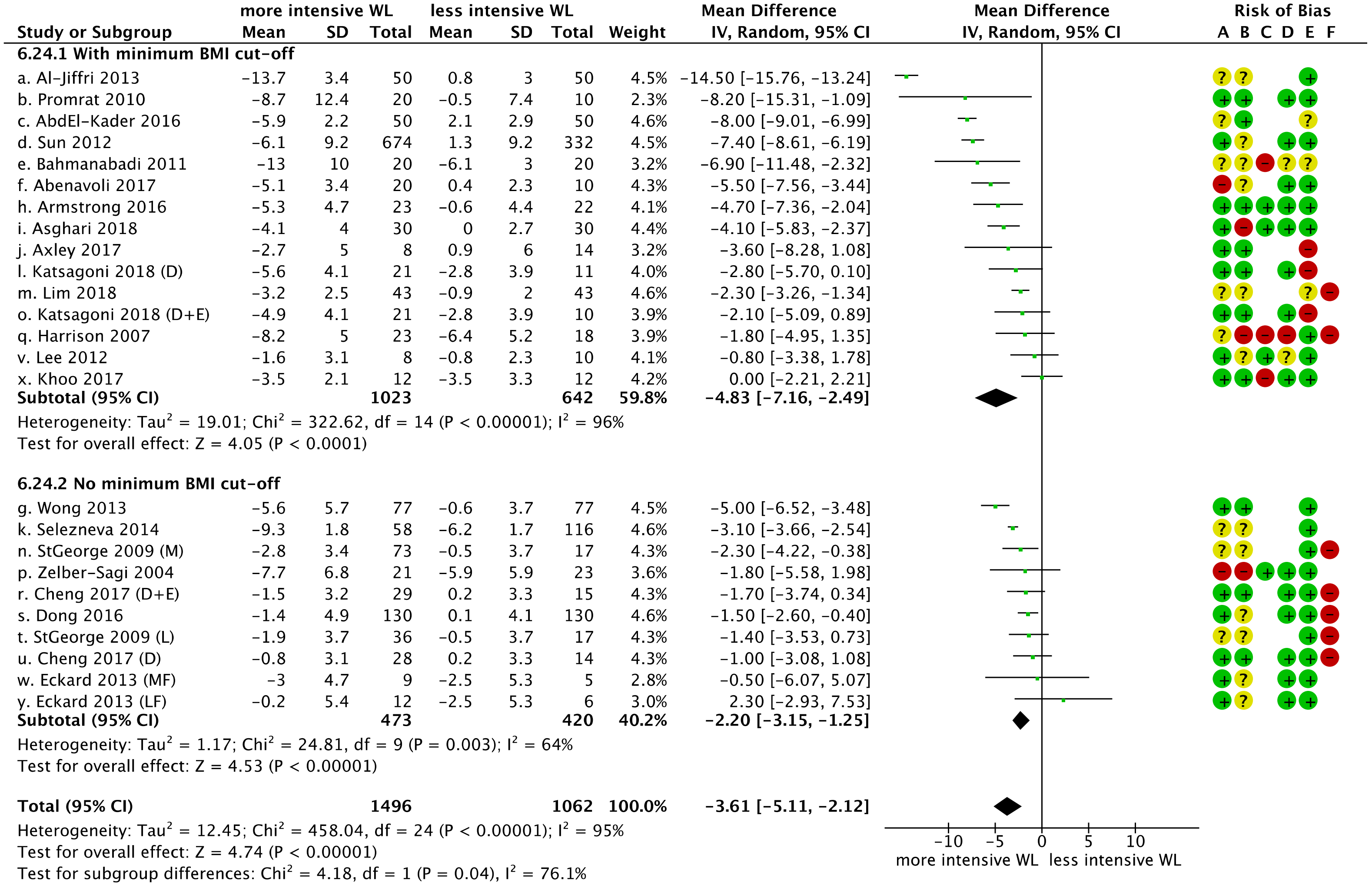

© 2019 Koutoukidis DA et al. *JAMA Internal Medicine*.

eFigure 7.2 Glucose (mmol/L) by the presence of a minimum overweight/obesity cut-off

Study or Subgroup	more intensive WL Mean	more intensive WL SD	more intensive WL Total	less intensive WL Mean	less intensive WL SD	less intensive WL Total	Weight	Mean Difference IV, Random, 95% CI
**6.21.1 With minimum BMI cut-off**								
b. Promrat 2010	−0.6	0.8	20	−1.7	3	10	1.2%	1.10 [−0.79, 2.99]
d. Sun 2012	−1.6	0.7	674	−0.2	1	332	6.9%	−1.40 [−1.52, −1.28]
f. Abenavoli 2017	−0.2	0.2	20	0	0.1	10	6.9%	−0.20 [−0.31, −0.09]
h. Armstrong 2016	−1	1.5	23	0.7	2.3	23	2.6%	−1.70 [−2.82, −0.58]
i. Asghari 2018	0.2	0.2	30	0.1	0.1	30	7.0%	0.10 [0.02, 0.18]
l. Katsagoni 2018 (D)	0	0.2	21	0.1	0.1	11	6.9%	−0.10 [−0.20, 0.00]
o. Katsagoni 2018 (D+E)	−0.1	0.1	21	0.1	0.1	10	7.0%	−0.20 [−0.28, −0.12]
q. Harrison 2007	−0.4	0.2	23	−0.7	1.8	18	3.6%	0.30 [−0.54, 1.14]
x. Khoo 2017	−0.7	0.6	12	−0.6	0.5	12	5.6%	−0.10 [−0.54, 0.34]
**Subtotal (95% CI)**			**844**			**456**	**47.8%**	**−0.31 [−0.68, 0.06]**
Heterogeneity: Tau² = 0.25; Chi² = 448.16, df = 8 (P < 0.00001); I² = 98%								
Test for overall effect: Z = 1.65 (P = 0.10)								
**6.21.2 No minimum BMI cut-off**								
g. Wong 2013	0	0.6	77	0.1	0.6	77	6.7%	−0.10 [−0.29, 0.09]
n. StGeorge 2009 (M)	−0.2	0.7	73	0	0.7	17	5.9%	−0.20 [−0.57, 0.17]
p. Zelber-Sagi 2004	−7.1	0.9	21	−5.1	0.3	23	5.8%	−2.00 [−2.40, −1.60]
r. Cheng 2017 (D+E)	0.1	0.2	29	0.2	0.2	15	6.9%	−0.10 [−0.22, 0.02]
s. Dong 2016	0	0.5	130	0.2	0.5	130	6.9%	−0.20 [−0.32, −0.08]
t. StGeorge 2009 (L)	−0.3	0.6	36	0	0.7	17	5.9%	−0.30 [−0.69, 0.09]
u. Cheng 2017 (D)	0.3	0.2	28	0.2	0.2	14	6.9%	0.10 [−0.03, 0.23]
w. Eckard 2013 (MF)	−0.1	1.1	9	0.1	0.5	5	3.6%	−0.20 [−1.04, 0.64]
y. Eckard 2013 (LF)	−0.5	1.3	12	0.1	0.5	6	3.6%	−0.60 [−1.44, 0.24]
**Subtotal (95% CI)**			**415**			**304**	**52.2%**	**−0.36 [−0.62, −0.10]**
Heterogeneity: Tau² = 0.12; Chi² = 98.31, df = 8 (P < 0.00001); I² = 92%								
Test for overall effect: Z = 2.68 (P = 0.007)								
**Total (95% CI)**			**1259**			**760**	**100.0%**	**−0.34 [−0.57, −0.12]**
Heterogeneity: Tau² = 0.19; Chi² = 554.22, df = 17 (P < 0.00001); I² = 97%								
Test for overall effect: Z = 2.96 (P = 0.003)								
Test for subgroup differences: Chi² = 0.04, df = 1 (P = 0.84), I² = 0%								

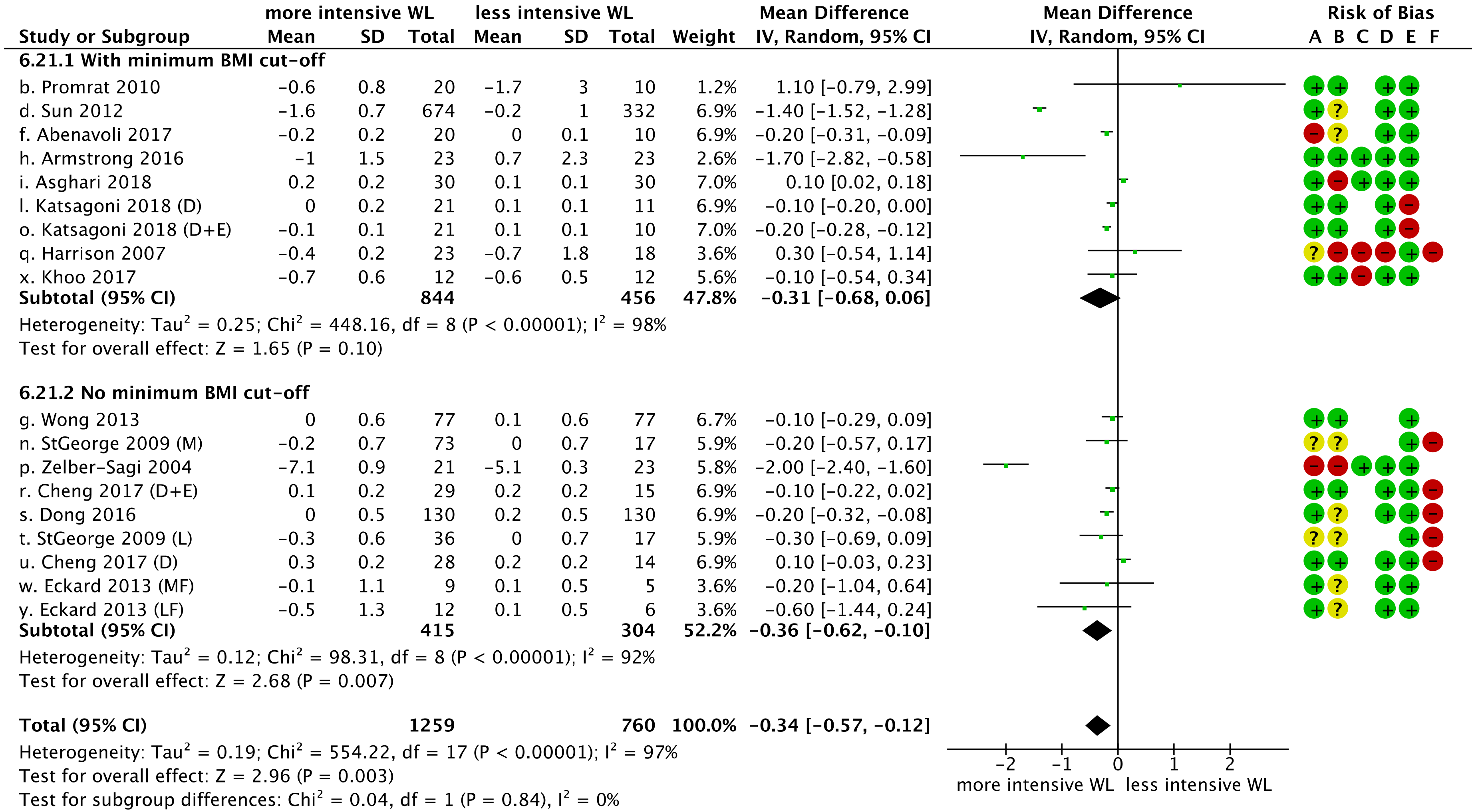

© 2019 Koutoukidis DA et al. *JAMA Internal Medicine*.

eFigure 7.3 Insulin resistance index (HOMA-IR or other) by the presence of a minimum overweight/obesity cut-off

	more intensive WL			less intensive WL				Std. Mean Difference	Risk of Bias					
Study or Subgroup	Mean	SD	Total	Mean	SD	Total	Weight	IV, Random, 95% CI	A	B	C	D	E	F
**6.22.1 With minimum BMI cut-off**														
a. Al-Jiffri 2013	-1.4	1.6	50	0.1	0.6	50	5.6%	-1.23 [-1.66, -0.80]	?	?			+	
b. Promrat 2010	-2	3	20	0.1	2.3	10	5.5%	-0.73 [-1.52, 0.05]	+	+		+	+	
c. AbdEl-Kader 2016	-2	0.7	50	0.3	0.5	50	5.6%	-3.75 [-4.41, -3.09]	?	+			?	
d. Sun 2012	-1.5	0.3	674	0.5	0.3	332	5.6%	-6.66 [-6.98, -6.34]	+	?		+	+	
f. Abenavoli 2017	0.2	0.5	20	0.6	0.3	10	5.5%	-0.87 [-1.67, -0.08]	-	?		+	+	
h. Armstrong 2016	-1.8	3.7	23	0.7	9.5	22	5.6%	-0.34 [-0.93, 0.25]	+	+	+	+	+	
i. Asghari 2018	-0.2	0.5	30	0.7	1.9	30	5.6%	-0.64 [-1.16, -0.12]	+	-	+	+	+	
l. Katsagoni 2018 (D)	-0.5	0.8	21	-0.3	0.6	11	5.5%	-0.26 [-1.00, 0.47]	+	+		+	-	
o. Katsagoni 2018 (D+E)	-0.8	0.5	21	-0.3	0.6	10	5.5%	-0.91 [-1.70, -0.12]	+	+		+	-	
q. Harrison 2007	0.01	0.014	23	0.01	0.007	18	5.6%	0.00 [-0.62, 0.62]	?	-	-	-	+	-
x. Khoo 2017	-2.9	2.3	12	-3.2	2.6	12	5.5%	0.12 [-0.68, 0.92]	+	+	-	+	+	
**Subtotal (95% CI)**			**944**			**555**	**61.2%**	**-1.40 [-3.13, 0.34]**						
Heterogeneity: Tau² = 8.50; Chi² = 989.15, df = 10 (P < 0.00001); I² = 99%														
Test for overall effect: Z = 1.58 (P = 0.11)														
**6.22.2 No minimum BMI cut-off**														
n. StGeorge 2009 (M)	-0.2	2.3	36	0	1.5	17	5.6%	-0.09 [-0.67, 0.48]	?	?			+	-
p. Zelber-Sagi 2004	-6.2	19.6	21	-0.8	3	23	5.6%	-0.39 [-0.98, 0.21]	-	-	+	+	+	
r. Cheng 2017 (D+E)	-0.1	0.5	29	0.2	0.6	15	5.6%	-0.55 [-1.19, 0.08]	+	+		+	+	-
t. StGeorge 2009 (L)	-0.4	1	73	0	1.5	17	5.6%	-0.36 [-0.89, 0.17]	?	?			+	-
u. Cheng 2017 (D)	0.2	0.5	28	0.2	0.6	14	5.6%	0.00 [-0.64, 0.64]	+	+		+	+	-
w. Eckard 2013 (MF)	-0.008	0.019	9	0	0.03	5	5.4%	-0.32 [-1.42, 0.78]	+	?		+	+	
y. Eckard 2013 (LF)	0.02	0.02	12	0	0.03	6	5.4%	0.81 [-0.22, 1.83]	+	?		+	+	
**Subtotal (95% CI)**			**208**			**97**	**38.8%**	**-0.22 [-0.47, 0.04]**						
Heterogeneity: Tau² = 0.00; Chi² = 6.13, df = 6 (P = 0.41); I² = 2%														
Test for overall effect: Z = 1.68 (P = 0.09)														
**Total (95% CI)**			**1152**			**652**	**100.0%**	**-0.91 [-2.11, 0.30]**						
Heterogeneity: Tau² = 6.70; Chi² = 1219.38, df = 17 (P < 0.00001); I² = 99%														
Test for overall effect: Z = 1.47 (P = 0.14)														
Test for subgroup differences: Chi² = 1.74, df = 1 (P = 0.19), I² = 42.5%														

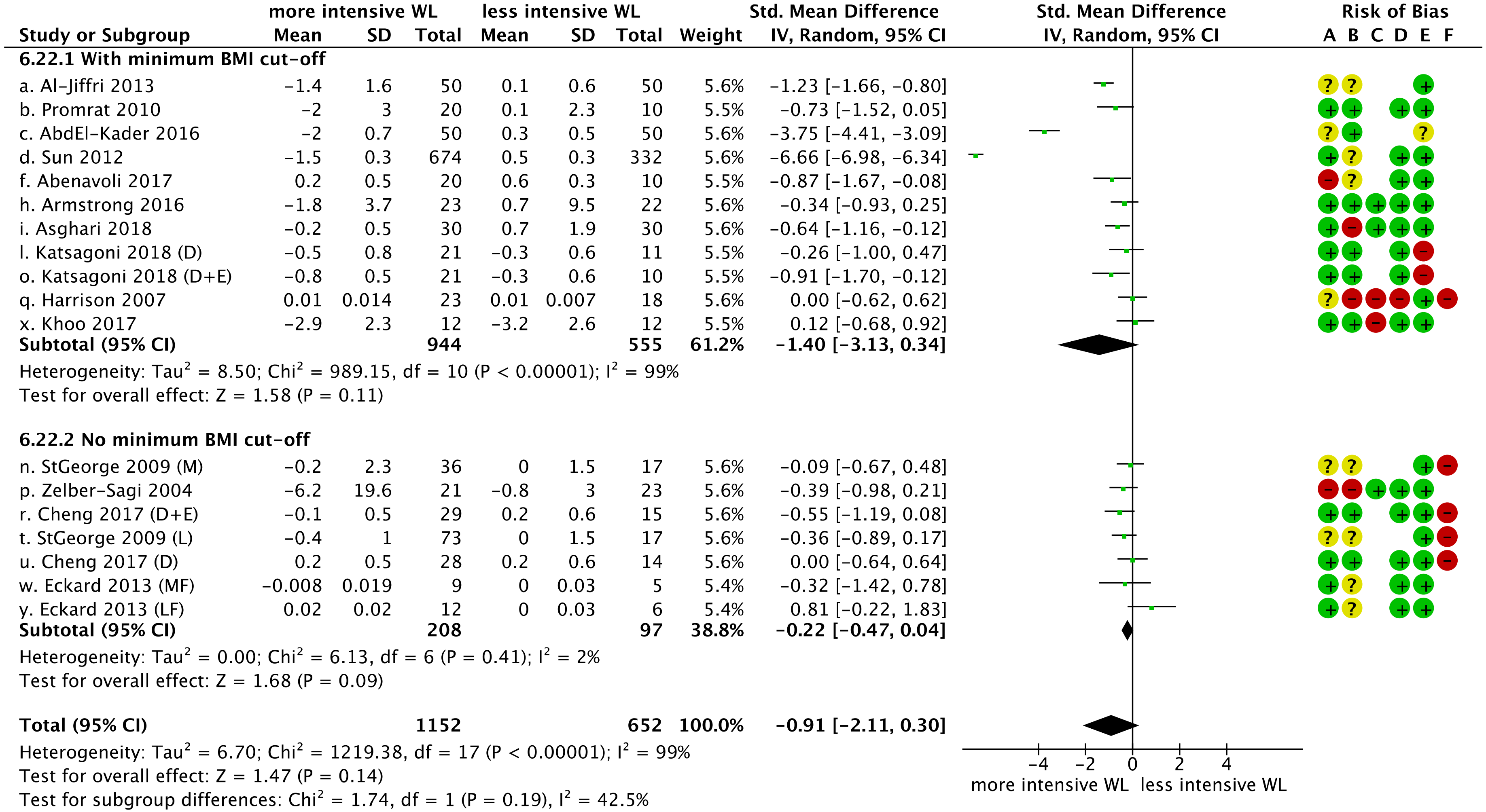

© 2019 Koutoukidis DA et al. *JAMA Internal Medicine*.

eFigure 7.4 Insulin (pmol/L) by the presence of a minimum overweight/obesity cut-off

	more intensive WL			less intensive WL				Mean Difference	Risk of Bias					
Study or Subgroup	Mean	SD	Total	Mean	SD	Total	Weight	IV, Random, 95% CI	A	B	C	D	E	F
**6.2.1 With minimum BMI cut-off**														
b. Promrat 2010	-31.2	58.8	20	18	30.6	10	5.8%	-49.20 [-81.20, -17.20]	+	+		+	+	
d. Sun 2012	-27	31.8	674	22.2	25.2	332	8.2%	-49.20 [-52.82, -45.58]	+	?		+	+	
f. Abenavoli 2017	4.8	10.8	20	10.2	9	10	8.1%	-5.40 [-12.72, 1.92]	-	?		+	+	
h. Armstrong 2016	-15.9	54.7	23	-34.7	164.1	22	2.7%	18.80 [-53.32, 90.92]	+	+	+	+	+	
i. Asghari 2018	-7.8	12	30	16.8	52.8	30	7.2%	-24.60 [-43.98, -5.22]	+	-	+	+	+	
l. Katsagoni 2018 (D)	-30.8	16.6	21	-6.5	14	11	7.9%	-24.30 [-35.20, -13.40]	+	+		+	-	
o. Katsagoni 2018 (D+E)	-14.3	18.7	21	-6.5	14	10	7.8%	-7.80 [-19.60, 4.00]	+	+		+	-	
q. Harrison 2007	-24	69.6	23	-6	16.8	18	6.1%	-18.00 [-47.48, 11.48]	?	-	-	-	+	-
x. Khoo 2017	-54.6	45.6	12	-56.4	37.2	12	5.7%	1.80 [-31.50, 35.10]	+	+	-	+	+	
**Subtotal (95% CI)**			**844**			**455**	**59.5%**	**-20.46 [-38.62, -2.29]**						
Heterogeneity: $Tau^2 = 614.36$; $Chi^2 = 155.12$, df = 8 (P < 0.00001); $I^2 = 95\%$														
Test for overall effect: Z = 2.21 (P = 0.03)														
**6.2.2 No minimum BMI cut-off**														
n. StGeorge 2009 (M)	4.2	25.2	36	-1.8	27	17	7.5%	6.00 [-9.25, 21.25]	?	?			+	-
p. Zelber-Sagi 2004	-81	235.8	21	-18.6	74.4	23	1.5%	-62.40 [-167.74, 42.94]	-	-	+	+	+	
r. Cheng 2017 (D+E)	-4.2	24	29	0.6	35.4	15	7.1%	-4.80 [-24.73, 15.13]	+	+		+	+	-
t. StGeorge 2009 (L)	-9	22.2	73	-1.8	27	17	7.7%	-7.20 [-21.01, 6.61]	?	?			+	-
u. Cheng 2017 (D)	-0.6	19.2	28	0.6	35.4	14	7.1%	-1.20 [-21.06, 18.66]	+	+		+	+	-
w. Eckard 2013 (MF)	15.6	36	9	-6.6	41.4	5	4.7%	22.20 [-21.04, 65.44]	+	?		+	+	
y. Eckard 2013 (LF)	-31.2	44.4	12	-6.6	41.4	6	4.9%	-24.60 [-66.17, 16.97]	+	?		+	+	
**Subtotal (95% CI)**			**208**			**97**	**40.5%**	**-2.22 [-10.17, 5.74]**						
Heterogeneity: $Tau^2 = 0.00$; $Chi^2 = 5.28$, df = 6 (P = 0.51); $I^2 = 0\%$														
Test for overall effect: Z = 0.55 (P = 0.58)														
**Total (95% CI)**			**1052**			**552**	**100.0%**	**-13.75 [-28.04, 0.53]**						
Heterogeneity: $Tau^2 = 643.40$; $Chi^2 = 222.99$, df = 15 (P < 0.00001); $I^2 = 93\%$														
Test for overall effect: Z = 1.89 (P = 0.06)														
Test for subgroup differences: $Chi^2 = 3.25$, df = 1 (P = 0.07), $I^2 = 69.2\%$														

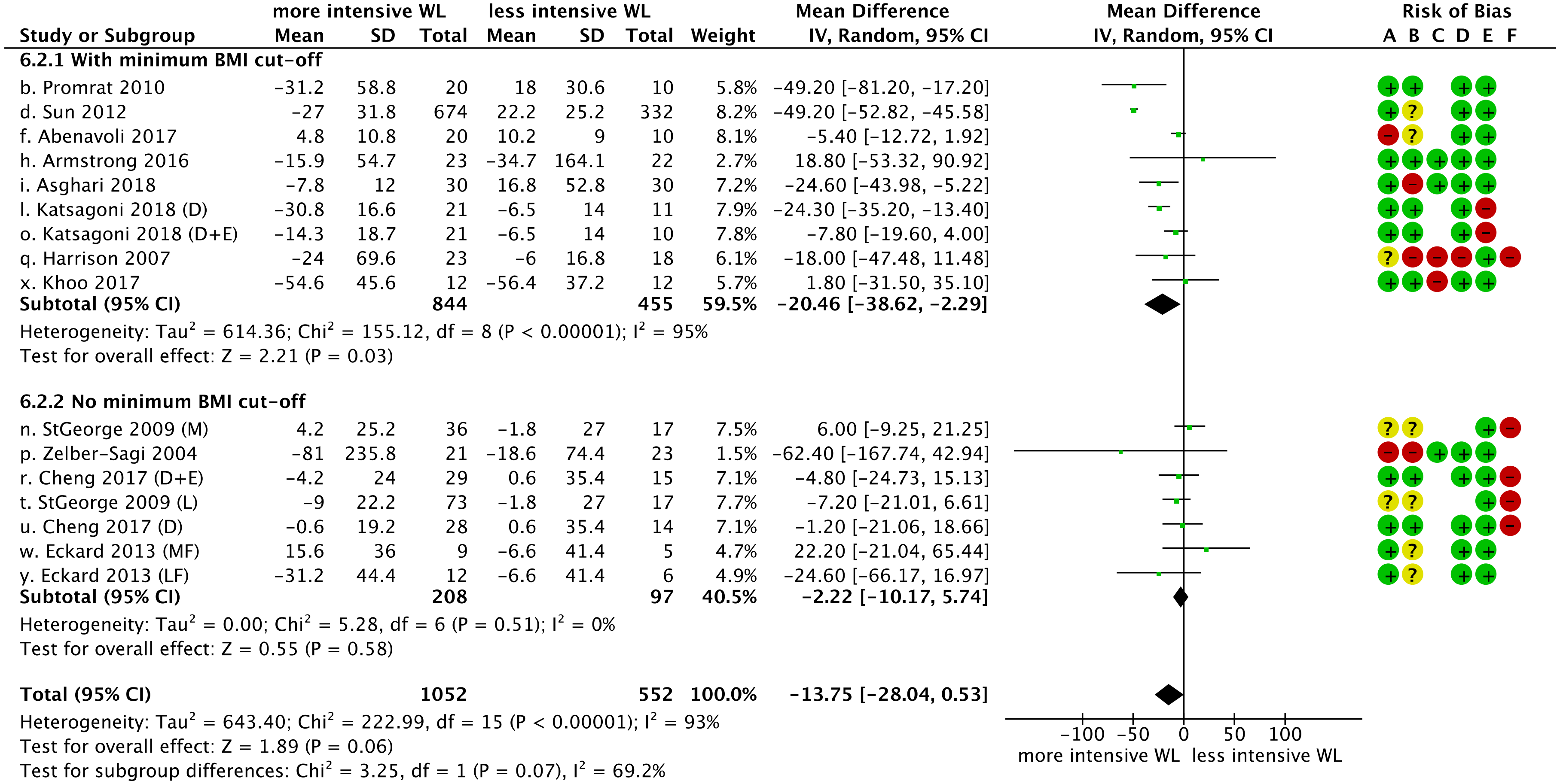

© 2019 Koutoukidis DA et al. *JAMA Internal Medicine*.

eFigure 7.5 ALT (U/L) by the presence of a minimum overweight/obesity cut-off

Study or Subgroup	more intensive WL Mean	more intensive WL SD	more intensive WL Total	less intensive WL Mean	less intensive WL SD	less intensive WL Total	Weight	Mean Difference IV, Random, 95% CI	Risk of Bias A	B	C	D	E	F
**6.1.1 With minimum BMI cut-off**														
a. Al-Jiffri 2013	-13.6	1.6	50	0.7	1.7	50	6.4%	-14.30 [-14.95, -13.65]	?	?			+	
b. Promrat 2010	-42.4	23	20	-16.5	14.2	10	3.1%	-25.90 [-39.28, -12.52]	+	+		+	+	
c. AbdEl-Kader 2016	-11.7	1.3	50	0.4	1.1	50	6.5%	-12.10 [-12.57, -11.63]	?	+			?	
d. Sun 2012	-22.9	9.2	674	3.7	9.2	332	6.4%	-26.60 [-27.81, -25.39]	+	?		+	+	
e. Bahmanabadi 2011	-7.5	10.9	20	-5.7	17.6	20	4.3%	-1.80 [-10.87, 7.27]	?	?	−	?	?	
f. Abenavoli 2017	0.5	7.2	20	-0.3	8.9	10	5.2%	0.80 [-5.55, 7.15]	−	?		+	+	
h. Armstrong 2016	-26.6	34.4	23	-10.2	35.8	22	1.9%	-16.40 [-36.93, 4.13]	+	+	+	+	+	
i. Asghari 2018	-4.3	7.5	30	7.2	10.3	30	5.8%	-11.50 [-16.06, -6.94]	+	−	+	+	+	
j. Axley 2017	-12	8.1	8	-6	10.4	14	4.7%	-6.00 [-13.82, 1.82]	+	+			−	
l. Katsagoni 2018 (D)	-20	26.1	21	-2.6	10.5	11	3.3%	-17.40 [-30.17, -4.63]	+	+		+	−	
m. Lim 2018	-35.3	39.3	43	-9.6	23.2	43	3.1%	-25.70 [-39.34, -12.06]	?	?			?	−
o. Katsagoni 2018 (D+E)	-22.2	9.7	21	-2.6	10.5	10	4.8%	-19.60 [-27.32, -11.88]	+	+		+	−	
q. Harrison 2007	-55	58.8	23	-45	32.4	18	1.1%	-10.00 [-38.31, 18.31]	?	−	−	−	+	−
v. Lee 2012	-53	13.3	8	-27.5	13.3	10	3.4%	-25.50 [-37.86, -13.14]	+	?	+	?	+	
x. Khoo 2017	-34	27	12	-42	46	12	1.0%	8.00 [-22.18, 38.18]	+	+	−	+	+	
**Subtotal (95% CI)**			**1023**			**642**	**61.1%**	**-14.25 [-17.97, -10.53]**						
Heterogeneity: Tau² = 31.10; Chi² = 526.84, df = 14 (P < 0.00001); I² = 97%														
Test for overall effect: Z = 7.50 (P < 0.00001)														
**6.1.2 No BMI cut-off**														
g. Wong 2013	-17	17.7	77	-7	9.5	77	5.8%	-10.00 [-14.49, -5.51]	+	+			+	
k. Selezneva 2014	4	22	58	-21.3	11.4	116	5.3%	25.30 [19.27, 31.33]	?	?			+	
n. StGeorge 2009 (M)	-19.1	29.7	73	-7.3	18.5	17	3.7%	-11.80 [-22.92, -0.68]	?	?			+	−
p. Zelber-Sagi 2004	-30.6	59	21	-12.7	26.6	23	1.2%	-17.90 [-45.38, 9.58]	−	−	+	+	+	
r. Cheng 2017 (D+E)	-1.5	4	29	1.5	3.3	15	6.3%	-3.00 [-5.22, -0.78]	+	+		+	+	−
s. Dong 2016	-4.7	8.1	130	-1.6	8.5	130	6.3%	-3.10 [-5.12, -1.08]	+	?		+	+	−
t. StGeorge 2009 (L)	-14.9	35.6	36	-7.3	18.5	17	2.9%	-7.60 [-22.18, 6.98]	?	?			+	−
u. Cheng 2017 (D)	-4.4	4	28	1.5	3.3	14	6.3%	-5.90 [-8.18, -3.62]	+	+		+	+	−
w. Eckard 2013 (MF)	-19.8	54.9	9	-4.3	38.7	5	0.4%	-15.50 [-64.87, 33.87]	+	?		+	+	
y. Eckard 2013 (LF)	-27.5	27.9	12	-4.3	38.7	6	0.8%	-23.20 [-57.96, 11.56]	+	?		+	+	
**Subtotal (95% CI)**			**473**			**420**	**38.9%**	**-2.88 [-8.29, 2.53]**						
Heterogeneity: Tau² = 46.31; Chi² = 105.23, df = 9 (P < 0.00001); I² = 91%														
Test for overall effect: Z = 1.04 (P = 0.30)														
**Total (95% CI)**			**1496**			**1062**	**100.0%**	**-9.81 [-13.12, -6.50]**						
Heterogeneity: Tau² = 44.17; Chi² = 924.44, df = 24 (P < 0.00001); I² = 97%														
Test for overall effect: Z = 5.81 (P < 0.00001)														
Test for subgroup differences: Chi² = 11.52, df = 1 (P = 0.0007), I² = 91.3%														

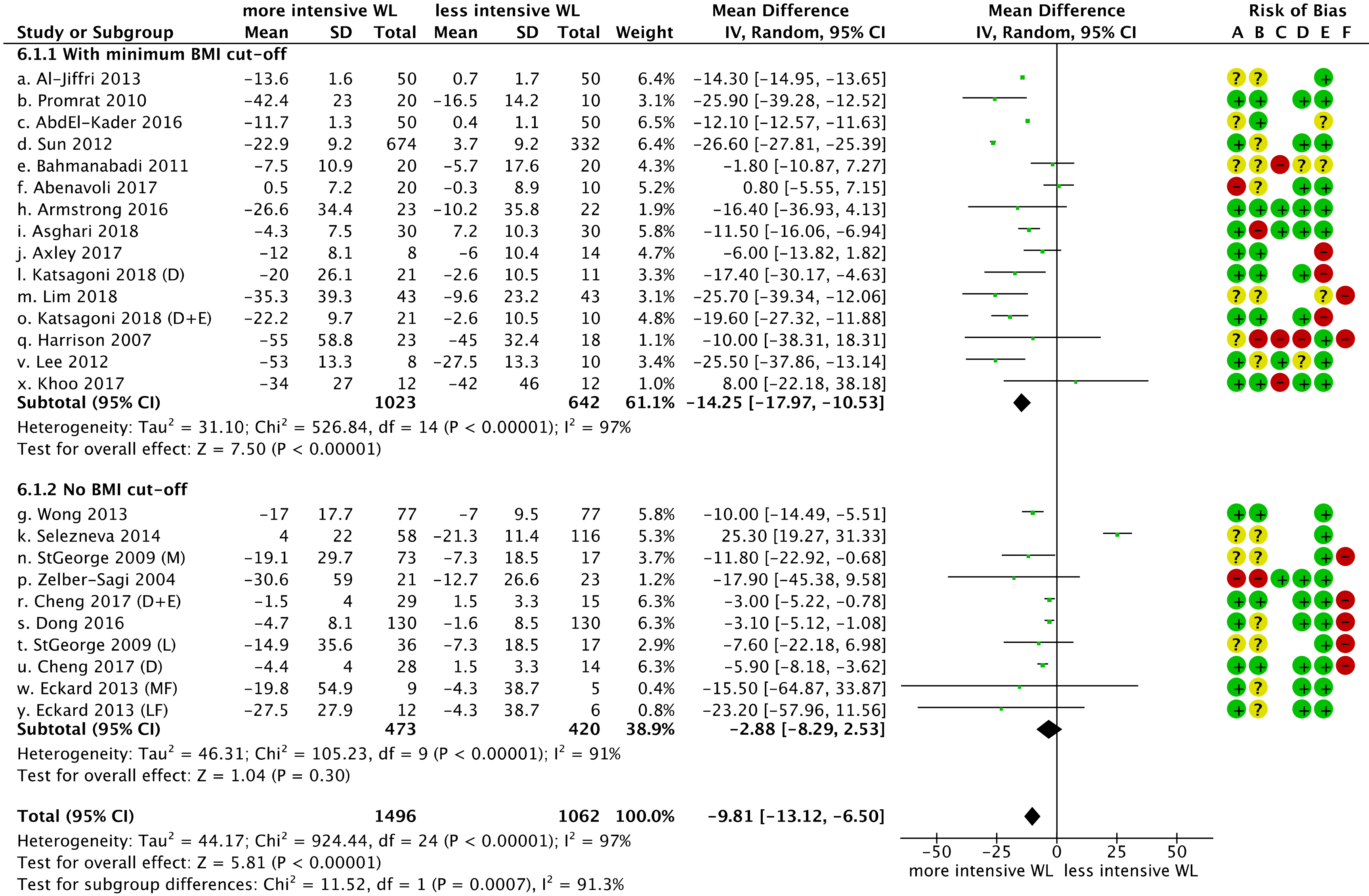

© 2019 Koutoukidis DA et al. *JAMA Internal Medicine*.

eFigure 7.6 AST (U/L) by the presence of a minimum overweight/obesity cut-off

Study or Subgroup	more intensive WL Mean	SD	Total	less intensive WL Mean	SD	Total	Weight	Mean Difference IV, Random, 95% CI
**6.3.1 With minimum BMI cut-off**								
a. Al-Jiffri 2013	-11.6	2.2	50	0.7	1.8	50	7.2%	-12.30 [-13.09, -11.51]
b. Promrat 2010	-20.2	14.1	20	-18	30.8	10	1.3%	-2.20 [-22.26, 17.86]
c. AbdEl-Kader 2016	-9.5	1.6	50	0.4	1.4	50	7.2%	-9.90 [-10.49, -9.31]
d. Sun 2012	-5.7	15.2	674	-0.5	5.7	332	7.1%	-5.20 [-6.50, -3.90]
e. Bahmanabadi 2011	4.4	6.7	20	-1.7	7.9	20	5.8%	6.10 [1.56, 10.64]
f. Abenavoli 2017	2.8	6.3	20	12.4	15.2	10	3.4%	-9.60 [-19.42, 0.22]
h. Armstrong 2016	-15.8	21.8	23	-8.6	28.3	22	2.0%	-7.20 [-22.01, 7.61]
i. Asghari 2018	-4.1	3.6	30	4.4	11.8	30	5.9%	-8.50 [-12.91, -4.09]
j. Axley 2017	-9	2.2	8	0	2	14	7.0%	-9.00 [-10.85, -7.15]
m. Lim 2018	-19.3	26.1	43	-5.1	13.7	43	3.8%	-14.20 [-23.01, -5.39]
n. StGeorge 2009 (M)	-6.9	18.3	36	-2.6	14.7	17	3.6%	-4.30 [-13.50, 4.90]
q. Harrison 2007	-28	23.2	23	-29	18.4	18	2.5%	1.00 [-11.73, 13.73]
x. Khoo 2017	-18	15	12	-23	24	12	1.8%	5.00 [-11.01, 21.01]
**Subtotal (95% CI)**			**1009**			**628**	**58.6%**	**-6.93 [-9.32, -4.53]**
Heterogeneity: Tau^2 = 9.88; Chi^2 = 143.94, df = 12 (P < 0.00001); I^2 = 92%								
Test for overall effect: Z = 5.67 (P < 0.00001)								
**6.3.2 No minimum BMI cut-off**								
g. Wong 2013	-4	6.2	77	-3	5.4	77	7.0%	-1.00 [-2.84, 0.84]
k. Selezneva 2014	0.1	13.7	58	-11	16.4	116	5.8%	11.10 [6.48, 15.72]
p. Zelber-Sagi 2004	-18.9	33	21	-8.8	17.2	23	1.8%	-10.10 [-25.87, 5.67]
r. Cheng 2017 (D+E)	-4.9	2.4	29	4	2.3	14	7.1%	-8.90 [-10.39, -7.41]
s. Dong 2016	-1	3.5	130	0.4	4.9	130	7.2%	-1.40 [-2.44, -0.36]
t. StGeorge 2009 (L)	-8.3	16.1	73	-2.6	14.7	17	4.2%	-5.70 [-13.60, 2.20]
u. Cheng 2017 (D)	0.4	2.6	28	4	2.3	15	7.1%	-3.60 [-5.11, -2.09]
w. Eckard 2013 (MF)	-19.6	47.9	9	-2.9	25.8	5	0.4%	-16.70 [-55.31, 21.91]
y. Eckard 2013 (LF)	-15.9	19.1	12	-2.9	25.8	6	1.0%	-13.00 [-36.30, 10.30]
**Subtotal (95% CI)**			**437**			**403**	**41.4%**	**-2.40 [-5.88, 1.08]**
Heterogeneity: Tau^2 = 17.39; Chi^2 = 112.80, df = 8 (P < 0.00001); I^2 = 93%								
Test for overall effect: Z = 1.35 (P = 0.18)								
**Total (95% CI)**			**1446**			**1031**	**100.0%**	**-4.84 [-7.31, -2.38]**
Heterogeneity: Tau^2 = 21.83; Chi^2 = 530.14, df = 21 (P < 0.00001); I^2 = 96%								
Test for overall effect: Z = 3.85 (P = 0.0001)								
Test for subgroup differences: Chi^2 = 4.41, df = 1 (P = 0.04), I^2 = 77.3%								

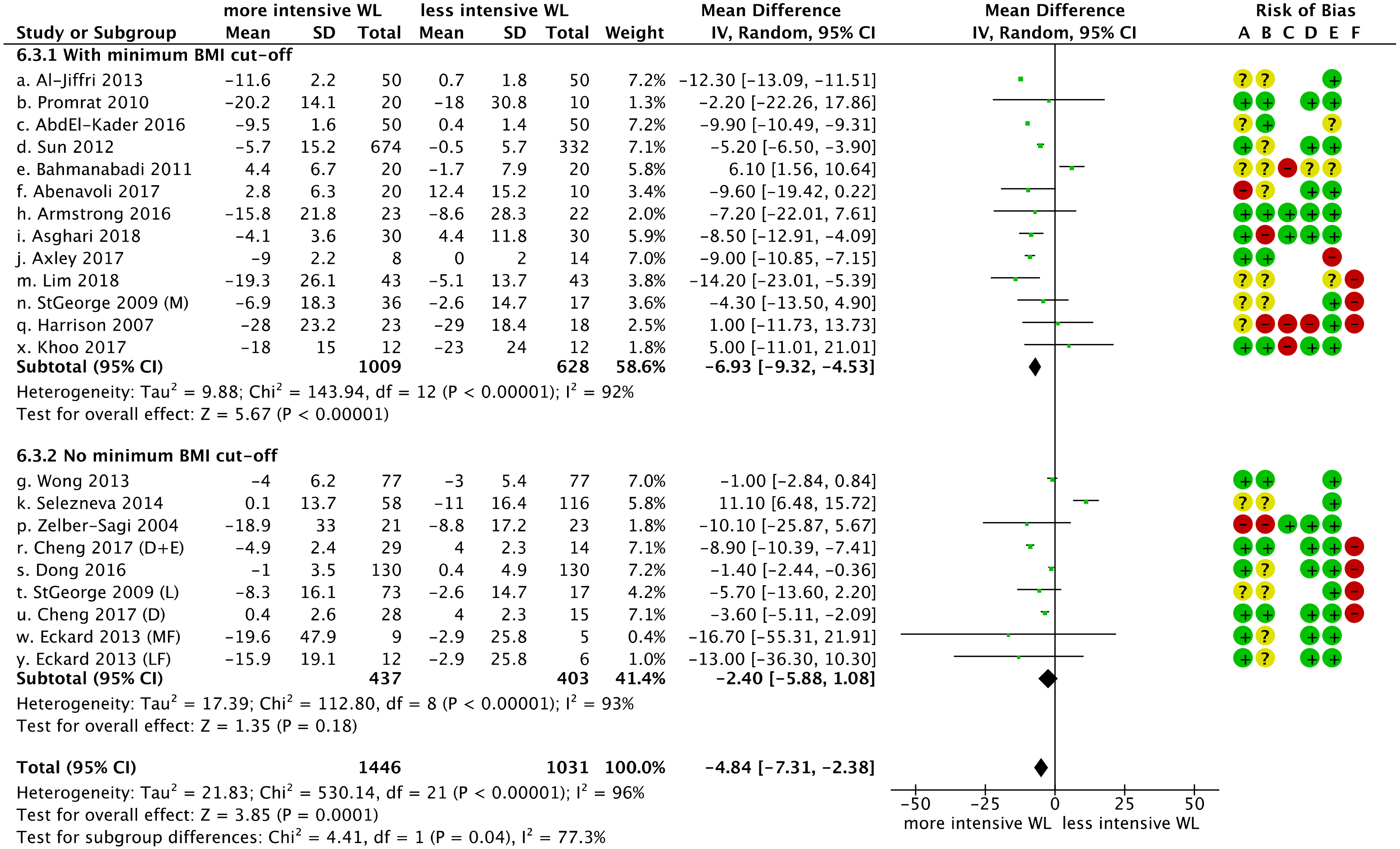

© 2019 Koutoukidis DA et al. *JAMA Internal Medicine*.

eFigure 7.7 Steatosis (Standardized Mean Difference as Assessed by Histologic Examination, MRI, or Ultrasonography) by the Presence of a Minimum Overweight/Obesity Cut-off

Study or Subgroup	more intensive WL Mean	SD	Total	less intensive WL Mean	SD	Total	Weight	Std. Mean Difference IV, Random, 95% CI
**6.15.1 With minimum BMI cut-off**								
b. Promrat 2010	-1.1	0.4	18	-0.3	0.4	10	8.1%	-1.94 [-2.89, -0.99]
f. Abenavoli 2017	-1	0.2	20	0.1	0.2	10	6.6%	-5.35 [-6.99, -3.71]
g. Wong 2013	-6.8	3.1	77	-2.1	2.5	77	9.0%	-1.66 [-2.03, -1.29]
h. Armstrong 2016	-0.7	0.8	23	-0.4	0.8	22	8.8%	-0.37 [-0.96, 0.22]
i. Asghari 2018	-0.1	0.2	24	0	0.1	26	8.8%	-0.63 [-1.20, -0.06]
q. Harrison 2007	0	0.2	23	0	0.2	18	8.7%	0.00 [-0.62, 0.62]
v. Lee 2012	-1	0.3	8	-1	0.3	10	8.2%	0.00 [-0.93, 0.93]
z. Ye 2017	-7.8	3.1	14	-1.9	2.4	16	8.2%	-2.09 [-3.00, -1.18]
**Subtotal (95% CI)**			**207**			**189**	**66.4%**	**-1.33 [-2.09, -0.57]**
Heterogeneity: $Tau^2 = 1.02$; $Chi^2 = 68.42$, df = 7 (P < 0.00001); $I^2 = 90\%$								
Test for overall effect: Z = 3.42 (P = 0.0006)								
**6.15.2 No minimum BMI cut-off**								
p. Zelber-Sagi 2004	0.1	0.3	11	-0.6	0.4	12	8.0%	1.90 [0.88, 2.91]
r. Cheng 2017 (D+E)	-7.6	3.6	29	2.8	2.7	15	8.2%	-3.07 [-3.99, -2.15]
s. Dong 2016	-1.1	0.4	130	0	0.4	130	9.1%	-2.74 [-3.08, -2.40]
u. Cheng 2017 (D)	-5.4	3.3	28	2.8	2.7	14	8.3%	-2.58 [-3.45, -1.72]
**Subtotal (95% CI)**			**198**			**171**	**33.6%**	**-1.65 [-3.48, 0.18]**
Heterogeneity: $Tau^2 = 3.31$; $Chi^2 = 74.82$, df = 3 (P < 0.00001); $I^2 = 96\%$								
Test for overall effect: Z = 1.77 (P = 0.08)								
**Total (95% CI)**			**405**			**360**	**100.0%**	**-1.48 [-2.27, -0.70]**
Heterogeneity: $Tau^2 = 1.74$; $Chi^2 = 190.62$, df = 11 (P < 0.00001); $I^2 = 94\%$								
Test for overall effect: Z = 3.70 (P = 0.0002)								
Test for subgroup differences: $Chi^2 = 0.10$, df = 1 (P = 0.75), $I^2 = 0\%$								

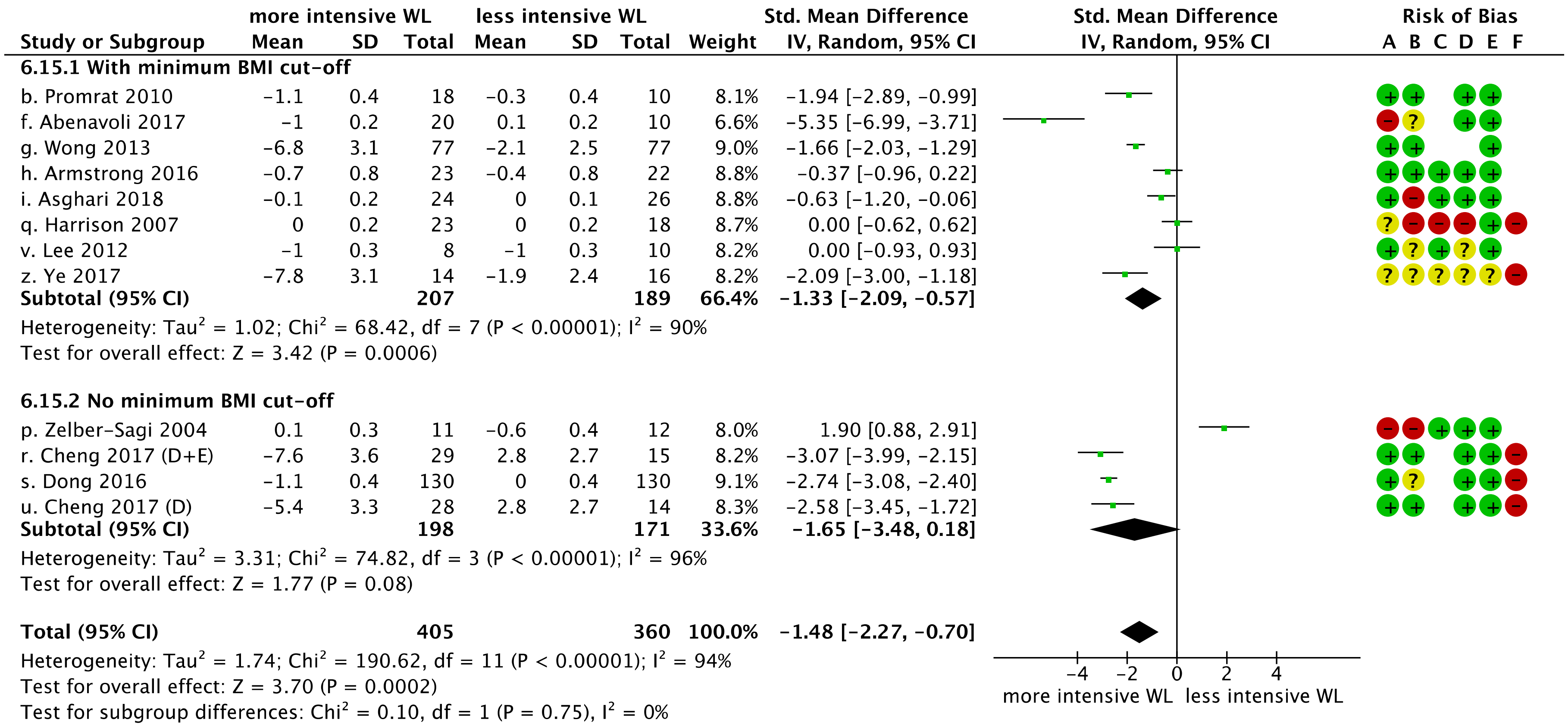

© 2019 Koutoukidis DA et al. *JAMA Internal Medicine*.

8 Subgroup analysis by the severity of the disease (NAFLD vs NASH)

eFigure 8.1 Fibrosis (stage F0-F4)

Study or Subgroup	more intensive WL Mean	SD	Total	less intensive WL Mean	SD	Total	Weight	Mean Difference IV, Random, 95% CI	Risk of Bias A	B	C	D	E	F
**7.1.1 NAFLD**														
p. Zelber-Sagi 2004	−0.7	0.6	11	−0.5	0.4	11	20.1%	−0.20 [−0.63, 0.23]	−	−	+	+	+	
w. Eckard 2013 (MF)	−0.1	1.5	9	0.1	0.5	5	9.1%	−0.20 [−1.27, 0.87]	+	?		+	+	
y. Eckard 2013 (LF)	−0.8	0.8	12	0.1	0.5	6	16.4%	−0.90 [−1.50, −0.30]	+	?		+	+	
**Subtotal (95% CI)**			**32**			**22**	**45.6%**	**−0.45 [−0.94, 0.05]**						
Heterogeneity: Tau^2 = 0.09; Chi^2 = 3.61, df = 2 (P = 0.16); I^2 = 45%														
Test for overall effect: Z = 1.77 (P = 0.08)														
**7.1.2 NASH**														
b. Promrat 2010	0	0.5	18	−0.3	0.6	10	19.8%	0.30 [−0.14, 0.74]	+	+		+	+	
h. Armstrong 2016	−0.2	0.8	23	0.2	1	22	17.9%	−0.40 [−0.93, 0.13]	+	+	+	+	+	
v. Lee 2012	0.5	0.8	8	0	0.3	10	16.8%	0.50 [−0.08, 1.08]	+	?	+	?	+	
**Subtotal (95% CI)**			**49**			**42**	**54.4%**	**0.13 [−0.38, 0.64]**						
Heterogeneity: Tau^2 = 0.14; Chi^2 = 5.95, df = 2 (P = 0.05); I^2 = 66%														
Test for overall effect: Z = 0.50 (P = 0.61)														
**Total (95% CI)**			**81**			**64**	**100.0%**	**−0.13 [−0.54, 0.27]**						
Heterogeneity: Tau^2 = 0.16; Chi^2 = 15.46, df = 5 (P = 0.009); I^2 = 68%														
Test for overall effect: Z = 0.65 (P = 0.52)														
Test for subgroup differences: Chi^2 = 2.54, df = 1 (P = 0.11), I^2 = 60.6%														

Mean Difference
IV, Random, 95% CI

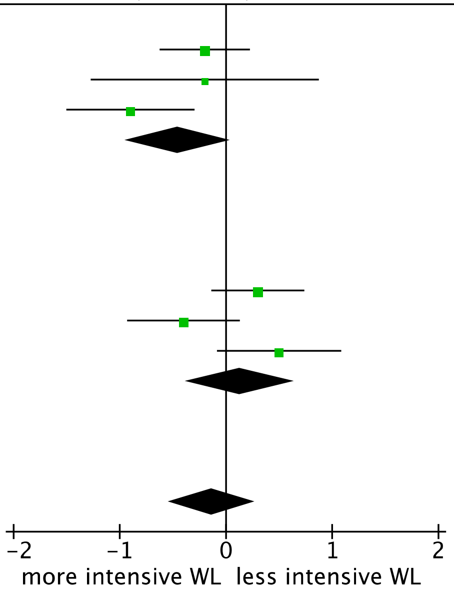

[illegible]

© 2019 Koutoukidis DA et al. *JAMA Internal Medicine*.

9 eMethods: Search strategy

Medline

	Searches
1	Non-alcoholic Fatty Liver Disease/
2	*Fatty Liver/
3	((nonalcoholic or non-alcoholic) adj5 (fatty liver or steatohepatitis)).ti,ab.
4	(fatty liver or steatohepatitis).ti.
5	(nafld or nash).ti,ab.
6	1 or 2 or 3 or 4 or 5
7	Weight Loss/
8	Weight Reduction Programs/
9	diet therapy/ or caloric restriction/ or diet, reducing/
10	exp Obesity/dh [Diet Therapy]
11	(weight adj3 (loss or lose or lost or losing or chang* or reduc* or manag*)).ti,ab.
12	((energy or calori*) adj2 (reduc* or restrict)).ti,ab.
13	((weight or overweight or obes*) adj5 (program* or service? or intervention?)).ti,ab.
14	exp Anti-Obesity Agents/
15	exp Obesity/dt, th [Drug Therapy, Therapy]
16	((weight or overweight or obes*) adj3 (therap* or treat* or drug? or agent?)).ti,ab.
17	exp OBESITY/su [Surgery]
18	exp Bariatric Surgery/
19	((weight loss or bariatric or obes*) adj5 surg*).ti,ab. or bariatric*.ti.
20	(((gastric or jejunoileal) adj3 (band* or bypass* or balloon* or diver*)) or gastrectom* or gastroplast* or ((biliopancreatic or bilio-pancreatic) adj2 diver*)).ti,ab.
21	obesity management/ or bariatrics/
22	((obes* or overweight) adj3 manage*).ti,ab.
23	7 or 8 or 9 or 10 or 11 or 12 or 13 or 14 or 15 or 16 or 17 or 18 or 19 or 20 or 21 or 22
24	randomized controlled trial.pt.
25	controlled clinical trial.pt.
26	randomized.ab.
27	placebo.ab.
28	drug therapy.fs.
29	randomly.ab.
30	trial.ab.
31	groups.ab.
32	24 or 25 or 26 or 27 or 28 or 29 or 30 or 31
33	exp animals/ not humans.sh.
34	32 not 33
35	6 and 23 and 34

© 2019 Koutoukidis DA et al. *JAMA Internal Medicine*.

Embase

	Searches
1	nonalcoholic fatty liver/
2	*Fatty Liver/
3	((nonalcoholic or non-alcoholic) adj5 (fatty liver or steatohepatitis)).ti,ab.
4	(fatty liver or steatohepatitis).ti.
5	(nafld or nash).ti,ab.
6	1 or 2 or 3 or 4 or 5
7	weight reduction/
8	weight loss program/
9	diet therapy/ or exp diet restriction/ or low calory diet/ or low fat diet/
10	exp Obesity/dm
11	(weight adj3 (loss or lose or lost or losing or chang* or reduc* or manag*)).ti,ab.
12	((energy or calori*) adj2 (reduc* or restrict)).ti,ab.
13	((weight or overweight or obes*) adj5 (program* or service? or intervention?)).ti,ab.
14	exp antiobesity agent/
15	exp Obesity/dt, th
16	((weight or overweight or obes*) adj3 (therap* or treat* or drug? or agent?)).ti,ab.
17	exp OBESITY/su
18	exp Bariatric Surgery/
19	((weight loss or bariatric or obes*) adj5 surg*).ti,ab. or bariatric*.ti.
20	(((gastric or jejunoileal) adj3 (band* or bypass* or balloon* or diver*)) or gastrectom* or gastroplast* or ((biliopancreatic or bilio-pancreatic) adj2 diver*)).ti,ab.
21	obesity management/ or bariatrics/
22	((obes* or overweight) adj3 manage*).ti,ab.
23	7 or 8 or 9 or 10 or 11 or 12 or 13 or 14 or 15 or 16 or 17 or 18 or 19 or 20 or 21 or 22
24	6 and 23
25	randomized controlled trial/
26	single blind procedure/ or double blind procedure/
27	crossover procedure/
28	random*.tw.
29	(random or ((singl* or doubl*) adj (blind* or mask*)) or crossover or cross over or factorial* or latin square or assign* or allocat* or volunteer*).ti,ab.
30	25 or 26 or 27 or 28 or 29
31	(exp animals/ or nonhuman/) not human/
32	30 not 31
33	24 and 32

© 2019 Koutoukidis DA et al. *JAMA Internal Medicine*.

PsycINFO

	Searches
1	((nonalcoholic or non-alcoholic) adj5 (fatty liver or steatohepatitis)).ti,ab.
2	(fatty liver or steatohepatitis).ti.
3	(nafld or nash).ti,ab.
4	1 or 2 or 3
5	weight loss/ or weight control/
6	(weight adj3 (loss or lose or lost or losing or chang* or reduc* or manag*)).ti,ab.
7	((energy or calori*) adj2 (reduc* or restrict)).ti,ab.
8	((weight or overweight or obes*) adj5 (program* or service? or intervention?)).ti,ab.
9	((weight or overweight or obes*) adj3 (therap* or treat* or drug? or agent?)).ti,ab.
10	bariatric surgery/
11	((weight loss or bariatric or obes*) adj5 surg*).ti,ab. or bariatric*.ti.
12	(((gastric or jejunoileal) adj3 (band* or bypass* or balloon* or diver*)) or gastrectom* or gastroplast* or ((biliopancreatic or bilio-pancreatic) adj2 diver*)).ti,ab.
13	((obes* or overweight) adj3 manage*).ti,ab.
14	5 or 6 or 7 or 8 or 9 or 10 or 11 or 12 or 13
15	4 and 14
16	random*.ti,ab,hw,id.
17	trial*.ti,ab,hw,id.
18	controlled stud*.ti,ab,hw,id.
19	placebo*.ti,ab,hw,id.
20	((singl* or doubl* or trebl* or tripl*) and (blind* or mask*)).ti,ab,hw,id.
21	(cross over or crossover or factorial* or latin square).ti,ab,hw,id.
22	(assign* or allocat* or volunteer*).ti,ab,hw,id.
23	treatment effectiveness evaluation/
24	mental health program evaluation/
25	exp experimental design/
26	(clinical trial or treatment outcome).md.
27	16 or 17 or 18 or 19 or 20 or 21 or 22 or 23 or 24 or 25 or 26
28	15 and 27

© 2019 Koutoukidis DA et al. *JAMA Internal Medicine*.

Cinahl

#	Query
S14	S3 AND S12 Limiters - Clinical Queries: Therapy - Best Balance
S13	S3 AND S12
S12	S4 OR S5 OR S6 OR S7 OR S8 OR S9 OR S10 OR S11
S11	(MH "Obesity+/DH/DT/SU/TH")
S10	TI (((weight loss or bariatric or obes*) N5 surg*)) OR AB (((weight loss or bariatric or obes*) N5 surg*)) OR TI bariatric* OR TI ((((gastric or jejunoileal) N3 (band* or bypass* or balloon* or diver*)) or gastrectom* or gastroplast* or ((biliopancreatic or bilio-pancreatic) N2 diver*))) OR AB ((((gastric or jejunoileal) N3 (band* or bypass* or balloon* or diver*)) or gastrectom* or gastroplast* or ((biliopancreatic or bilio-pancreatic) N2 diver*)))
S9	(MH "Bariatric Surgery+")
S8	TI (((weight or overweight or obes*) N3 (therap* or treat* or drug? or agent?))) AND AB (((weight or overweight or obes*) N3 (therap* or treat* or drug? or agent?)))
S7	(MH "Antiobesity Agents+")
S6	TI ((weight N3 (loss or lose or lost or losing or chang* or reduc* or manag*))) OR AB ((weight N3 (loss or lose or lost or losing or chang* or reduc* or manag*))) OR TI (((energy or calori*) N2 (reduc* or restrict))) OR AB (((energy or calori*) N2 (reduc* or restrict))) OR TI (((weight or overweight or obes*) N5 (program* or service? or intervention?))) OR AB (((weight or overweight or obes*) N5 (program* or service? or intervention?)))
S5	(MH "Diet, Reducing") OR (MH "Diet Therapy")
S4	(MH "Weight Loss") OR (MH "Weight Reduction Programs")
S3	S1 OR S2
S2	TI ("fatty liver" or steatohepatitis) OR AB (((nonalcoholic or non-alcoholic) N5 ("fatty liver" or steatohepatitis))) OR TI (nafld or nash) OR AB (nafld or nash)
S1	(MM "Fatty Liver") OR (MH "Nonalcoholic Fatty Liver Disease")

Cochrane

ID	Search
#1	MeSH descriptor: [Non-alcoholic Fatty Liver Disease] explode all trees
#2	((nonalcoholic or non-alcoholic) near (fatty liver or steatohepatitis)):ti,ab,kw or fatty liver or steatohepatitis:ti or nafld or nash:ti,ab,kw (Word variations have been searched)
#3	#1 or #2
#4	weight or obes* or overweight or bariatric*:ti,ab,kw (Word variations have been searched)
#5	MeSH descriptor: [Weight Loss] this term only
#6	MeSH descriptor: [Weight Reduction Programs] explode all trees
#7	MeSH descriptor: [Diet Therapy] this term only
#8	MeSH descriptor: [Diet, Reducing] explode all trees
#9	MeSH descriptor: [Caloric Restriction] explode all trees
#10	(weight near/3 (loss or lose or lost or losing or chang* or reduc* or manag*)):ti,ab,kw or ((energy or calori*) near/2 (reduc* or restrict)):ti,ab,kw or ((weight or overweight or obes*) near (program* or service? or intervention?)):ti,ab,kw (Word variations have been searched)
#11	MeSH descriptor: [Anti-Obesity Agents] explode all trees
#12	((weight or overweight or obes*) near/3 (therap* or treat* or drug? or agent?)):ti,ab,kw (Word variations have been searched)
#13	MeSH descriptor: [Bariatric Surgery] explode all trees

© 2019 Koutoukidis DA et al. *JAMA Internal Medicine*.

#14	bariatric*:ti or ((weight loss or bariatric or obes*) near surg*):ti,ab,kw or (((gastric or jejunoileal) near/3 (band* or bypass* or balloon* or diver*)) or gastrectom* or gastroplast* or ((biliopancreatic or bilio-pancreatic) near/2 diver*)):ti,ab,kw or ((obes* or overweight) near/3 manage*):ti,ab,kw (Word variations have been searched)
#15	MeSH descriptor: [Obesity] explode all trees
#16	MeSH descriptor: [Obesity Management] explode all trees
#17	#4 or #5 or #6 or #7 or #8 or #9 or #10 or #11 or #12 or #13 or #14 or #15 or #16
#18	#3 and #17

WoS

Set	Save search history and/or create an alertOpen a saved search history
# 9	#8 AND #7
# 8	TOPIC: ((random* or blind* or allocat* or assign* or trial* or crossover* or cross-over*))
# 7	#6 AND #1
# 6	#5 OR #4 OR #3 OR #2
# 5	TOPIC: (((obes* or overweight) NEAR/3 manage*))
# 4	TOPIC: (((weight loss or bariatric or obes*) NEAR5 surg*)) OR TOPIC: (bariatric*) OR TOPIC: ((((gastric or jejunoileal) NEAR/3 (band* or bypass* or balloon* or diver*)) or gastrectom* or gastroplast* or ((biliopancreatic or bilio-pancreatic) NEAR/2 diver*)))
# 3	TOPIC: (((weight or overweight or obes*) NEAR/3 (therap* or treat* or drug? or agent?)))
# 2	TOPIC: ((weight NEAR/3 (loss or lose or lost or losing or chang* or reduc* or manag*))) OR TOPIC: (((energy or calori*) NEAR/2 (reduc* or restrict))) OR TOPIC: (((weight or overweight or obes*) NEAR/5 (program* or service? or intervention?)))
# 1	TOPIC: (((nonalcoholic or non-alcoholic) NEAR/5 ("fatty liver" or steatohepatitis))) OR TITLE: ("fatty liver" or steatohepatitis) OR TOPIC: (nafld OR nash)

Trial Registers

**ClinicalTrials.gov**
Other terms=(obesity OR obesity OR weight OR overweight OR bariatric OR bariatrics) AND Condition=("nonalcoholic fatty liver" OR "non alcoholic fatty liver" OR "nonalcoholic steatohepatitis" OR "non-alcoholic steatohepatitis" OR nafld OR nash)
**WHO ICTRP**
nonalcoholic fatty liver AND obese OR non alcoholic fatty liver AND obese OR nonalcoholic steatohepatitis AND obese OR non-alcoholic steatohepatitis AND obese OR nafld AND obese OR nash AND obese
nonalcoholic fatty liver AND obesity OR non alcoholic fatty liver AND obesity OR nonalcoholic steatohepatitis AND obesity OR non-alcoholic steatohepatitis AND obesity OR nafld AND obesity OR nash AND obesity
nonalcoholic fatty liver AND overweight OR non alcoholic fatty liver AND overweight OR nonalcoholic steatohepatitis AND overweight OR non-alcoholic steatohepatitis AND overweight OR nafld AND overweight OR nash AND overweight
nonalcoholic fatty liver AND weight OR non alcoholic fatty liver AND weight OR nonalcoholic steatohepatitis AND weight OR non-alcoholic steatohepatitis AND weight OR nafld AND weight OR nash AND weight
nonalcoholic fatty liver AND bariatric OR non alcoholic fatty liver AND bariatric OR nonalcoholic steatohepatitis AND bariatric OR non-alcoholic steatohepatitis AND bariatric OR nafld AND bariatric OR nash AND bariatric
nonalcoholic fatty liver AND bariatrics OR non alcoholic fatty liver AND bariatrics OR nonalcoholic steatohepatitis AND bariatrics OR non-alcoholic steatohepatitis AND bariatrics OR nafld AND bariatrics OR nash AND bariatrics

© 2019 Koutoukidis DA et al. *JAMA Internal Medicine*.
